# Revisiting the origin of the bending in group 2 metallocenes AeCp_2_ (Ae = Be–Ba)†

**DOI:** 10.1039/d2cp05020j

**Published:** 2023-05-24

**Authors:** Tetiana Sergeieva, T. Ilgin Demirer, Axel Wuttke, Ricardo A. Mata, André Schäfer, Gerrit-Jan Linker, Diego M. Andrada

**Affiliations:** a Department of Chemistry, Saarland University, Campus Saarbrücken 66123 Saarbrücken Germany diego.andrada@uni-saarland.de andre.schaefer@uni-saarland.de; b Institute for Physical Chemistry, Georg-August-University Göttingen, Tammannstrasse 6 D-37077 Göttingen Germany ricardo.mata@chemie.uni-goettingen.de; c MESA+ Institute for Nanotechnology, University of Twente 7522 NB Enschede The Netherlands g.linker@utwente.nl

## Abstract

Metallocenes are well-established compounds in organometallic chemistry, and can exhibit either a coplanar structure or a bent structure according to the nature of the metal center (E) and the cyclopentadienyl ligands (Cp). Herein, we re-examine the chemical bonding to underline the origins of the geometry and stability observed experimentally. To this end, we have analysed a series of group 2 metallocenes [Ae(C_5_R_5_)_2_] (Ae = Be–Ba and R = H, Me, F, Cl, Br, and I) with a combination of computational methods, namely energy decomposition analysis (EDA), polarizability model (PM), and dispersion interaction densities (DIDs). Although the metal–ligand bonding nature is mainly an electrostatic interaction (65–78%), the covalent character is not negligible (33–22%). Notably, the heavier the metal center, the stronger the d-orbital interaction with a 50% contribution to the total covalent interaction. The dispersion interaction between the Cp ligands counts only for 1% of the interaction. Despite that orbital contributions become stronger for heavier metals, they never represent the energy main term. Instead, given the electrostatic nature of the metallocene bonds, we propose a model based on polarizability, which faithfully predicts the bending angle. Although dispersion interactions have a fair contribution to strengthen the bending angle, the polarizability plays a major role.

## Abstract 2

Metallocenes are well-established compounds in organometallic chemistry, which can exhibit either a coplanar structure or a bent structure according to the nature of the metal center (E) and cyclopentadienyl ligands (Cp).

## Graphical abstract

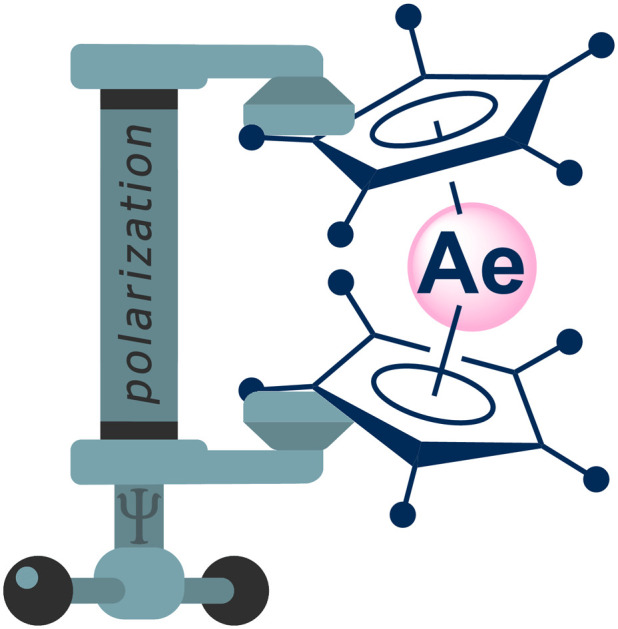

## Introduction

More than seventy years ago, Kealy, Pauson, Miller, Tebboth and Tremaine described ferrocene Fe(Cp)_2_ for the first time, laying the foundation for research on the metallocene family.^1–10^ Over the years, these compounds have evolved from only a curiosity into well-recognized reagents in organometallic chemistry, with applications ranging from coordination chemistry to homogenous catalysis and even industrial processes.^11^ To date, many examples of sandwich- or half-sandwich-type complexes with the formula E(Cp)_*n*_ (*n* = 1–4) have been prepared and structurally characterized, in which E is a main-group element or a transition metal.^11^ In particular, their structures have drawn much attention as the understanding of the bonding provides guidelines for engineering their stoichiometric and catalytic reactivity.

Attempts for modelling metallocene structures were developed parallel to the structural elucidation of ferrocene, and understanding the chemical bonding between the central atom and the cyclopentadienyl ligands is challenging using the existing heuristic models.^7–9^ Originally, the bond between the central iron atom and the Cp rings was assumed to be an electron sharing σ-type (C–Fe–C), given the lack of X-ray analysis, although the possibility of an ionic interaction ([Fe^2+^] [Cp^−^]_2_) was discussed.^1^ However, the surprisingly high thermal stability and its remarkable chemical inertness toward acids and bases could not be explained with these proposed bonding modes. Independently, Fischer^3^ and a group of scientists including Wilkinson, Rosenblum, Whiting, and Woodward^10^ proposed the metal–ligand interaction as a π-complexation. The subsequent analysis by Pfab, Eiland and Pepinsky confirmed the η^5^ binding pattern and the 6π electron aromatic character of the Cp ligands.^4^ Shortly after, Orgel used molecular orbital (MO) theory to explain the Fe–Cp bonding in ferrocene. The binding interaction was explained as formed by two “covalent–ionic” bonds resulting from the mixing of metals with Cp^−^ orbitals, and two “donor” bonds, when electrons located at the d_*x*^2^−*y*^2^_, d_*xy*_ orbitals of iron donate into vacant antibonding orbitals of the Cp ligands.^5^

The first electronic structure description was reported about 20 years after the structure elucidation.^12,13^ Although its η^5^ coordination was reproduced at the Hartree–Fock (HF) level, the Fe–C bond lengths were poorly predicted.^14^ Years later, Koch, Jørgensen, and Helgaker achieved a better theoretical structure by performing CCSD and CCSD(T) calculations.^15^ Many theoretical calculations have been reported to provide quantitative and qualitative insights into the bonding nature of metallocenes. The extension of the Dewar–Chatt–Duncanson model for ferrocene allows for the discussion of the chemical bond in terms of donor–acceptor interactions. With a strong electrostatic nature (51%), the covalent part is predicted as π-donation from MOs of the Cp^−^ ligands to the empty orbitals of the Fe^2+^ ion and a back donation from the Fe to the π* orbitals of the ligands.^16^

The replacement of Fe with main-group elements from the s- or p-block, in particular with alkaline earth metals of group 2 (Ae), or heavy elements of group 14, has attracted much attention in the light of their preferred oxidation state of +2, ever since Fischer and co-workers reported magnesocene,^17^ calcocene,^18^ stannocene,^19^ and plumbocene,^20^ just a few years after the discovery of ferrocene.^21–24^ A common observation was a more labile E–Cp bond, which was explained by the weaker π-type interaction in view of the absence of d-orbitals on the bonding. Notably, the bonding nature changes from 49% covalent in Cp_2_Fe to being predominantly ionic in the case of s-block metallocenes and slightly more covalent when the central atom is a p-block element.^16,25^

Aside from the bond nature, numerous reports attempted to explain the geometrical model of sandwich complexes. A general observation in metallocene chemistry is that structures have either a coplanar or a bent orientation of the Cp rings relative to the central atom. While the classical ferrocene Fe(Cp)_2_ and Fe(Cp*)_2_ ligands are clearly coplanar, other complexes exhibit a bent structure, *i.e.* Sn(Cp)_2_ and Pb(Cp)_2_. Such a distortion has been ascribed to the presence of a lone-pair at the central atom following the traditional valence-shell electron-pair-repulsion (VSEPR) model.^26^ However, this does not account for the subtleties of orbital interactions that influence molecular shapes and thus cannot justify bend geometries of many other complexes, the so-called non-VSEPR structures, among them are heavy alkaline earth metals (Ca, Sr, and Ba) sandwich compounds.

**Fig. 1:**
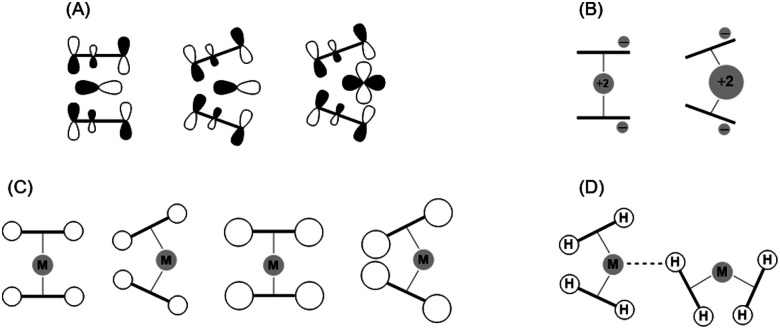
Illustration of models used to explain the bending of metallocenes. (A) Molecular orbital theory model (i). (B) Polarization model (ii). (C) Weak interaction concept (iii). (D) Agostic interaction model (iv).

Numerous models aiming at a general understanding of the structure, bonding and reactivity of such molecules have been proposed. The findings from experimental and computational investigations regarding the reason for the bending of metallocenes of heavier alkaline earth metals of group 2, which does not follow the valence shell electron pair repulsion theory model's prediction, can be categorized into four groups (Fig. 1):

(i) Molecular orbital (MO) theory model. In 1953, Walsh^27^ proposed a molecular orbital diagram and linked the angle of AB_2_ molecules as a function of molecular orbital energies. Later, Hayes addressed the bending of heavy alkaline-earth dihalides by evoking this diagram, although with some modifications.^28^ The authors suggested to take unoccupied d-orbitals of Ca, Sr and Ba into account. This was supported by the energetics of the s-, p- and d-orbitals of these elements. The d-orbitals of Be and Mg lay energetically above the level of the p-orbitals and hence do not have a strong participation in binding. Thus, the symmetry of the valence p-orbitals leads to a coplanar structure for a better orbital overlap. In contrast, the energetic arrangement of Ca, Sr and Ba orbitals is different, which results in the contribution of the d-orbitals to the Ae–Cp bonding with an optimal overlap corresponding to bent geometries.

(ii) Polarization model. Klemperer and co-workers observed a permanent electric dipole moment for monomeric dihalides of heavy alkaline earth elements, suggesting a bent arrangement.^29^ This finding was linked to the polarized-ion model, where large cations may be significantly polarized by anions due to charge–dipole and dipole–dipole interactions. In fact, these simple classical arguments were drawn by Debye to give a qualitative description for the angle in H_2_O.^30^ As such, molecular bending is related to the polarizability of the central atom and the polarizing power of the ligands. Gigli applied the polarizable Rittner type ion model to develop a quantitative prediction of dihalide dimer geometries.^31^ By splitting charge–dipole and dipole–dipole moment interactions into individual contributions, it was proposed that the major role in stabilization of linear or bent geometries is dependent on the magnitude of the induced dipole moment of the central cation. Among a large range of dipole moment values applied to the system, barium halides always showed a bent configuration, while strontium analogues adopt a linear form when the induced dipole is significantly lower.

(iii) Weak interaction concept. An alternative explanation for the bending of alkaline-earth metal complexes with Cp* ligands was discussed by Andersen *et al.*^32^ The bending energy was found to be relatively small (0.5 kcal mol^−1^ for [Ca(Cp*)_2_]) and the tilting was described in terms of maximizing the van der Waals (VdW) attractions between the methyl groups of two Cp* rings. Bosnich and co-workers re-evaluated this “weak interaction” concept and expanded the list of compounds to complexes such as SrCp*_2_, BaCp*_2_, SmCp*_2_, and EuCp*_2_.^33^ The conclusion regarding the importance of the VdW interactions was made based on the calculations of Δ*E*_VdW_, which referred to the VdW energy difference between the linear and bent geometries. The increase of Δ*E*_VdW_ values with an increase of the metal radius was ascribed to the weakening of VdW attractive forces in a linear geometry. However, Huffman and co-workers postulated that the length of intramolecular methyl-methyl contacts is in the range of 3.55–3.59 Å, regardless the radii of the central atoms (Ca, Ba, Yb, Sm, and Eu) in sandwich complex bearing Cp* ligands.^34^ One could interpret this finding in the way that metallocenes with large central cations and longer M–Cp distances should be more bent to enhance VdW interactions. It should be noted, however, that many studies do not rule out the polarizability model and consider these strengths to reinforce each other.^32,35^

(iv) Agostic interaction model. This hypothesis has been formulated relatively recently, after recognizing the importance of three-center–two-electron (3c2e) C–H⋯[E] bonds,^36^ thus an interaction between a C–H bond and a metal center with relatively high Lewis acidic character. Evidence of intermolecular agostic interactions in metallocenes of alkaline earth metals (CaCp* and BaCp*) has been invoked by Huffman as an explanation for the molecular structure and lattice pattern observed by X-ray diffraction.^34^ However, the absence of a clear trend in the packing arrangement cannot provide a clear picture of bending behaviour in the crystals. A similar conclusion was drawn by Pal *et al.*, based on the performed calculation of noncovalent interactions (NCIs) and topological analysis within the quantum theory of atoms in molecules (QTAIM) for MgCp*_2_ and CaCp*_2_ complexes.^37^ Although the geometrical, topological and NBO analysis interpretations of the C–H⋯Mg/Ca interactions to be pregostic, other forces such as VdW attraction between two Cp* rings were proposed to be the driving force of bending.

In view of the fact that the questions about the bonding situation in alkaline earth metal metallocenes were often controversially discussed because of vaguely defined concepts, we aim to address this in terms of well-established quantum chemical expressions, in the current work. The set of compounds in this study consists of unsubstituted metallocenes Ae(Cp)_2_, their methylated derivatives Ae(Cp*)_2_, and their penta-halogenated analogues Ae(C_5_R_5_), where R refers to F, Cl, Br, and I. We performed a series of analyses using energy decomposition analysis (EDA), the calculation of dispersion interaction density (DID) and polarizability to evaluate the existing concepts on the structure of metallocenes.

## Computational details

### General

Geometry optimizations were performed using the Gaussian 16 C01 software suite.^38^ The geometry optimizations for unsubstituted complexes with the general structure Ae(Cp)_2_ were carried out using density functional theory (DFT) BP86,^39–41^ B3LYP,^42,43^ M06-2X^44^ functionals with Grimme dispersion corrections D3^45^ and the Becke-Jonson damping function^46^ in combination of def2-TZVPP^47^ basis sets without any symmetry restrictions. Substituted metallocenes [Ae(C_5_R_5_)_2_] (Ae = Be-Ba and R = Me, F, Cl, Br, and I) were optimized at the B3LYP-D3(BJ)/def2-TZVPP level of theory. The stationary points were located with the Berny algorithm^48^ using redundant internal coordinates. Analytical Hessians were computed to determine the nature of stationary points (one and zero imaginary frequencies for transition states and minima, respectively)^49^ and to calculate unscaled zero-point energies (ZPEs) as well as thermal corrections and entropy effects using the standard statistical-mechanics relationships for an ideal gas.

To further evaluate bond dissociation energies (*D*_e_), single-point energy calculations using the LCCSD(T)^50–58^ methods were performed on B3LYP-D3(BJ)/def2-TZVPP optimized geometries using the program package Molpro2019.1.^59^ The cc-pVTZ basis set was used for carbon hydrogen, fluorine, chlorine and bromine, the cc-pCVTZ basis set was used for beryllium, magnesium and calcium, and the cc-pVTZ-PP^60^ basis set was used for strontium, barium and iodine.^61,62^ The LCCSD(T) calculations were carried out using Pipek-Mezey localized orbitals.^63^ The domains were determined with the use of natural population analysis criteria, with NPA = 0.03.

The natural bond orbital (NBO)^64,65^ partial charges were computed at B3LYP-D3(BJ)/def2-TZVPP using NBO 7.0.^66^

We calculate the atomic polarizability of the metal atom in the metallocenes in support of the polarizability model for the chemical angle. Single point calculations were performed, at the aforementioned optimized structures, using the local properties module LoProp^67^ of the Molcas software^68^ at the B3LYP/ANO-RCC-VTZP level of theory.^42,43^

### Energy decomposition analysis

The nature of the chemical bonds was investigated by means of the energy decomposition analysis (EDA) method, which was developed by Morokuma^69^ and Ziegler and Rauk.^70,71^ The bonding analysis focuses on the instantaneous interaction energy Δ*E*_int_ of a bond A–B between two fragments A and B in the particular electronic reference state and in the frozen geometry AB. This energy is divided into four main components (eqn (1)):

1 Δ*E*_int_ = Δ*E*_elst_ + Δ*E*_Pauli_ + Δ*E*_orb_ + Δ*E*_disp_

The term Δ*E*_elst_ corresponds to the classical electrostatic interaction between the unperturbed charge distributions of the prepared atoms (or fragments) and it is usually attractive. The Pauli repulsion Δ*E*_Pauli_ is the energy change associated with the transformation from the superposition of the unperturbed wave functions (the Slater determinant of the Kohn-Sham orbitals) of the isolated fragments to the wave function *Ψ*^0^ = *NÂ*[*Ψ*^A^*Ψ*^B^], which appropriately obeys the Pauli principle through explicit antisymmetrization (*Â* operator) and renormalization (*N* = constant) of the product wave function. It comprises the destabilizing interactions between electrons of the same spin on either fragment. The orbital interaction Δ*E*_orb_ accounts for charge transfer and polarization effects.^72^ In the case that the Grimme dispersion corrections^45,46^ are computed, the term Δ*E*_disp_ is added to the equation 1 (eqn (1)). Further details on the EDA method can be found in the literature.^73,74^ In the case of C_5_R_5_^−^, the relaxation of the fragments to their equilibrium geometries at the electronic ground state is termed Δ*E*_prep_, because it may be considered as the preparation energy for chemical bonding. The addition of Δ*E*_prep_ to the intrinsic interaction energy Δ*E*_int_ gives the total energy Δ*E*, which is – by definition with an opposite sign – the bond dissociation energy *D*_e_:

2 Δ*E*(−*D*_e_) = Δ*E*_int_ + Δ*E*_prep_

The EDA-NOCV method combines the EDA with the natural orbitals for chemical valence (NOCV) to decompose the orbital interaction term Δ*E*_orb_ into pairwise contributions. The NOCVs *Ψ*_*i*_ are defined as the eigenvector of the valence operator, *V̂*, given by equation (eqn (3)):

3 *VΨ*_*i*_ = *viΨ*_*i*_

In the EDA–NOCV scheme, the orbital interaction term, Δ*E*_orb_, is given by equation (eqn 4):4
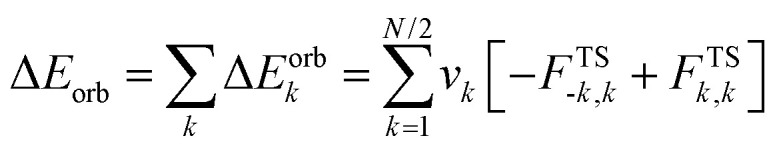
where *F*^TS^_*−k*,*−k*_ and *F*^TS^_*k*,*k*_ are the diagonal transition state Kohn–Sham matrix elements corresponding to NOCVs with the eigenvalues −*ν*_*k*_ and *ν*_*k*_, respectively. The Δ*E*^orb^_*k*_ term for a particular type of bond is assigned by the visual inspection of the shape of the deformation density Δ*ρ*_*k*_. The later term is a measure of the size of the charge deformation and it provides a visual notion of the charge flow that is associated with the pairwise orbital interaction. The EDA-NOCV scheme thus provides both qualitative and quantitative information about the strength of orbital interactions in chemical bonds. The EDA-NOCV calculations were carried out using ADF2019.101. The basis sets for all elements have triple-*ζ* quality augmented by two sets of polarization functions and one set of diffuse function. Core electrons were treated by the frozen-core approximation. This level of theory is denoted as BP86-D3(BJ)/TZ2P.^75^ Scalar relativistic effects have been incorporated by applying the zeroth-order regular approximation (ZORA).^76^

### Dispersion interaction density

Dispersion interaction densities (DIDs) were computed as proposed at the PAO-LMP2/cc-pCVDZ&cc-pVDZ level of theory.^77^ The Voxel DIDs^78^ are plotted using the ParaView Software.^79^

## Results and discussion

This section is divided as follows: first, the results and discussion on the geometry and bond dissociation of different metallocenes are presented (Section A). We then discuss the chemical bonding by dispersion interactions, and energy decomposition analysis (Section B). We finish with a model to predict the bending angle in metallocenes based on the polarizability model.

### A. Geometry and bond energies

#### Unsubstituted metallocenes [Ae(Cp)_2_]

**Fig. 2:**
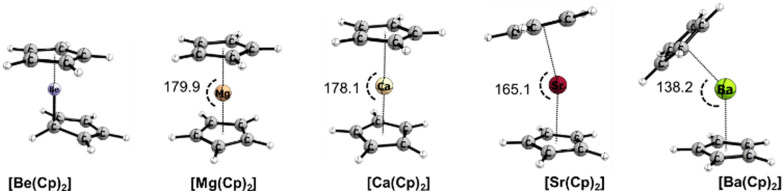
Optimized geometries of group 2 metallocenes [Ae(Cp)_2_] (Ae = Be–Ba) at the B3LYP-D3(BJ)/def2-TZVPP level of theory along with *β* angles in [°].

Fig. 2 shows the optimized structures of all complexes without symmetry constrain. When a complex is enforced coplanar, two poses are possible, namely staggered (*D*_5d_) or eclipsed (*D*_5h_). The small energetic difference within 2 kcal mol^−1^ (being the higher for [Be(Cp)_2_]) reflects the flat PES and rapid rotation of the Cp rings, which is in good agreement with previous observations.^80^ Another important aspect of these compounds is the coordination mode of the Cp rings relative to a central atom. All obtained structures shown in Fig. 2 possess the η^5^:η^5^ coordination, except Be(Cp)_2_, where a cation binds in a η^5^ manner to one Cp and η^1^ to another. This unusual “slipped sandwich” for beryllocene was previously observed by X-ray analysis^81^ and discussed in numerous theoretical studies.^82,83^ For this reason, we exclude Be(Cp)_2_ from the chemical bond discussion.

Table 1 presents the selected geometrical parameters, bond dissociation energies and natural atomic partial charges of the central element (Ae). As expected, the distances between Cp rings (geometrical centre) and central atoms are elongated with the increase of the radii of the central element in the series Mg–Ba and range from 2.00 to 2.72 Å. The values of *β* angles (Table 1) predicted magnesocene being coplanar, while strontocene and barocene are bent, regardless of the functional being used. This is in agreement with previously reported findings.^84–87^ Notably, the calcocene geometry significantly depends on the choice of the DFT level of theory. An optimization of Ca(Cp)_2_ with B3LYP leads to a coplanar structure, while BP86 and M06-2X furnished a bent structure (see the ESI,† Table S1). To examine the reliability of considered DFT methods for the prediction of calcocene bending situation, we carried out the rigid scan of PES along the Cp–Ca–Cp angle (140–180°) at the LCCSD/cc-pCVTZ&cc-pVTZ level of theory. The flat pattern of the PES (ESI,† Fig. S2) makes the estimation of functional performance to be difficult. Furthermore, the absence of experimental evidence of bending for monomeric calcocene^88^ and significant debates on this topic among theoretical reports^85,89,90^ also cannot provide an unambiguous answer regarding the bending of CaCp_2_.

**Table 1:** C_5_R_5_–Ae bond lengths, C_5_R_5_–Ae–C_5_R_5_ angles, bond dissociation energies (*D*_e_) and NBO partial charges calculated at B3LYP-D3(BJ)/def2-TZVPP and LCCSD(T)/cc-pCVTZ&cc-pVTZ {in curly brackets}. Ae = Be–Ba and R =H, Me, F, Cl, Br, and I

Ae	[Ae(Cp)_2_]	[Ae(Cp*)_2_]	[Ae(C_5_F_5_)_2_]	[Ae(C_5_Cl_5_)_2_]	[Ae(C_5_Br_5_)_2_]	[Ae(C_5_I_5_)_2_]
C_5_R_5_–Ae bond lengths, Å						
Be	—	1.649	1.635	1.639	1.656	1.676
Mg	2.000	1.959	2.028	1.987	1.981	1.983
Ca	2.351	2.315	2.367	2.337	2.336	2.328
Sr	2.542	2.500	2.559	2.520	2.524	2.524
Ba	2.722	2.687	2.731	2.702	2.713	2.718
C_5_R_5_–Ae–C_5_R_5_ angles, deg						
Be	—	179.9	179.9	179.9	179.9	179.9
Mg	179.9	179.8	180.0	179.9	179.9	179.8
Ca	178.1	158.1	150.2	151.1	154.9	163.9
Sr	165.1	149.3	155.6	145.4	148.0	153.6
Ba	138.2	139.4	142.2	138.5	142.4	148.5
*D*_e_, kcal mol^−1^^a^						
Be	713.3{724.2}	725.0{725.7}	648.1{654.8}	629.7{633.6}	625.3{626.9}	628.3{627.8}
Mg	576.8{574.1}	583.6{573.6}	506.7{502.5}	493.1{484.7}	492.7{482.0}	501.7{484.0}
Ca	501.8{500.6}	503.6{495.0}	439.3{435.3}	429.6{420.7}	428.8{418.3}	436.2{420.8}
Sr	464.7{467.2}	465.5{460.4}	406.5{406.6}	398.2{393.9}	397.3{392.0}	403.5{393.9}
Ba	437.0{441.3}	439.4{436.5}	385.0{386.0}	378.1{376.0}	376.4{374.3}	381.6{376.1}
NPA charges						
Be	+1.61	+1.72	+1.55	+1.68	+1.71	+1.74
Mg	+1.80	+1.88	+1.72	+1.81	+1.81	+1.80
Ca	+1.78	+1.79	+1.72	+1.74	+1.71	+1.67
Sr	+1.81	+1.81	+1.75	+1.77	+1.76	+1.72
Ba	+1.78	+1.77	+1.72	+1.76	+1.76	+1.74

a The dissociation energies (*D*_e_) considering the [Ae(C_5_R_5_)_2_] → Ae^2+^ + 2 C_5_R_5_^−^ dissociation.

The calculated bond dissociation energies (*D*_e_) suggest that the strength of Ae–Cp bonds is decreasing when going down the group from Mg to Ba. This finding is in agreement with the previously published data that Cp complexes of the heavier alkaline earth metals have a tendency to dissociate.^91^ Theoretically predicted partial charges at the central atoms Ae do not change within the series Mg–Ba and are determined to be approximately +1.8 au.

#### Penta-methyl-cyclopentadienyl metallocenes [Ae(Cp*)_2_]

**Fig. 3:**
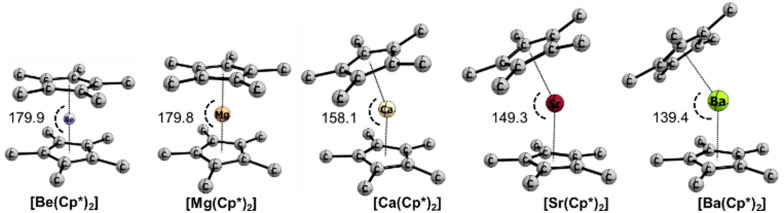
Optimized geometries of metallocenes for [Ae(Cp*)_2_] (Ae = Be–Ba) at the B3LYP-D3(BJ)/def2-TZVPP level of theory along with angles in degrees. Ae = Be–Ba and Cp* = a methylated cyclopentadienyl anion. Hydrogens are omitted for clarity.

**Fig. 4:**
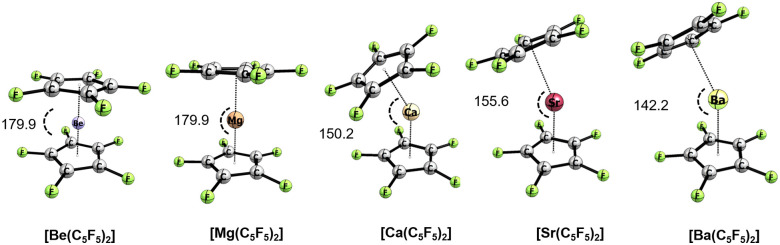
Optimized geometries of group 2 metallocenes [Ae(C_5_F_5_)_2_] (Ae = Be–Ba) at the B3LYP-D3(BJ)/def2-TZVPP level of theory along with angles in [°].

The substitution of the hydrogens by methyl groups on the Cp rings results in minor structural changes for Be, Mg, and Ba metallocenes, but significant effects can be observed for the Ca and Sr counterparts (Fig. 3 and 4). While the bond lengths Cp*–Ae in calcocene and strontocene exhibit an alteration of 0.04 Å, the bending angles become significantly more acute by *ca.* 20° to 26°. The dissociation energies *D*_e_ for all complexes calculated at B3LYP-D3(BJ) increase with respect to the Cp systems by about 10 kcal mol^−1^. However, the LCCSD(T) calculations predict similar dissociation energies. Such a difference can be due to the stronger electron-donating properties of methyl groups, which would lead to a stronger orbital interaction between Cp* and the central atom. Also, the dispersion interaction between the Cp* groups can lead to higher dissociation energies. Notably, the natural partial charges at the central atom become more positive for Be and Mg, while the heavier analogues show no differences with respect to the Cp analogues.

#### Penta-halogenated-cyclopentadienyl metallocenes [Ae(C_5_R_5_)_2_]

In order to assess the influence of the Cp substituents on the metallocene structures, we have introduced penta-halogenated cyclopentadienyl groups. Although most of the main group metallocenes have not been isolated experimentally,^21,22,24,92–94^ such a modification leads to a better understanding of the ruling electronic effect. The proposed derivatives would introduce a variety of donation properties of the Cp π-system as well as the dispersion interaction between the rings. Thus, [Ae(C_5_R_5_)_2_] complexes show similar structural features to those in the case of [Ae(Cp*)_2_] with a coplanar structure of Be and Mg, and a bent structure of Ca, Sr, and Ba. It is worth mentioning that the halogen atoms take electron density from the ring *via* an induction effect, while they donate density by a mesomeric effect. Notably, there is a trend between the π-donation strength (the resonance component of the electronic effect, *σ*^R^ = Cl > Br > I = −0.19 > −0.22 > −0.24),^95^ and the decreasing Cp–Ae–Cp angle. The higher is the ability to donate electron density to the Cp π-system the more deviation from planarity is observed. For instance, [Ca(C_5_Cl_5_)_2_] is bent by 151.1°, while [Ca(C_5_Br_5_)_2_], and [Ca(C_5_I_5_)_2_] analogues are 154.9°, and 163.9°, respectively. In contrast, tracing a correlation of bending in the C_5_F_5_ case is difficult as a result of an interplay between strong mesomeric (*R* = −0.39) and inductive effects (*F* = 0.45) for the F substituent.^95^ This trend is followed by Sr and Ba congeners (see Table 1). Alternatively, the steric clash between the R substituents might lead to the same observation. However, [Ba(C_5_I_5_)_2_] exhibits an I⋯I distance (4.442 Å) which is already longer than the sum of the van der Waals radii (3.96 Å).

Natural partial charges are comparable with those obtained for Cp and Cp*. Extreme cases such as [Be(C_5_F_5_)_2_] show a less positively charged central atoms than the rest of the molecules in the series. In terms of the bond dissociation energies *D*_e_, the halogenated systems bear between 100 and 50 kcal mol^−1^ less than Cp congeners. This suggests that the electrostatic interaction should not be strongly affected, while other physical factors might be playing a role for the weaker interaction.

### B. Chemical bonding and bending

#### Dispersion interactions

Among the models for alkaline-earth metallocene structures, it is well-recognized that the dispersion interaction between the Cp groups enforces to bend their structures when the central metal is polarizable enough.^37^ Calcocene has been in the spotlight of discussion since the theoretical structures display a sharp dependence on the Cp substituents. Specifically, the dispersion interaction between methyl groups of Cp* rings has been ascribed as the responsible factor for bending the structure from 178.1° to 158.1° (Table 1). However, some polarization can also play a role since d-orbitals are significantly populated.^96^ To sort these interactions, Grabowsky and co-workers optimized [Ca(Cp*)_2_] structures at the B3LYP level of theory with and without the dispersion term. The results showed that B3LYP-D3 favours a bent geometry with an energetic preference of ∼1 kcal mol^−1^, while excluding dispersion leads to a coplanar structure. In this regard, the so-called “dispersion theory”, Kaltsoyannis and Russo suggested a weak interaction between two Cp* rings, then (Cp*)_2_^2−^ should also be a bent structure, if the nature of the central metal is not important.^97,98^

**Fig. 5:**
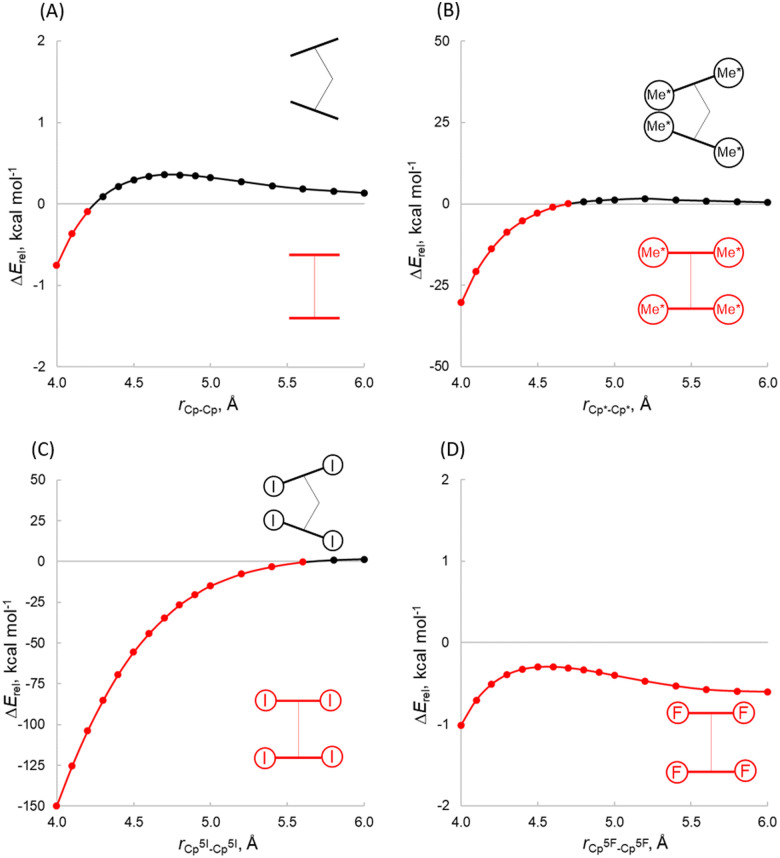
Rigid potential energy surface scans of planar *vs.* bent metalloceneanions (Cp)_2_^2−^ as a function of the Cp^X^–Cp^X^ distance performed at the B3LYP-D3(BJ)/def2-TZVPP level of theory (Δ*E*_rel_ = *E*_bent_ − *E*_linear_).

In the absence of the centre metal, we have computed the energy between the C_5_R_5_ groups in two different geometries, bent (140°) and coplanar (180°), at different distances (Fig. 5A and B). While the Cp system shows a lower energy for the coplanar structure at short distances, at longer distances the bent structure becomes more favourable. Notably, in the case of Cp*, the coplanar structure is more stable at short distances, while at longer distances the bent structure sets in (at 4.7 Å) since the energy penalty becomes negligible. These results indicate that the cation is necessary for a bent structure where the dispersion interaction might not be the main determinant of the distortion. To expand aforementioned ideas, we performed a rigid scan of PES for planar (180°) *vs.* bent (140°) (C_5_R_5_)_2_^2−^ structures at different distances Cp^X^(centroid)–Cp^X^(centroid) ranging from 4.0 to 6.0 Å (Fig. 5C and D), which covers the range of atomic radii considered. Firstly, the preference of (C_5_F_5_)_2_^2−^ to be coplanar at any point of the curve (Fig. 5D), which is in sharp contrast to the behaviour of [Ae(C_5_F_5_)_2_] (Table 1), indicating that the presence of the central atom has an important role. The same conclusion can be outlined analysing the trends for (C_5_I_5_)_2_^2−^. In contrast, (Cp)_2_^2−^ and (Cp*)_2_^2−^ indeed behave very similarly to analogous [Ae(Cp)_2_] and [Ae(Cp*)_2_] complexes, now suggesting that the central atom has a lower impact on the bending. Furthermore, an inspection of plots (Fig. 5B) reveals that the bending of (Cp*)_2_^2−^ strongly depends on the distance between aromatic rings. (Cp*)_2_^2−^ is bent when the distance is 4.8–6.0 Å, while planar at 4.0–4.7 Å. This finding contradicts a Kaltsoyannis argument about the necessity of the cation for bending, since the model they utilized was limited to specific Cp^X^–Cp^X^ lengths. At this point, one cannot exclude the dispersion to be a driving force of the bending.

**Fig. 6:**
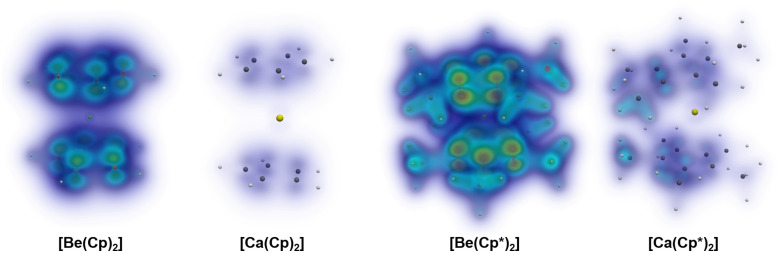
Dispersion interaction density (DID) plots calculated at the PAO-LMP2/cc-pCVTZ&cc-pVTZ level of theory. The brown zones indicate regions of electron density in a molecule which interacts strongly by dispersion interactions with the other molecule. Blue stands for weaker/diffuse contributions.

From the optimised structures, we calculated at the PAO-LMP2/cc-pCVTZ&cc-pVTZ level the dispersion interactions between the C_5_R_5_ rings (R = H and Me). Given the local character of occupied and virtual orbitals in the local correlation treatments, the intermolecular effects due to double excitations from occupied orbitals of one unit into virtual orbitals of the same unit and intermolecular effects due to excitations involving orbitals from both units can be divided. Additionally, the interactions (Cp⋯Cp) can be dissected into dispersion effects, exchange dispersion, and ionic contributions.^99–101^Table 2 provides the results of the energy decomposition of the local correlation approach, and the exemplary dispersion interaction density (DID)^77^ profiles of Be(Cp)_2_, Be(Cp*)_2_, Ca(Cp)_2_ and Ca(Cp*)_2_ are shown in Fig. 6. As expected, the dispersion interaction between the rings is stronger for the beryllium complexes and drastically diminishes with the heavier analogues. The Cp* system doubles the amount of the dissection interaction in Cp systems, in good agreement with the higher dissociation energy values. The DID profile shows that the π–π interaction between the rings dominates the dispersion profile in most cases, but for Cp* starting from Ca the close C–H contacts rule the interaction.

**Table 2:** Energy decomposition within the local correlation treatment for [Ae(C_5_R_5_)_2_] (R = H and Me) at PAO-LMP2/cc-pCVTZ&cc-pVTZ. Values are in kcal mol^−1^

	Δ*E*_intra_	Δ*E*_disp ex_	Δ*E*_disp_	Δ*E*_ionic_
Be(Cp)_2_	−18.1	−0.1	−10.8	−7.2
Mg(Cp)_2_	−6.3	0.0	−4.5	−1.8
Ca(Cp)_2_	−3.8	0.0	−2.8	−1.0
Sr(Cp)_2_	−2.6	0.0	−2.0	−0.6
Ba(Cp)_2_	−9.2	0.0	−2.2	−7.0
Be(Cp*)_2_	−30.6	−0.1	−20.5	−9.9
Mg(Cp*)_2_	−12.4	0.0	−9.4	−2.9
Ca(Cp*)_2_	−11.2	0.0	−6.7	−4.5
Sr(Cp*)_2_	−7.9	0.0	−4.9	−3.0
Ba(Cp*)_2_	−39.0	0.0	−4.9	−34.1

#### Molecular orbitals and polarizability

What is the role of the central metal? An alternative explanation of the metallocene bending relates to the type and shape of the orbitals participating in bond formation. According to this concept, beryllocene and magnesocene are coplanar, which results from the engagement of only valence s and p orbitals for Be and Mg into σ bonding with ligands. However, the involvement of d-orbitals in the case of Ca, Sr, Ba induces bending.

**Fig. 7:**
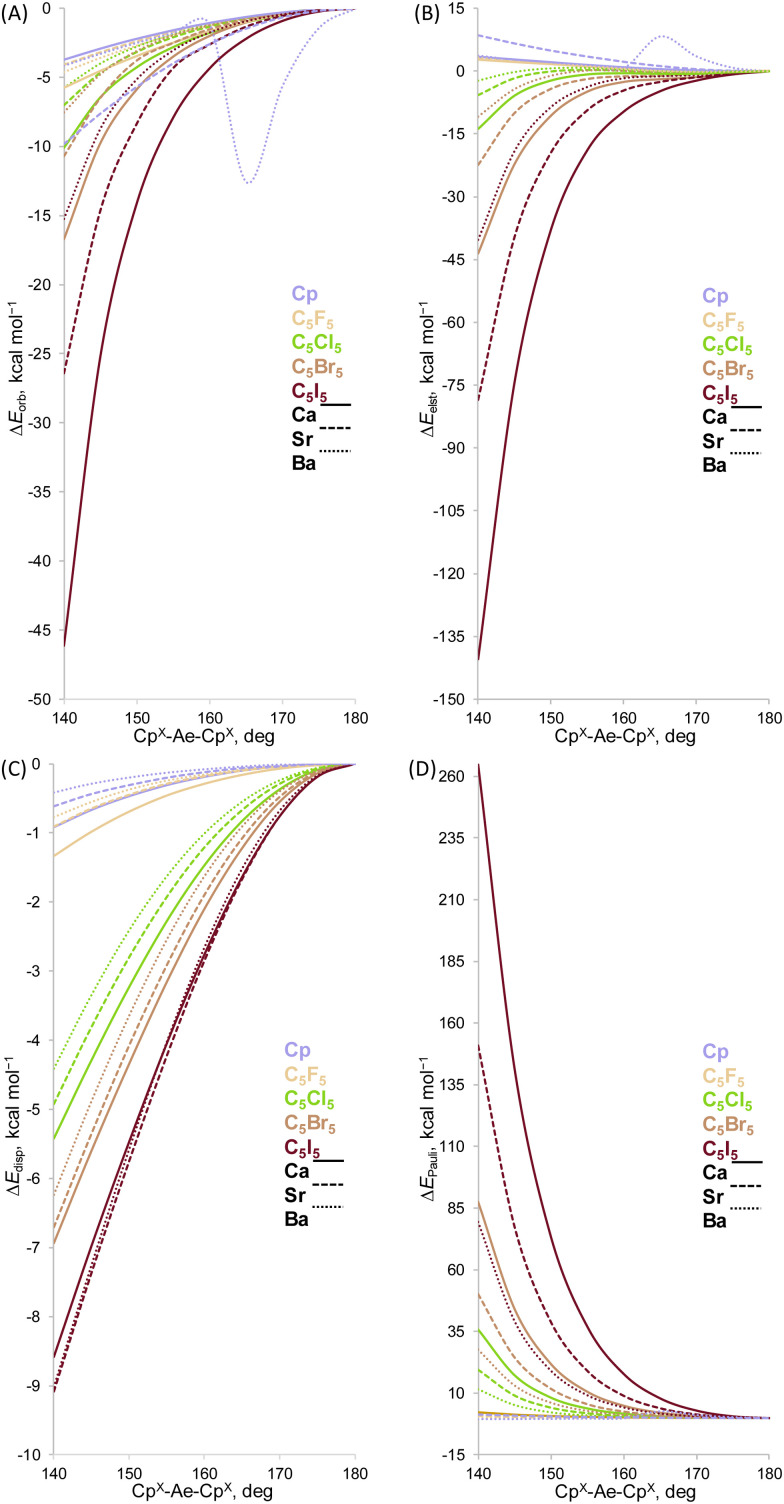
Energy decomposition analysis at the BP86-D3(BJ)/TZ2P level of theory for the [Ae(C_5_R_5_)_2_] complexes. Ae = Be–Ba and R = H, Me, and F–I. Energy values are given in kcal mol^−1^. (A) Orbital interaction, (B) electrostatic interaction, (C) dispersion interaction, and (D) Pauli repulsion.

To gain a quantitative insight into the role of the main orbital interaction, we performed the energy decomposition analysis (EDA) of the complexes maintaining the same C_5_R_5_–Ae distance given by the full optimization but changing the Cp^X^–Ae–Cp^X^ angle from 140 to 180°. The EDA is a useful tool to assess the nature of the chemical bond and to identify the driving forces behind the binding interaction.^102^ The nature of the energy components has been a matter of debate, given the path-dependent nature.^103–105^ However, the fragmentation schemes used in a consistent manner generate reliable trends.^106,107^ We have used three fragment schemes to take into account the interaction between the Cp^–^ moieties. The result of splitting Δ*E*_int_ is shown in Fig. 7. Inspection of the EDA terms revealed that the trend in stabilizing bent structures by Δ*E*_orb_, Δ*E*_disp_ and Δ*E*_elst_ terms counteract by the Δ*E*_Pauli_ forcing metallocenes to adopt the coplanar configuration and the interplay between these attractive and repulsive components determines the extent of deformation. Notably, the electrostatic interaction favours a coplanar structure for most of the metallocenes, with the exception of Cp. This finding shows that the previously controversial orbital interaction and dispersion in fact accompany each other promoting a bent structure. Note, however, that the dispersion interaction represents a minor factor (1%) in comparison to the orbital interaction.

**Fig. 8:**
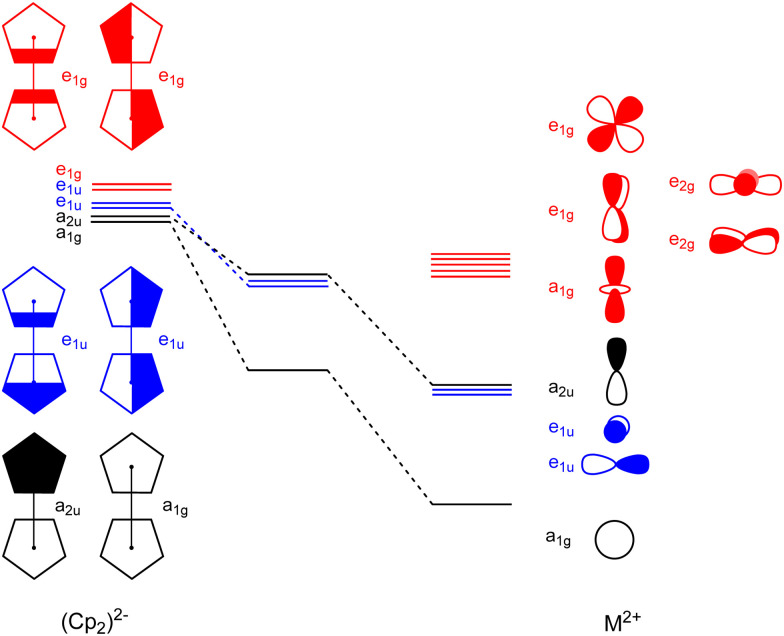
Schematic orbital correlation diagram for the coplanar complex Mg(Cp)_2_.

**Fig. 9:**
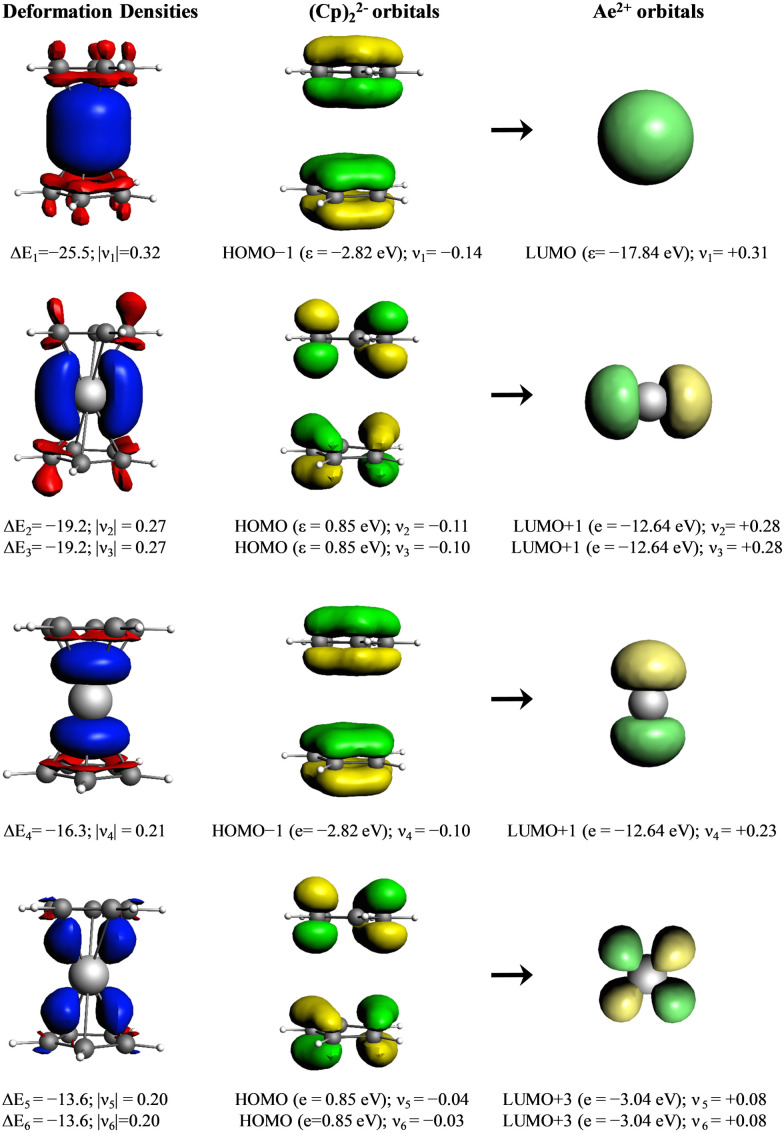
Plot of the deformation densities Δ*ρ* of the pairwise orbital interactions between Mg^2+^ in its A1^0^ electronic state and (Cp_2_)^2−^, associated energies Δ*E* (in kcal mol^−1^) and eigenvalues ν (in a.u.). The red color shows the charge outflow, whereas the blue color shows the charge density accumulation. The shape of the most important interacting occupied and vacant orbitals of the fragments.

Deeper insights into the nature of the orbital interaction are available from the combination of EDA with natural orbitals for chemical valence calculations (EDA-NOCV).^108,109^ This method deconstructs the orbital term (Δ*E*_orb_) into components (Δ*E*_orb_*ρ*(*i*)) that provide an energetic estimation of a given deformation density (*ρ*(*i*)), which is related to a particular electron flow channel, and consequently the amount of charge transferred, Δ*q*_(*i*)_ = |*ν*_(*i*)_|, for the bonding between the interacting fragments. The most interesting results are obtained by breaking down the orbital term Δ*E*_orb_. Fig. 8 shows the orbital diagram for the most important orbital interaction in a coplanar situation (*D*_5d_). In this situation, s and d_*z*^2^_ can interact with the a_1g_ combination of the C_5_R_5_ rings. The p orbitals interact with the e_1u_ and a_2u_ orbitals, while the remaining d orbitals can interact with the e_1g_ C_5_R_5_ orbitals. Fig. 9 represents the shape of the deformation densities *ν*_1_–*ν*_6_, showing the charge flow and the most important fragment orbitals which are involved in the pairwise donor–acceptor bonding for the MgCp_2_ metallocene complexes. The scheme in Fig. 8 can be associated with the interactions displayed in Fig. 9. The color coding red to blue illustrates the direction of the charge flow. EDA-NOCV reveals that the major interaction originates from the participation of s and p orbitals, which is because the symmetry keeps the complexes co-planar. When going down in the group from Mg to Ba, the contribution of d-orbitals is increasing triggering the sandwiches to bent their geometries (ESI,† Table S2 and Fig. S3–S7). For instance, the acceptor contributions of the formally empty d orbital to the total orbital interaction are Mg(Cp)_2_ (19.2%), Ca(Cp)_2_ (49.5%), Sr(Cp)_2_ (46.9%), and Ba(Cp)_2_ (47.2%). With the contribution of the d orbitals in addition to the small contribution of the dispersion interactions, we benchmarked the model based on the polarizability of the central atom.

#### Polarizability model

**Fig. 10:**
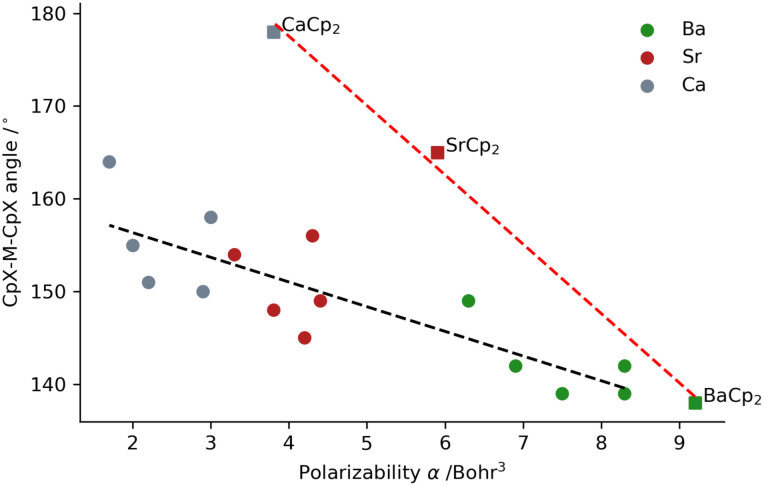
Cp^X^–Ae–Cp^X^ angle depending on the polarizability of the constituent metal centre atom, where Ae = Ca, Sr, and Ba and Cp^X^ is the indication of the H substitution in the Cp molecules (see the text).

Given the increasing importance of the d-orbital involvement and associated deformation densities in metallocenes with the heavier metal center, and hence its polarizability, we addressed a model that can reproduce the experimental observation. From the extended Debye polarizability (EDP) model,^110–112^ it follows that bending can be initiated when the electron density around the metal center atom (Ae) is sufficiently polarizable. As an extension of Debye's model for H_2_O,^30^ the EDP model provides a more balanced description by treating the centre and outer atoms equally in allowing induced dipoles not only at the centre atom but also at the ligands. For bent geometries, the EDP model predicts that the larger Ae polarizability gives rise to a smaller Cp–Ae–Cp angle. The NBO charge on the metal center inside the metallocene is estimated to be in the range of +1.6 to +1.8 (Table 1). The occupancy of the valence s shell of the metal will be almost depleted. The polarizability around Ae in the metallocenes is expected to be small for Be and Mg, but in the series Ae = Ca, Sr, and Ba we expect some polarizability to originate from sub-valence electrons. The optimized geometries of group 2 metallocenes (Table 1) provide a way to test the dependence of the angle on the polarizability of the centre atom. Omitting the linear Be and Mg centered metallocenes, we calculate the polarizability around Ae in the Ca, Sr and Ba centered metallocenes. In Fig. 10, the C_5_R_5_–Ae–C_5_R_5_ angle is plotted against the calculated Ae polarizability. We broadly see the predicted trend towards sharper angles with a higher polarizability around the center atom. The trend line does not infer a linear trend *per se*, and is only intended to guide. Compared to the substituted Ae(C_5_R_5_)_2_, a series of unsubstituted metallocenes Ae(Cp)_2_ seem to have a stronger dependence on the polarizability following the angles Ca (178°) > Sr (165°) > Ba (138°). This is related to the different trend computed by the EDA analysis (Fig. 7), where the electrostatic interaction favours the bending.

## Conclusions

In this work, we have addressed the long-standing dichotomy of the experimentally observed metallocene coplanar and bent structures. Under (quasi) gas phase conditions, the lightest group 2 metallocenes such as Be and Mg exhibit a coplanar arrangement of the Cp ligands, while the geometry becomes bent for heavier atoms such as Ca, Sr and Ba. Our calculation of the interaction of the C_5_R_5_ rings in the absence of the central atom suggests a coplanar structure as the most favourable at short distances, while at longer distances the coplanar and bent structures are energetically similar. This is a consequence of a strong interaction between the π systems of the rings. Nonetheless, the energy decomposition analysis suggests a strong ionic character (*ca.* 70%) of the bond between the metal and Cp and only a minor role of dispersion interactions, representing only about 1% of the total stabilizing interactions. The further dissection of the orbital term reveals six main orbital contributions which consist of donations of the different π-orbitals into the s, p and d orbitals on the metal centers. Notably, the contribution of the d orbitals becomes dominant for Ca, Sr and Ba. Thus, the strong d-orbital dependence and associated charge deformations give prevalence to the notion, put forward originally by Debye, that the ability to form induced dipoles at the centre atom is connected to the stabilization of the bent molecule. In this manner, the bending angle can be accurately assessed by the polarizability of the central Ae2+ atom. Exactly how the angle changes with the polarizability will depend on the ligand, as it influences the ratio of the different forces at play (*e.g.*, Pauli repulsion and electrostatic interactions). The clearest example within the complexes studied is itself the cyclopentadienyl ring derivatives [Ae(Cp)_2_], which exhibit less Pauli repulsion and favorable electrostatic interactions.

## Author contributions

T. S., T. I. D., A. W., G.-J. L., and D. M. A. performed the calculations. G.-J. L., R. A. M., A. S., and D. M. A. acquired funding and contributed methodologies. T. S., T. I. D., A. S., G.-J. L., and D. M. A. prepared the manuscript and the ESI.† All authors contributed to the interpretation of the computed data and the writing and editing of the manuscript.

## Conflicts of interest

There are no conflicts to declare.

## Supplementary Material

CP-025-D2CP05020J-s001
