# Supplementary material for: Revisiting the origin of the bending in group 2 metallocenes AeCp2 (Ae = Be–Ba)
Source: Phys Chem Chem Phys. 2023 May 24;25(30):20657–67. doi: 10.1039/d2cp05020j (PMC10395002; doi:10.1039/d2cp05020j)
Supplement: CP-025-D2CP05020J-s001 [file CP-025-D2CP05020J-s001.pdf]

Electronic Supplementary Material (ESI) for Physical Chemistry Chemical Physics.
This journal is © the Owner Societies 2023

Supporting Information

for

Revisiting the Origin of the Bending in Group 2 Metallocenes Cp_2Ae (Ae = Be - Ba)

Tetiana Sergeieva,[a] T. Ilgin Demirer,[a] Axel Wuttke,[b] Ricardo A. Mata,*[b] and André Schäfer,*[a] Gerrit-Jan Linker,*[c] and Diego M. Andrada,*[a]

[a] *Institut für Allgemeine und Anorganische Chemie, Universität des Saarlandes, D-66123 Saarbrücken, Germany.*

[b] *Institut für Physikalische Chemie, Georg-August-Universität Göttingen, Tammannstrasse 6, D-37077 Göttingen, Germany.*

[c] *MESA+ Institute for Nanotechnology, University of Twente, 7522 NB Enschede, The Netherlands.*

Table of Content

**Geometry and Bond Dissociation Energies**	**S3**
**Energy Decomposition Analysis**	**S6**
**Orbital Scheme**	**S33**
**Electronic Energy and xyz coordinates**	**S34**

Geometry and Bond Dissociation Energies

Table S1. Geometrical Parameters (in Å and °) and bond dissociation energy (D_e in kcal/mol) at the BP86-D3(BJ)/def2-TZVPP and M06-2X/def2-TZVPP (in parenthesis) level of theory.[a,b]

$[Ae(C_5R_5)_2]$	**Distance C_5R_5-Ae**	**Angle C_5R_5-Ae-C_5R_5**	***D_e*[a]**
$[Be(Cp)_2]$	1.649 (1.642)	179.9 (180.0)	707.2 (714.5)
$[Mg(Cp)_2]$	2.000 (1.982)	179.6 (179.6)	572.6 (581.1)
$[Ca(Cp)_2]$	2.332 (2.340)	163.6 (163.5)	503.7 (502.4)
$[Sr(Cp)_2]$	2.514 (2.524)	150.0 (149.9)	467.4 (468.7)
$[Ba(Cp)_2]$	2.691 (2.704)	131.4 (136.9)	442.0 (441.9)
$[Be(Cp^*)_2]$	1.645 (1.628)	180.0 (179.9)	721.6 (729.4)
$[Mg(Cp^*)_2]$	1.957 (1.953)	180.0 (180.0)	578.6 (584.2)
$[Ca(Cp^*)_2]$	2.296 (2.320)	154.0 (151.7)	506.8 (500.8)
$[Sr(Cp^*)_2]$	2.477 (2.503)	148.8 (143.4)	469.3 (465.3)
$[Ba(Cp^*)_2]$	2.661 (2.671)	139.1 (137.1)	445.2 (439.5)

[a] The dissociation energies (De) considering the $[Ae(C_5R_5)_2] \rightarrow Ae^{2+} + 2\ C_5R_5^{-}$ dissociation. [b] All dissociation energy values are counterpoise corrected.

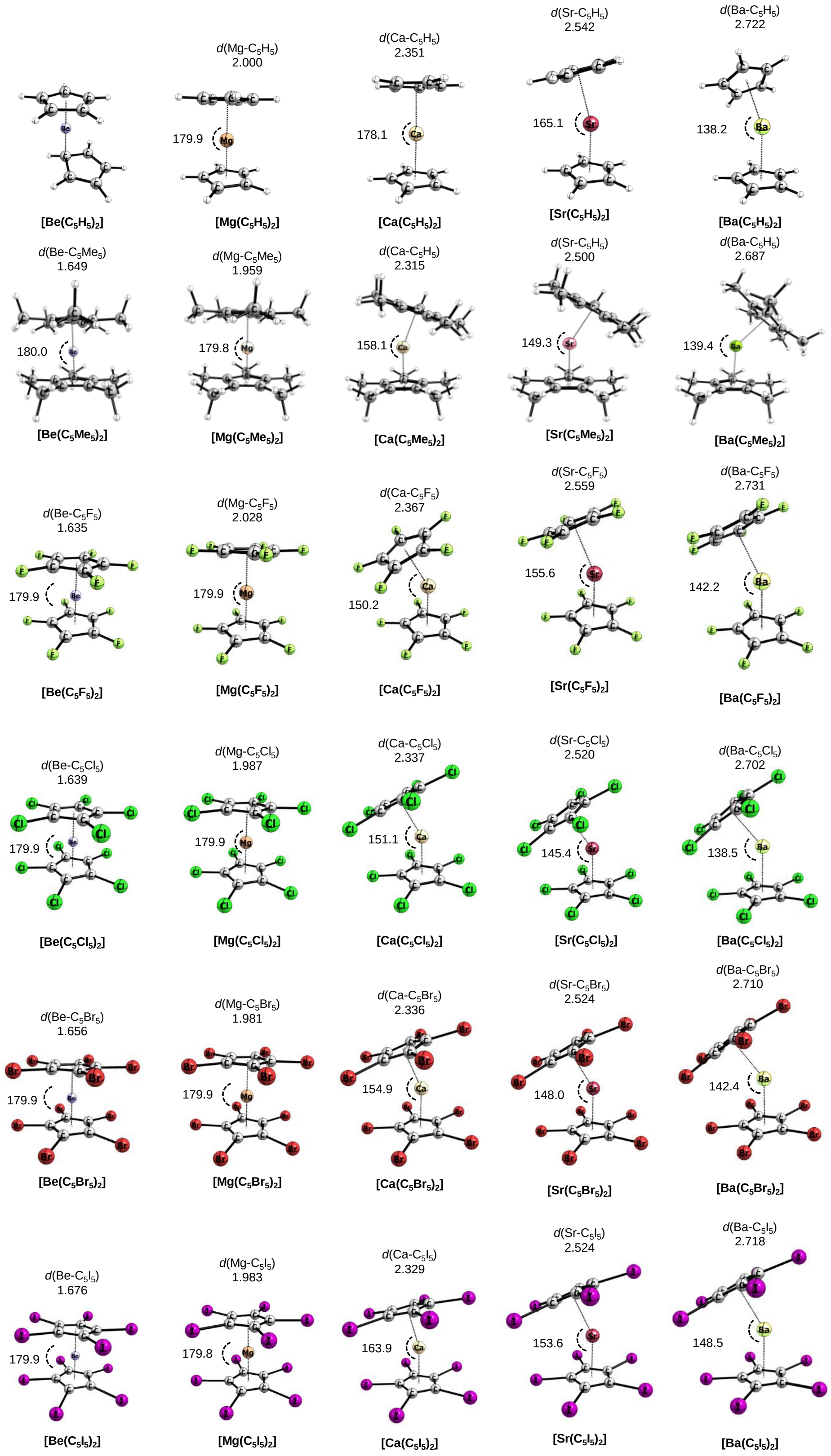

Figure S1. Optimized geometries of Group 2 metallocenes [$Ae(C_5R_5)_2$] (Ae = Be-Ba, R = H, Me, F, Cl, Br, I) at the B3LYP-D3(BJ)/def2-TZVPP level of theory.

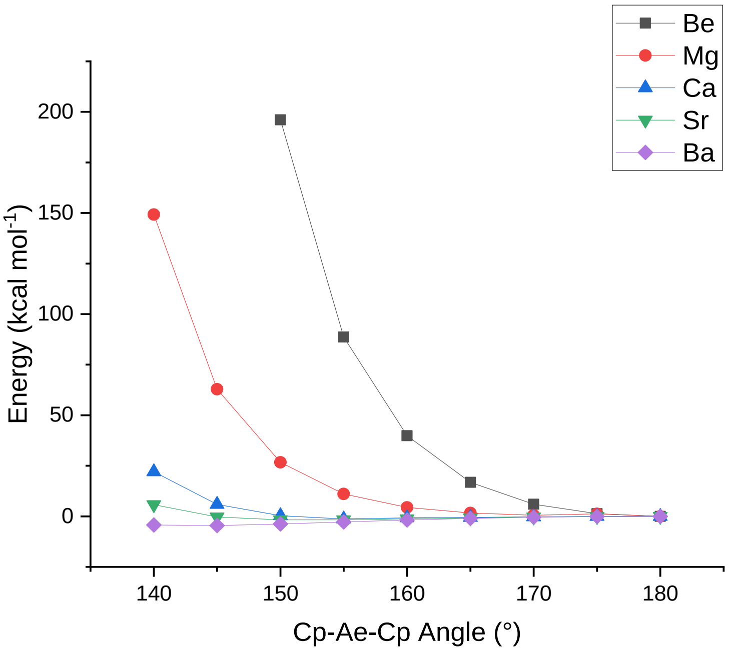

Figure S2. Electronic Energy *vs* bending angle of Group 2 metallocenes [Ae(Cp)$_2$] (Ae = Be-Ba) at the LCCSD(T)/cc-pCVTZ&cc-pVTZ//B3LYP-D3(BJ)/def2-TZVPP level of theory. The energy is relative to the structure at 180°.

Energy Decomposition Analysis

Table S2. Energy decomposition analysis at the BP86-D3(BJ)/TZ2P level of theory for the $[Ae(C_5R_5)_2]$ (Ae = Be-Ba, R =H, Me, F, Cl, Br, I) complexes. Energy values are given in kcal/mol.

	$[Be(Cp)_2]$	$[Mg(Cp)_2]$	$[Ca(Cp)_2]$	$[Sr(Cp)_2]$	$[Ba(Cp)_2]$
Fragments	Be^{2+} (s^0); $2Cp^−$ (a'^{32} a''^{20})	Mg^{2+}(s^0); $2Cp^−$ (a'^{32} a''^{20})	Ca^{2+}(s^0); $2Cp^−$ (a'^{32} a''^{20})	Sr^{2+}(s^0); $2Cp^−$ (a'^{32} a''^{20})	Ba^{2+}(s^0); $2Cp^−$ (a'^{32} a''^{20})
ΔE_{int}	-706.0	-569.7	-503.0	-466.0	-440.7
ΔE_{Pauli}	81.5	74.8	97.1	96.9	105.5
ΔE_{elstat}[a]	-517.8 (65.8 %)	-494.0 (76.7 %)	-465.5 (74.2 %)	-446.2 (79.3 %)	-428.3 (78.4 %)
ΔE_{orb}[a]	-260.7 (33.1 %)	-142.1 (22.1 %)	-126.1 (20.1 %)	-108.1 (19.2 %)	-108.3 (19.8 %)
ΔE_{disp}[a]	-9.0 (1.1 %)	-8.3 (1.3 %)	-35.4 (5.7 %)	-8.6 (1.5 %)	-9.6 (1.8 %)
$\Delta E_{\rho 1}$(a' → p_x)[b]	-53.6 (20.6 %)	-19.2 (13.5 %)	-8.1 (6.4 %)	-7.5 (6.9 %)	-10.2 (9.4 %)
$\Delta E_{\rho 2}$ (a''→ p_y)[b]	-53.8 (20.6 %)	-19.1 (13.4 %)	-8.2 (6.5 %)	-7.5 (6.9 %)	-7.4 (6.8 %)
$\Delta E_{\rho 3}$ (a'→ p_z)[b]	-44.5 (17.1 %)	-16.3 (11.5 %)	-6.2 (4.9 %)	-4.6 (4.3 %)	-3.8 (3.5 %)
$\Delta E_{\rho 4}$(a' →s)[b]	-40.5 (15.5 %)	-25.6 (18.0 %)	-15.6 (12.4 %)	-15.7 (14.5 %)	-18.9 (17.5 %)
$\Delta E_{\rho 5}$ (a'→ d_{xz})[b]	-12.7 (4.9 %)	-13.6 (9.6 %)	-31.5 (25.0 %)	-26.0 (24.1 %)	-24.8 (22.9 %)
$\Delta E_{\rho 6}$ (a'→ d_{yz})[b]	-12.7 (4.9 %)	-13.6 (9.6 %)	-30.9 (24.5 %)	-24.7 (22.8 %)	-22.4 (20.7 %)
ΔE_{orb}(rest)[b]	-42.9 (16.4 %)	-34.7 (24.4 %)	-25.6 (20.3 %)	-22.1 (20.4 %)	-20.8 (19.2 %)
ΔE_{prep}	0.6	0.3	0.5	0.5	0.4
$-D_e$	-705.4	-569.4	-502.5	-465.5	-440.3
	$[Be(Cp^*)_2]$	$[Mg(Cp^*)_2]$	$[Ca(Cp^*)_2]$	$[Sr(Cp^*)_2]$	$[Ba(Cp^*)_2]$
Fragments	Be^{2+} (s^0); $2Cp^{*-}$ (a'^{32} a''^{20})	Mg^{2+}(s^0); $2Cp^{*-}$ (a'^{32} a''^{20})	Ca^{2+}(s^0); $2Cp^{*-}$ (a'^{32} a''^{20})	Sr^{2+}(s^0); $2Cp^{*-}$ (a'^{32} a''^{20})	Ba^{2+}(s^0); $2Cp^{*-}$ (a'^{32} a''^{20})
ΔE_{int}	-725.1	-581.2	-511.0	-472.6	-449.3
ΔE_{Pauli}	93.5	88.5	112.5	110.1	116.8
ΔE_{elstat}[a]	-503.9 (61.5 %)	-478.2 (71.4 %)	-447.4 (71.3 %)	-428.2 (73.5 %)	-411.3 (72.7 %)
ΔE_{orb}[a]	-291.7 (35.6 %)	-171.9 (25.7 %)	-156.9 (25.0 %)	-135.6 (23.3 %)	-134.3 (23.7 %)
ΔE_{disp}[a]	-23.1 (2.8 %)	-19.6 (2.9 %)	-19.2 (3.1 %)	-18.9 (3.2 %)	-20.4 (3.6 %)
$\Delta E_{\rho 1}$(a' → p_x)[b]	-56.2 (19.3 %)	-21.1 (12.3 %)	-9.7 (7.7 %)	-8.6 (6.3 %)	-10.9 (8.1 %)
$\Delta E_{\rho 2}$ (a''→ p_y)[b]	-55.9 (19.2 %)	-21.0 (12.2 %)	-9.1 (7.2 %)	-8.2 (6.1 %)	-7.9 (5.9 %)
$\Delta E_{\rho 3}$ (a'→ p_z)[b]	-44.8 (15.4 %)	-16.6 (9.7 %)	-6.0 (4.8 %)	-4.4 (3.2 %)	-3.5 (2.6 %)

$\Delta E_{\rho 4}$(a' → s)[b]	-40.7 (14.0 %)	-25.7 (15.0 %)	-18.5 (14.7 %)	-17.6 (12.9 %)	-20.3 (15.1 %)
$\Delta E_{\rho 5}$ (a' → d_{xz})[b]	-12.9 (4.4 %)	-14.9 (8.7 %)	-35.1 (27.8 %)	-29.1 (21.5 %)	-28.2 (21.0 %)
$\Delta E_{\rho 6}$ (a' → d_{yz})[b]	-12.9 (4.4 %)	-14.8 (8.6 %)	-33.3 (26.5 %)	-27.4 (20.2 %)	-25.2 (18.8 %)
ΔE_{orb}(rest)[b]	-68.3 (23.4 %)	-40.7 (33.6 %)	-45.2 (35.9 %)	-40.3 (29.7 %)	-38.3 (28.5 %)
ΔE_{prep}	1.7	1.8	1.6	1.6	1.3
$-D_e$	-723.4	-579.4	-509.4	-471.0	-448.0
	$[Be(C_5F_5)_2]$	$[Mg(C_5F_5)_2]$	$[Ca(C_5F_5)_2]$	$[Sr(C_5F_5)_2]$	$[Ba(C_5F_5)_2]$
Fragments	$Be^{2+}(s^0)$;2$(C_5F_5)^-$ (a'^{32} a''^{20})	$Mg^{2+}(s^0)$; 2$(C_5F_5)^-$ (a'^{32} a''^{20})	$Ca^{2+}(s^0)$; 2$(C_5F_5)^-$ (a'^{32} a''^{20})	$Sr^{2+}(s^0)$; 2$(C_5F_5)^-$ (a'^{32} a''^{20})	$Ba^{2+}(s^0)$; 2$(C_5F_5)^-$ (a'^{32} a''^{20})
ΔE_{int}	-650.6	-503.4	-444.4	-409.4	-388.6
ΔE_{Pauli}	74.6	64.9	81.58	76.4	81.9
ΔE_{elstat}[a]	-419.49 (57.8 %)	-403.4 (71.0 %)	-376.2 (71.5 %)	-360.7 (74.2 %)	-347.5 (73.9 %)
ΔE_{orb}[a]	-295.2 (40.7 %)	-156.1 (27.5 %)	-140.3 (26.7 %)	-116.0 (23.9 %)	-113.1 (24.0 %)
ΔE_{disp}[a]	-10.52 (1.4 %)	-8.8 (1.5 %)	-9.4 (1.8 %)	-9.0 (1.9 %)	-9.8 (2.1 %)
$\Delta E_{\rho 1}$(a' → p_x)[b]	-61.7 (20.9 %)	-20.8 (13.2 %)	-	-	-
$\Delta E_{\rho 2}$ (a'' → p_y)[b]	-61.4 (20.8 %)	-20.7 (13.2 %)	-9.3 (6.6 %)	-	-
$\Delta E_{\rho 3}$ (a' → p_z)[b]	-46.6 (15.7 %)	-17.0 (10.8 %)	-6.2 (4.4 %)	-4.7 (3.3 %)	-4.0 (3.5 %)
$\Delta E_{\rho 4}$(a' → s)[b]	-45.0 (15.2 %)	-29.4 (18.7 %)	-10.0 (7.1 %)	-8.0 (5.6 %)	-7.9 (7.0 %)
$\Delta E_{\rho 5}$ (a' → d_{xz})[b]	-10.6 (3.6 %)	-12.6 (8.0 %)	-33.1 (23.5 %)	-27.3 (19.1 %)	-25.7 (22.7 %)
$\Delta E_{\rho 6}$ (a' → d_{yz})[b]	-10.5 (3.6 %)	-12.5 (8.0 %)	-30.6 (21.7 %)	-25.6 (17.9 %)	-22.6 (20.0 %)
$\Delta E_{\rho 7}$ (a' → d_{xy})[b]	-	-	-	-7.0 (4.9 %)	-9.3 (8.2 %)
$\Delta E_{\rho 8}$ (a' → d_{x2y2})[b]	-	-	-19.7 (14.0 %)	-	-
$\Delta E_{\rho 9}$ (a' → d_{z2})[b]	-	-	-	-16.6 (11.6 %)	-18.9 (16.6 %)
ΔE_{orb}(rest)[b]	-59.7 (20.2 %)	-43.8 (27.9 %)	-31.8 (22.6 %)	-54.0 (37.7 %)	-25.0 (22.0 %)
ΔE_{prep}	0.6	0.3	0.5	0.5	0.4
$-D_e$	-705.4	-569.4	-502.5	-465.5	-440.3
	$[Be(C_5Cl_5)_2]$	$[Mg(C_5Cl_5)_2]$	$[Ca(C_5Cl_5)_2]$	$[Sr(C_5Cl_5)_2]$	$[Ba(C_5Cl_5)_2]$
Fragments	$Be^{2+}(s^0)$;2$(C_5Cl_5)^-$(a'^{32} a''^{20})	$Mg^{2+}(s^0)$; 2$(C_5Cl_5)^-$(a'^{32} a''^{20})	$Ca^{2+}(s^0)$; 2$(C_5Cl_5)^-$(a'^{32} a''^{20})	$Sr^{2+}(s^0)$; 2$(C_5Cl_5)^-$(a'^{32} a''^{20})	$Ba^{2+}(s^0)$; 2$(C_5Cl_5)^-$(a'^{32} a''^{20})
ΔE_{int}	-629.9	-489.1	-433.1	-400.2	-381.4
ΔE_{Pauli}	80.7	69.8	84.0	79.6	82.8
ΔE_{elstat}[a]	-362.5 (51.0 %)	-355.1 (63.5 %)	-337.8 (65.3 %)	-326.5 (68.0 %)	-318.1 (68.5 %)
ΔE_{orb}[a]	-322.4 (45.4 %)	-183.3 (32.8 %)	-158.7 (30.7 %)	-133.3 (27.8 %)	-125.5 (27.0 %)
ΔE_{disp}[a]	-25.7 (3.6 %)	-20.6 (3.7 %)	-20.6 (4.0 %)	-20.0 (4.2 %)	-20.7 (4.4 %)
$\Delta E_{\rho 1}$(a' → p_x)[b]	-57.8 (17.9 %)	-19.4 (10.6 %)	-	-	-
$\Delta E_{\rho 2}$ (a'' → p_y)[b]	-57.6 (17.9 %)	-19.4 (10.5 %)	-	-	-

$\Delta E_{\rho3}$ (a’ $\rightarrow$ p_z)[b]	-47.5 (14.7 %)	-17.3 (9.4 %)	-6.1 (3.8 %)	-	-
$\Delta E_{\rho4}$(a’ $\rightarrow$s)[b]	-45.1 (14.0 %)	-27.7 (15.1 %)	-9.1 (5.7 %)	-8.2 (6.1 %)	-
$\Delta E_{\rho5}$ (a’ $\rightarrow$ d_{xz})[b]	-	-13.6 (7.4 %)	-31.0 (19.5 %)	-24.3 (18.2 %)	-22.2 (17.7 %)
$\Delta E_{\rho6}$ (a’ $\rightarrow$ d_{yz})[b]	-	-13.5 (7.4 %)	-28.3 (17.8 %)	-21.7 (16.2 %)	-19.2 (15.3 %)
$\Delta \mathrm{E}_{\rho7}$ (a’ $\rightarrow$ d_{xy})[b]	-	-	-8.5 (5.4 %)	-7.6 (5.7 %)	-8.8 (7.0 %)
$\Delta \mathrm{E}_{\rho8}$ (a’ $\rightarrow$ d_{x2y2})[b]	-	-	-	-	-18.6 (14.8 %)
$\Delta \mathrm{E}_{\rho9}$ (a’ $\rightarrow$ d_{z2})[b]	-	-	-19.8 (12.5 %)	-18.0 (13.5 %)	-7.2 (5.7 %)
ΔE_{orb}(rest)[b]	-114.4 (35.5 %)	-72.6 (39.6 %)	-56.1 (35.3 %)	-53.8 (40.3 %)	-49.5 (39.4 %)
ΔE_{prep}	1.7	1.8	1.6	1.6	1.3
$-D_e$	-723.4	-579.4	-509.4	-471.0	-448.0
	$[Be(C_5Br_5)_2]$	$[Mg(C_5Br_5)_2]$	$[Ca(C_5Br_5)_2]$	$[Sr(C_5Br_5)_2]$	$[Ba(C_5Br_5)_2]$
Fragments	Be^{2+} (s^0);2$(C_5Br_5)^-$(a’^{32} a’’^{20})	Mg^{2+}(s^0); 2$(C_5Br_5)^-$(a’^{32} a’’^{20})	Ca^{2+}(s^0); 2$(C_5Br_5)^-$(a’^{32} a’’^{20})	Sr^{2+}(s^0); 2$(C_5Br_5)^-$(a’^{32} a’’^{20})	Ba^{2+}(s^0); 2$(C_5Br_5)^-$(a’^{32} a’’^{20})
ΔE_{int}	-635.4	-494.2	-440.7	-407.7	-388.2
ΔE_{Pauli}	81.4	71.1	82.0	77.5	79.5
ΔE_{elstat}[a]	-350.6 (48.9 %)	-340.6 (60.2 %)	-328.1 (62.8 %)	-318.0 (65.5 %)	-310.3 (66.3 %)
ΔE_{orb}[a]	-333.2 (46.5 %)	-196.3 (34.7 %)	-168.3 (32.2 %)	-142.1 (29.3 %)	-132.4 (28.3 %)
ΔE_{disp}[a]	-33.1 (4.6 %)	-28.3 (5.0 %)	-26.2 (5.2 %)	-25.1 (5.2 %)	-25.0 (5.3 %)
$\Delta E_{\rho1}$(a’ $\rightarrow$ p_x)[b]	-57.9 (17.4 %)1	-20.2 (10.2 %)	-	-	
$\Delta E_{\rho2}$ (a’’$\rightarrow$ p_y)[b]	-57.8 (17.4 %)2	-20.1 (10.2 %)	-	-	
$\Delta E_{\rho3}$ (a’$\rightarrow$ p_z)[b]	-47.2 (14.2 %)4	-17.4 (8.8 %)	-	-	
$\Delta E_{\rho4}$(a’ $\rightarrow$s)[b]	-46.3 (13.9 %)3	-27.8 (14.1 %)	-6.1 (3.6 %)	-5.2 (3.6 %)	-4.5 (3.4 %)
$\Delta E_{\rho5}$ (a’$\rightarrow$ d_{xz})[b]	-	-14.1 (7.2 %)	-30.7 (18.2 %)	-24.0 (16.8 %)	-21.8 (16.4 %)
$\Delta E_{\rho6}$ (a’$\rightarrow$ d_{yz})[b]	-	-14.0 (7.1 %)	-28.5 (16.9 %)	-21.6 (15.2 %)	-19.4 (14.7 %)
$\Delta \mathrm{E}_{\rho7}$ (a’$\rightarrow$ d_{xy})[b]	-	-	-8.5 (5.0 %)	-7.7 (5.4 %)	-8.5 (6.4 %)
$\Delta \mathrm{E}_{\rho8}$ (a’$\rightarrow$ d_{x2y2})[b]	-	-	-19.3 (11.4 %)	-8.3 (5.8 %)	-17.8 (13.4 %)
$\Delta \mathrm{E}_{\rho9}$ (a’$\rightarrow$ d_{z2})[b]	-	-	-9.0 (5.4 %)	-17.7 (12.4 %)	-7.2 (5.4 %)
ΔE_{orb}(rest)[b]	-123.9 (37.2 %)	-83.2 (42.3 %)7	-66.5 (39.4 %)	-57.9 (40.7 %)	-53.3 (40.2 %)
ΔE_{prep}	1.7	1.8			
$-D_e$	-723.4	-579.4			
	$[Be(C_5I_5)_2]$	$[Mg(C_5I_5)_2]$	$[Ca(C_5I_5)_2]$	$[Sr(C_5I_5)_2]$	$[Ba(C_5I_5)_2]$
Fragments	Be^{2+} (s^0); 2$(C_5I_5)^-$(a’^{32} a’’^{20})	Mg^{2+}(s^0); 2$(C_5I_5)^-$(a’^{32} a’’^{20})	Ca^{2+}(s^0); 2$(C_5I_5)^-$(a’^{32} a’’^{20})	Sr^{2+}(s^0); 2$(C_5I_5)^-$(a’^{32} a’’^{20})	Ba^{2+}(s^0); 2$(C_5I_5)^-$(a’^{32} a’’^{20})
ΔE_{int}	-640.67	-505.2	-448.3	-414.5	-395.5
ΔE_{Pauli}	91.9	79.8	87.7	81.6	82.1
ΔE_{elstat}[a]	-344.28 (47.0 %)	-334.3 (57.1 %)	-318.7 (59.5 %)	-309.0 (62.3 %)	-303.3 (63.5 %)
ΔE_{orb}[a]	-343.2 (46.8 %)	-209.6 (35.8 %)	-180.3 (33.6 %)	-152.9 (30.8 %)	-141.4 (29.6 %)

ΔE_{disp}[a]	-45.1 (6.1 %)	-41.2 (7.0 %)	-36.9 (6.9 %)	-34.1 (6.9 %)	-32.9 (6.9 %)
$\Delta E_{\rho 1}$(a' → p_x)[b]	-56.8 (16.5 %)	-20.2 (9.6 %)	-	-	-
$\Delta E_{\rho 2}$ (a'' → p_y)[b]	-56.6 (16.5 %)	-20.1 (9.6 %)	-	-	-
$\Delta E_{\rho 3}$ (a' → p_z)[b]	-46.8 (13.6 %)	-17.5 (8.3 %)	-	-	-
$\Delta E_{\rho 4}$(a' → s)[b]	-46.6 (13.6 %)	-27.6 (13.1 %)	-7.8 (4.3 %)	-7.3 (4.8 %)	-5.9 (4.1 %)
$\Delta E_{\rho 5}$ (a' → d_{xz})[b]	-	-14.4 (6.8 %)	-30.5 (16.9 %)	-23.6 (15.4 %)	-21.4 (15.1 %)
$\Delta E_{\rho 6}$ (a' → d_{yz})[b]	-	-14.3 (6.8 %)	-29.6 (16.4 %)	-22.1 (14.4 %)	-20.0 (14.1 %)
$\Delta \mathrm{E}_{\rho 7}$ (a' → d_{xy})[b]	-		-8.2 (4.5 %)	-7.9 (5.2 %)	-8.2 (5.8 %)
$\Delta \mathrm{E}_{\rho 8}$ (a' → d_{x2y2})[b]	-		-17.7 (9.8 %)	-17.0 (11.1 %)	-16.9 (11.9 %)
$\Delta \mathrm{E}_{\rho 9}$ (a' → d_{z2})[b]	-		-8.4 (4.7 %)	-8.0 (5.2 %)	-7.6 (5.4 %)
ΔE_{orb}(rest)[b]	-136.1 (39.7 %)	-96.0 (45.6 %)	-78.2 (43.3 %)	-67.2 (43.9 %)	-61.7 (43.5 %)
ΔE_{prep}	1.7	1.7	1.7	1.7	1.7
$-D_e$	-723.4	-723.4	-723.4	-723.4	-723.4

[a] The value in parenthesis gives the percentage contribution to the total attractive interactions $\Delta E_{elstat} + \Delta E_{orb} + \Delta E_{disp}$. [b] The value in parenthesis gives the percentage contribution to the total orbital interactions ΔE_{orb}.

[$Be(C_5F_5)_2$]

**Deformation Densities**	**$(C_5F_5)_2^{2-}$ orbitals**	**Be^{2+} orbitals**

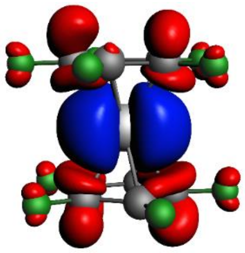
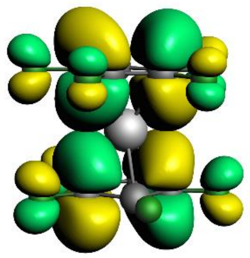
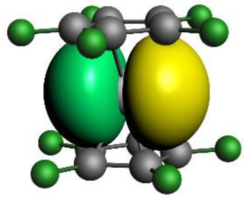

ΔE_1=-61.7; $|\nu_1|$=0.49 HOMO (ε=0.61 eV) ν_1= -0.24 LUMO+1 (ε=-17.36 eV) ν_1=+0.38

ΔE_2=-61.4; $|\nu_2|$=0.49 HOMO (ε=0.61 eV) ν_2= -0.24 LUMO+1 (ε=-17.36 eV) ν_2=+0.38

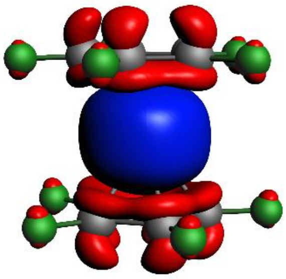
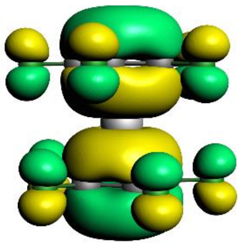
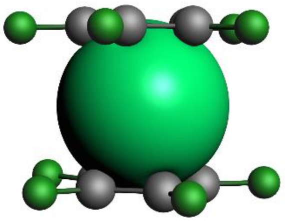

ΔE_3=-45.0; $|\nu_3|$=0.36 HOMO-1 (ε=-2.75 eV) ν_3= -0.3 LUMO (ε=-21.1 eV) ν_3=+0.46

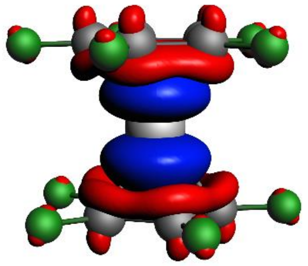
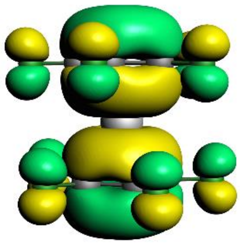
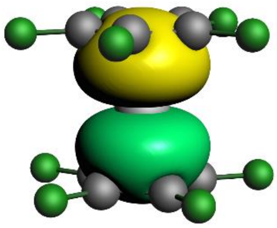

ΔE_4= -46.6; $|\nu_4|$= 0.37 HOMO-1 (ε=-2.75 eV) ν_4= -0.26 LUMO+1 (ε=-17.36 eV) ν_4=+0.37

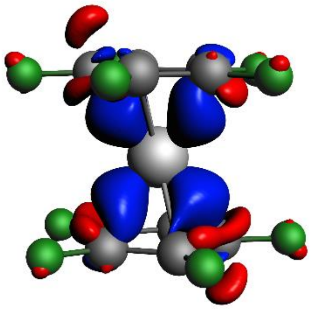
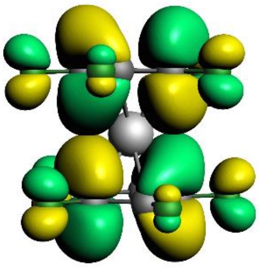
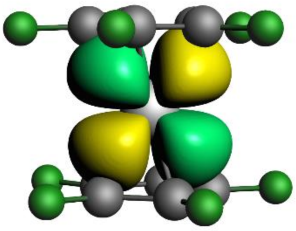

ΔE_5= -10.6; $|\nu_5|$= 0.14 HOMO (ε=0.61 eV) ν_5= -0.02 LUMO+5 (ε=10.4 eV) ν_5=+0.036

ΔE_6= -10.6; $|\nu_6|$= 0.14 | HOMO (ε=0.61 eV) ν_6= -0.02 | LUMO+5 (ε=10.4 eV) ν_6=+0.036

[Mg(C$_5$F$_5$)$_2$]

**Deformation Densities**	**$(C_5F_5)_2^{2-}$ orbitals**	**Mg^{2+} orbitals**

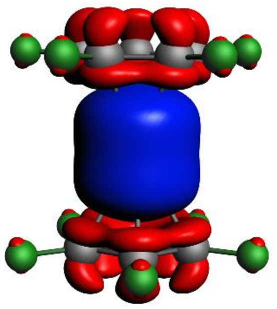
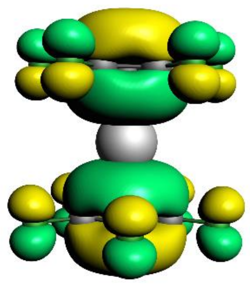
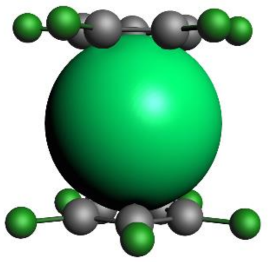

ΔE_1=-29.4; $|\nu_1|$=0.36 | HOMO-1 (ε=-2.79 eV) ν_1= -0.24 | LUMO (ε=-17.84 eV) ν_1=+0.31

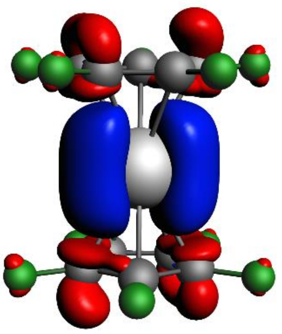
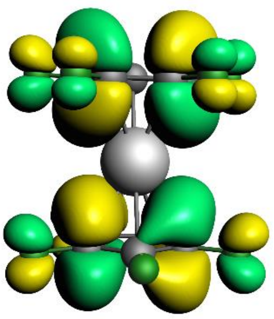
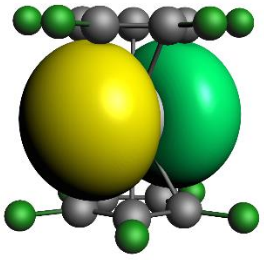

ΔE_2= -20.8; $|\nu_2|$=0.30 | HOMO (ε=0.55 eV) ν_2= -0.12 | LUMO+1 (ε=-12.63 eV) ν_2=+0.24

ΔE_3=-20.7; $|\nu_3|$=0.30 | HOMO (ε=0.55 eV) ν_3= -0.12 | LUMO+1 (ε=-12.63 eV) ν_3=+0.24

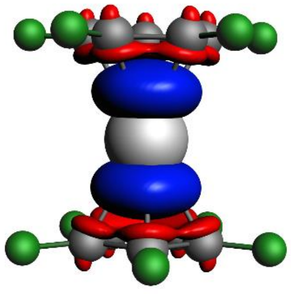
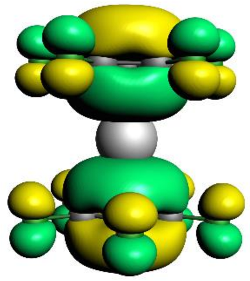
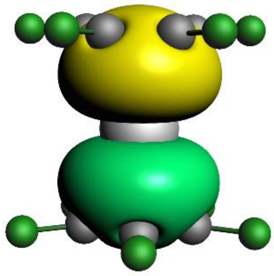

ΔE_4=-17.0; $|\nu_4|$=0.22 | HOMO-1 (ε=-2.79 eV) ν_4= -0.16 | LUMO+1 (ε=-12.63 eV) ν_4=+0.24

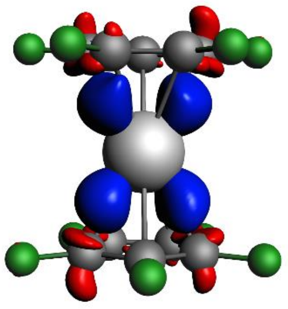
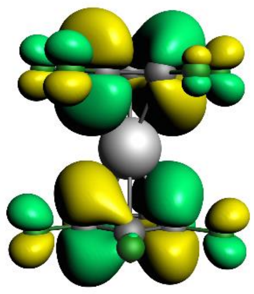
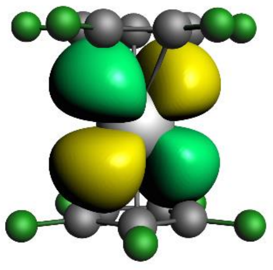

ΔE_5= -12.5; $|\nu_5|$= 0.18 HOMO (ε=0.55 eV) ν_5= -0.06 LUMO+3 (ε=-3.04 eV) ν_5=+0.08

ΔE_6= -12.5; $|\nu_6|$= 0.18 HOMO (ε=0.55 eV) ν_6= -0.06 LUMO+3 (ε=-3.04 eV) ν_6=+0.08

[Ca(C_5F_5)$_2$]

**Deformation Densities**	**$(C_5F_5)_2^{2-}$ orbitals**	**Ca^{2+} orbitals**
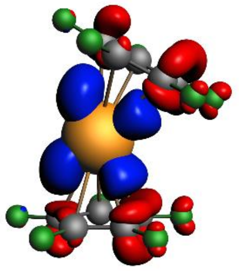	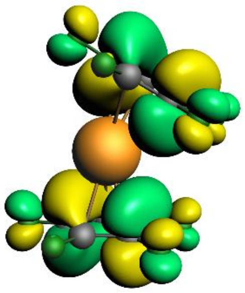	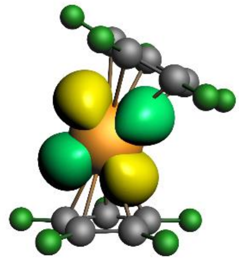
ΔE_1= -33.1; $|\nu_1|$=0.51	HOMO (ε=0.50 eV) ν_1= -0.22	LUMO (ε=-15.91 eV) ν_1= +0.24
ΔE_2=-30.6; $|\nu_2|$=0.49	HOMO (ε=0.50 eV) ν_2= -0.20	LUMO (ε=-15.91 eV) ν_2= +0.22
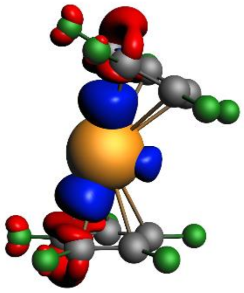	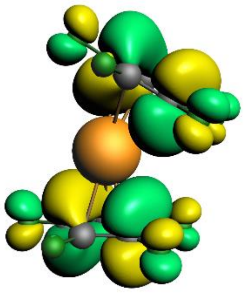	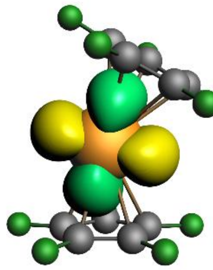
ΔE_3= -19.7; $|\nu_3|$=0.35	HOMO (ε=0.50 eV) ν_3= -0.1	LUMO (ε=-15.91 eV) ν_3= +0.11
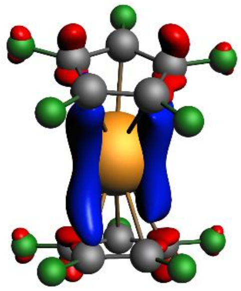	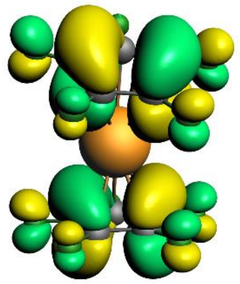	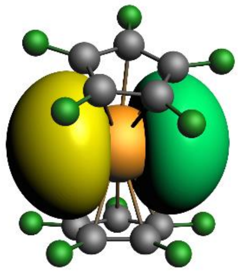
ΔE_4= -9.3; $|\nu_4|$=0.22	HOMO (ε=0.50 eV) ν_4= -0.06	LUMO+2 (ε=-10.53 eV) ν_4= +0.03

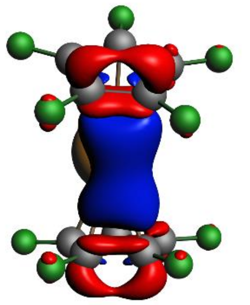
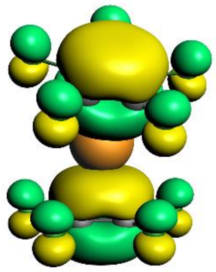
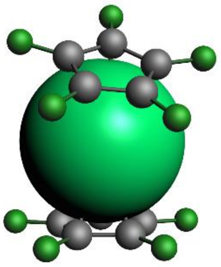

ΔE_5= -10.0; $|\nu_5|$=0.19

HOMO-1 (ε=-2.86 eV) ν_5= -0.08

LUMO+1 (ε=-14.24 eV) ν_5= +0.12

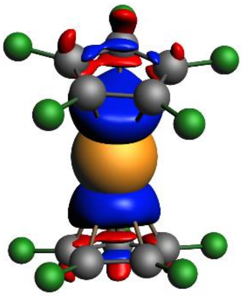
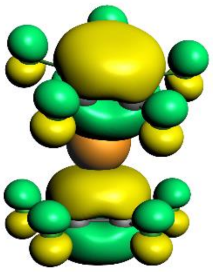
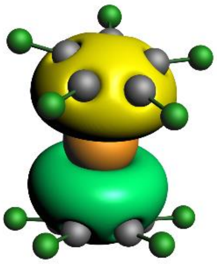

ΔE_6= -6.2; $|\nu_6|$=0.12

HOMO-1 (ε=-2.86 eV) ν_6= -0.06

LUMO+2 (ε=-10.53 eV) ν_6= +0.06

[Sr(C_5F_5)$_2$]

Deformation Densities | **$(C_5F_5)_2^{2-}$ orbitals** | **Sr^{2+} orbitals**

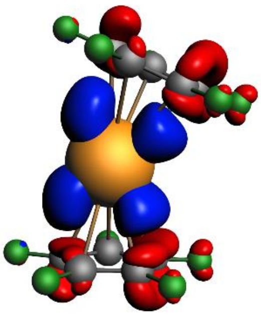
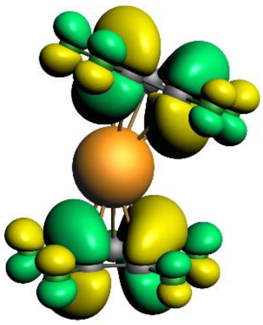
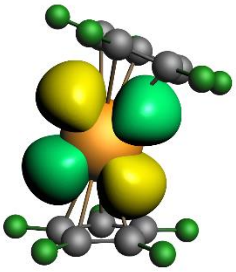

ΔE_1= -27.3; $|\nu_1|$=0.47

ΔE_2=-25.6; $|\nu_2|$=0.45

HOMO (ε=0.46 eV) ν_1= -0.24

HOMO (ε=0.46 eV) ν_2= -0.20

LUMO (ε=-13.22 eV) ν_1= +0.24

LUMO (ε=-13.22 eV) ν_2= +0.22

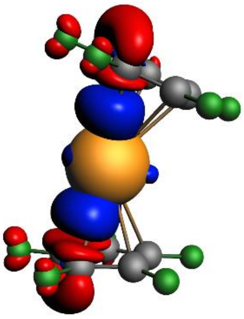
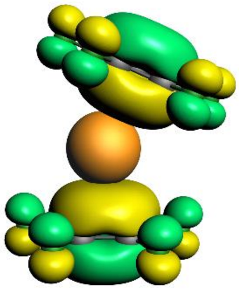
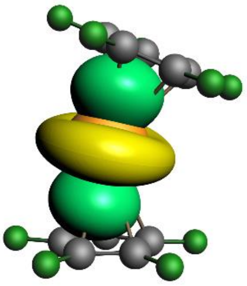

ΔE_3=-16.6; $|\nu_3|$=0.32

HOMO-1 (ε=-2.90 eV) ν_3= -0.08

LUMO (ε=-13.22 eV) ν_3= +0.11

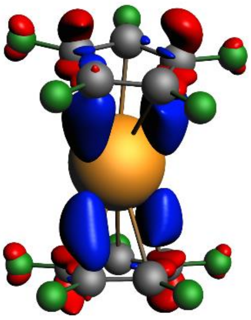

ΔE_4=-7.0; $|\nu_4|$=0.18

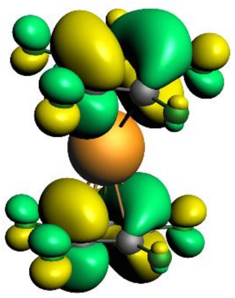

HOMO (ε=0.46 eV) ν_4= -0.20

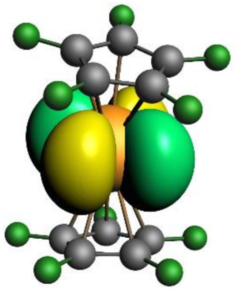

LUMO (ε=-13.22 eV) ν_4= +0.02

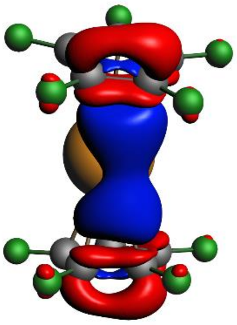

ΔE_5=-8.0; $|\nu_5|$=0.18

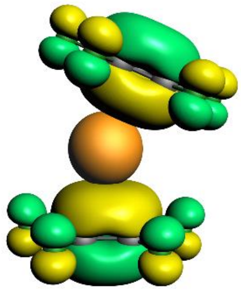

HOMO-1 (ε=-2.90 eV) ν_5= -0.06

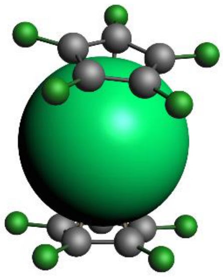

LUMO (ε=-13.22 eV) ν_5= +0.07

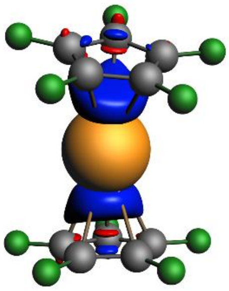

ΔE_6=-4.7; $|\nu_6|$=0.11

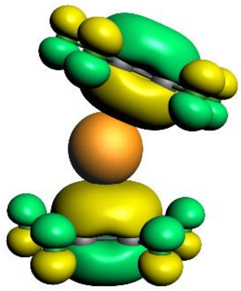

HOMO-1 (ε=-2.90 eV) ν_6= -0.02

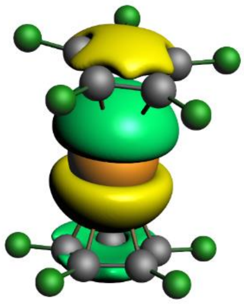

LUMO+4 (ε=0.53 eV) ν_6= +0.02

[Ba(C_5F_5)$_2$]

Deformation Densities | **(C_5F_5)$_2^{2-}$ orbitals** | **Ba^{2+} orbitals**

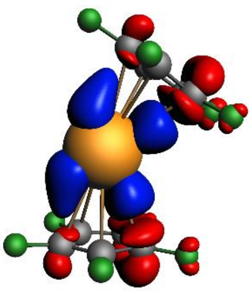

ΔE_1= -25.7; $|\nu_1|$=0.48

ΔE_2=-22.6; $|\nu_2|$=0.43

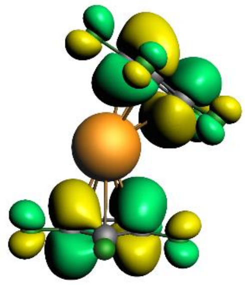

HOMO (ε=0.45 eV) ν_1= -0.2

HOMO (ε=0.45 eV) ν_2= -0.16

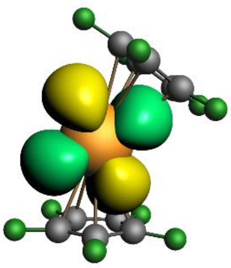

LUMO (ε=-12.82 eV) ν_1= + 0.21

LUMO (ε=-12.82 eV) ν_2= + 0.19

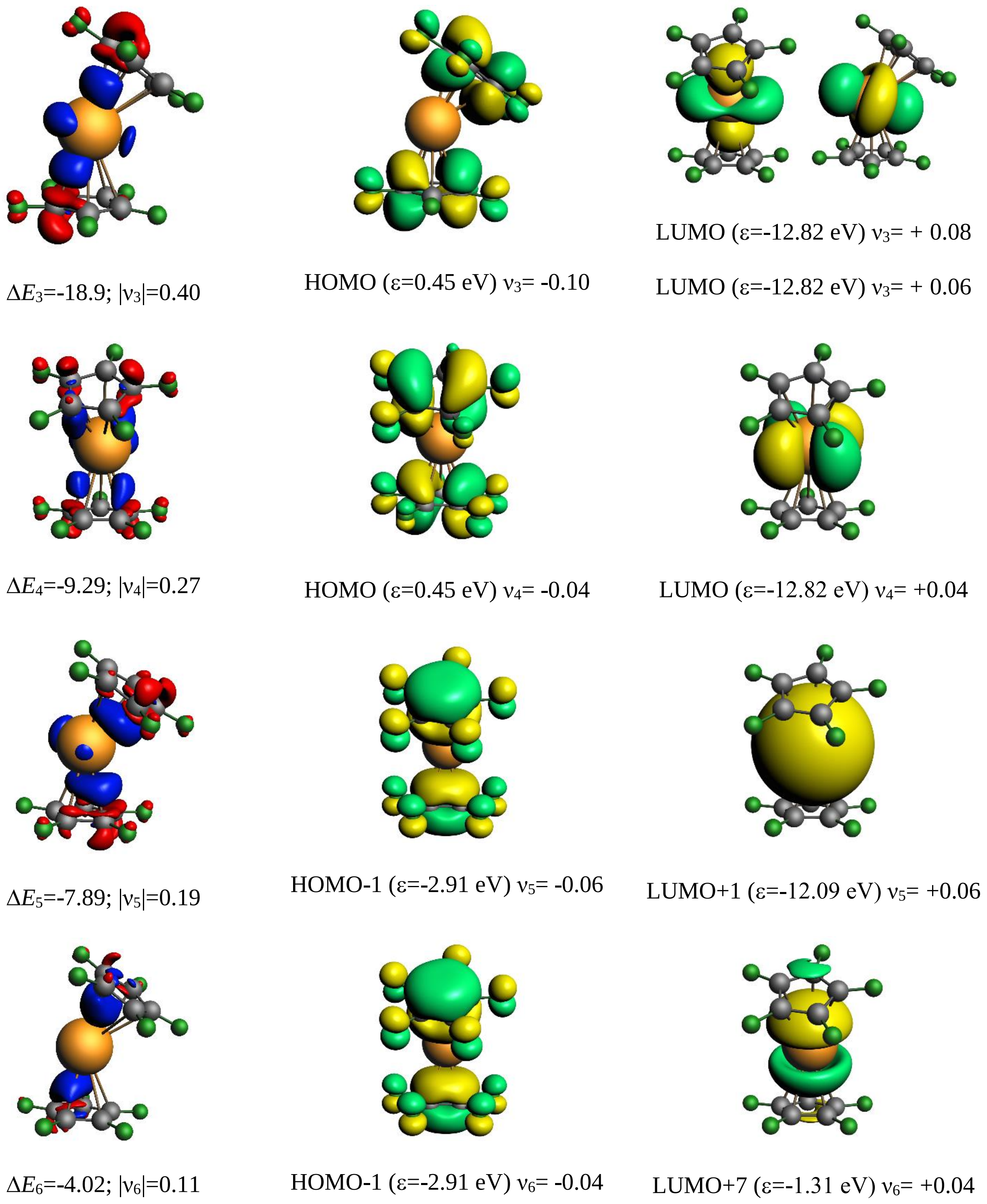

Figure S3. Plot of deformation densities (isovalue = 0.003) of the pairwise orbital interaction and shape of the most important occupied and vacant orbitals (isovalue = 0.03) in $[Ae(C_5F_5)_2]$ (Ae=Be-Ba) with the orbital interaction energies ΔE_{orb} (in kcal/mol) and their eigenvalues ν (in e). The direction of the charge flow is red→blue. The eigenvalues ν indicate the amount of donated (negative numbers) and accepted charge (positive numbers). The occupied orbitals are shown in yellow and blue for the different phases, while the unoccupied orbitals are in cyan and orange.

$[Be(C_5Cl_5)_2]$

**Deformation Densities**	**$(C_5Cl_5)_2^{2-}$ orbitals**	**Be^{2+} orbitals**
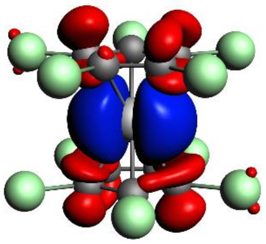	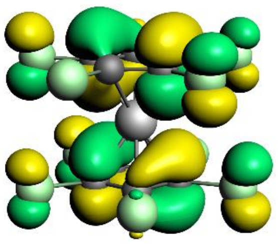	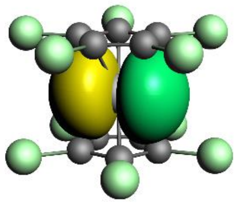
ΔE_1= -57.8; $\lvert\nu_1\rvert$= 0.47	HOMO (ε=-0.73 eV) ν_1= -0.28	LUMO+1 (ε=-17.36 eV) ν_1=+0.43
ΔE_2= -57.6; $\lvert\nu_2\rvert$= 0.47	HOMO (ε=-0.73 eV) ν_2= -0.28	LUMO+1 (ε=-17.36 eV) ν_2=+0.43
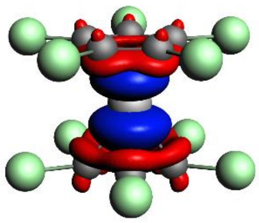	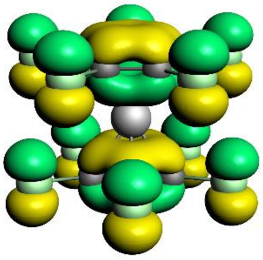	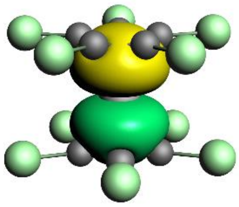
ΔE_3= -47.5; $\lvert\nu_3\rvert$= 0.37	HOMO-1 (ε=-3.20 eV) ν_3= -0.15	LUMO+1 (ε=-17.36 eV) ν_3=+0.33
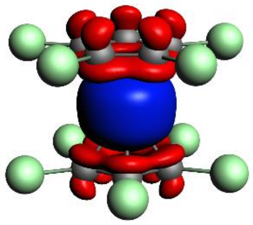	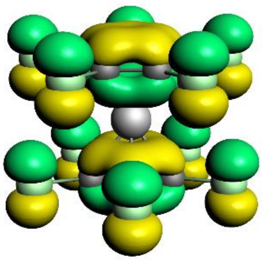	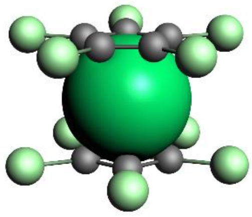
ΔE_4= -45.2; $\lvert\nu_4\rvert$= 0.37	HOMO-1 (ε=-3.20 eV) ν_4= -0.18	LUMO (ε=-21.09 eV) ν_4=+0.41

$[Mg(C_5Cl_5)_2]$

**Deformation Densities**	**$(C_5Cl_5)_2^{2-}$ orbitals**	**Mg^{2+} orbitals**

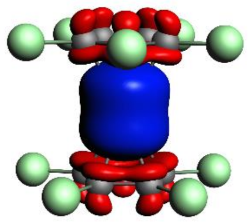
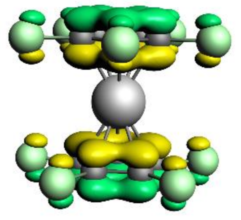
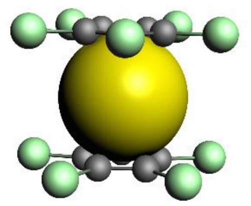

ΔE_1= -27.6; $|\nu_1|$= 0.33   HOMO-6 (ε=-6.68 eV) ν_1= -0.18   LUMO (ε=-17.85 eV) ν_1=+0.34

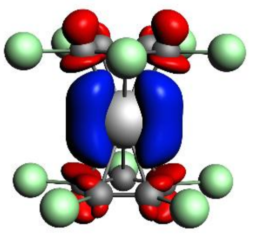
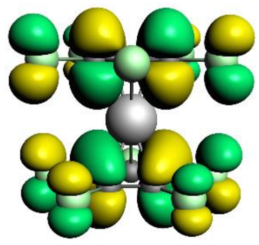
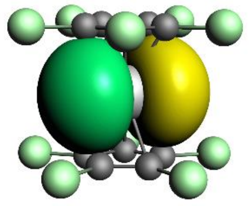

ΔE_2= -19.4; $|\nu_2|$= 0.28   HOMO (ε=-0.75 eV) ν_2= -0.16   LUMO+1 (ε=-12.64 eV) ν_2=+ 0.18

ΔE_3= -19.4; $|\nu_3|$= 0.28   HOMO (ε=-0.75 eV) ν_3= -0.16   LUMO+1 (ε=-12.64 eV) ν_3=+ 0.18

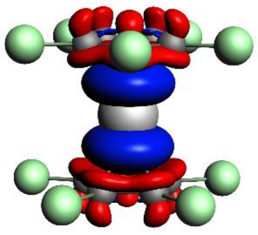
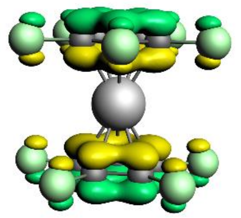
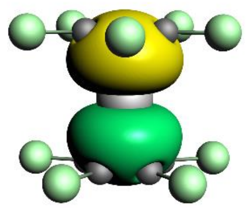

ΔE_4= -17.3; $|\nu_4|$= 0.22   HOMO-6 (ε=-6.68 eV) ν_4= -0.08   LUMO+1 (ε=-12.64 eV) ν_4=+ 0.19

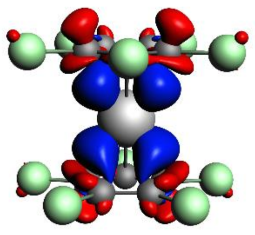
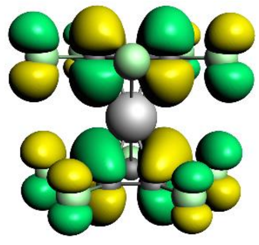
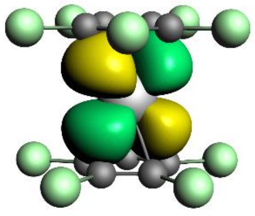

ΔE_5= -13.6; $|\nu_5|$= 0.18   HOMO (ε=-0.75 eV) ν_5= -0.06   LUMO+3 (ε=-3.04 eV) ν_5=+ 0.06

ΔE_6= -13.6; $|\nu_6|$= 0.18   HOMO (ε=-0.75 eV) ν_6= -0.06   LUMO+3 (ε=-3.04 eV) ν_6=+ 0.06

[$Ca(C_5Cl_5)_2$]

Deformation Densities   **$(C_5Cl_5)_2^{2-}$ orbitals**   **Ca^{2+} orbitals**

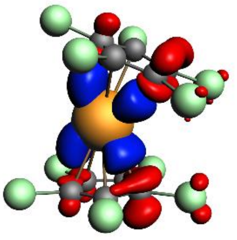
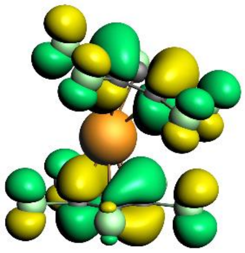
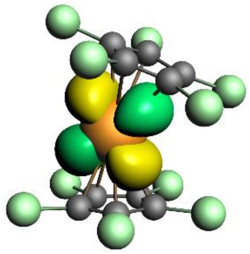

ΔE_1= -31.0; $|\nu_1|$= 0.48 HOMO (ε=-0.79 eV) ν_1= -0.19 LUMO (ε=-15.91 eV) ν_1=+ 0.21

ΔE_2= -28.2; $|\nu_2|$= 0.46 HOMO (ε=-0.79 eV) ν_2= -0.17 LUMO (ε=-15.91 eV) ν_2=+ 0.19

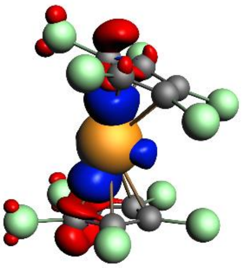
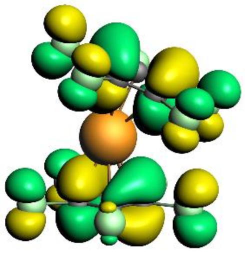
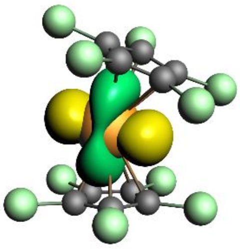

ΔE_3= -19.8; $|\nu_3|$= 0.35 HOMO (ε=-0.79 eV) ν_3= -0.08 LUMO (ε=-15.91 eV) ν_3=+ 0.10

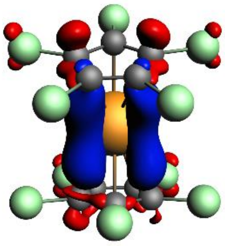
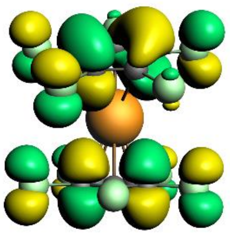
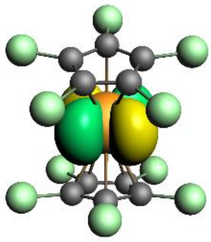

ΔE_4= -8.5; $|\nu_4|$= 0.21 HOMO (ε=-0.79 eV) ν_4= -0.04 LUMO (ε=-15.91 eV) ν_4=+ 0.03

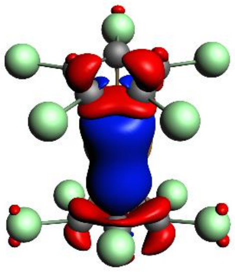
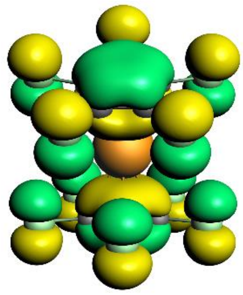
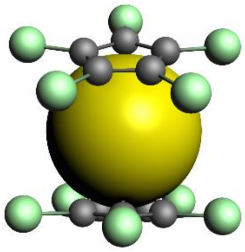

ΔE_5= -9.1; $|\nu_5|$= 0.19 HOMO-1 (ε=-3.25 eV) ν_5= -0.02 LUMO+1 (ε=-14.24 eV) ν_5=+ 0.07

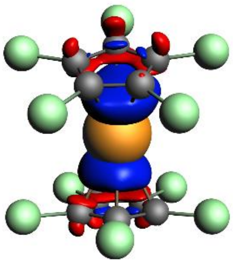
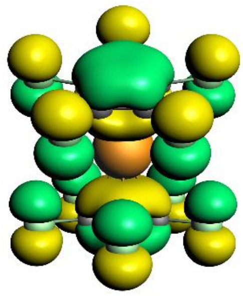
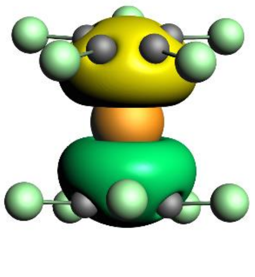

LUMO+2 (ε=-10.53 eV) ν_6=+ 0.06

ΔE_6= -6.1; $|\nu_6|$= 0.12 HOMO-1 (ε=-3.25 eV) ν_6= -0.02

[$Sr(C_5Cl_5)_2$]

**Deformation Densities**	**$(C_5Cl_5)_2^{2-}$ orbitals**	**Sr^{2+} orbitals**
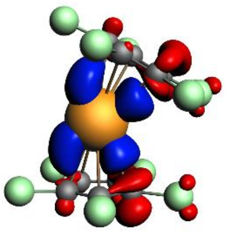	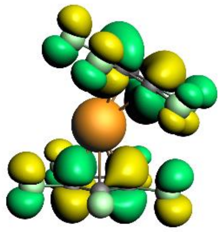	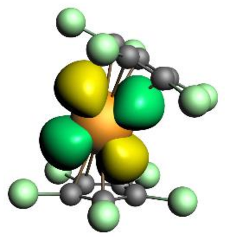
ΔE_1= -24.3; $|\nu_1|$= 0.42	HOMO (ε=-0.80 eV) ν_1= -0.16	LUMO (ε=-13.22 eV) ν_1=+ 0.20
ΔE_2= -21.7; $|\nu_2|$= 0.39	HOMO (ε=-0.80 eV) ν_2= -0.12	LUMO (ε=-13.22 eV) ν_2=+ 0.17
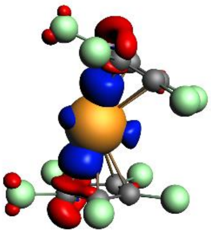	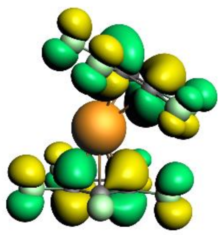	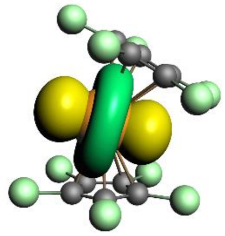
ΔE_3= -18.0; $|\nu_3|$= 0.35	HOMO (ε=-0.80 eV) ν_3= -0.08	LUMO (ε=-13.22 eV) ν_3=+ 0.08
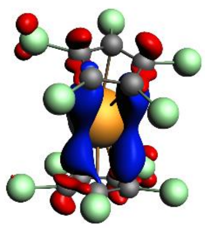	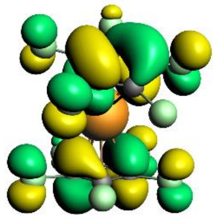	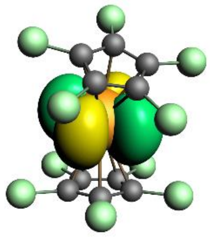
ΔE_4= -7.6; $|\nu_4|$= 0.21	HOMO (ε=-0.80 eV) ν_4= -0.04	LUMO (ε=-13.22 eV) ν_4=+ 0.04
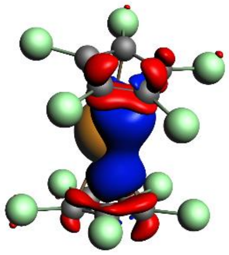	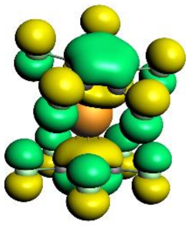	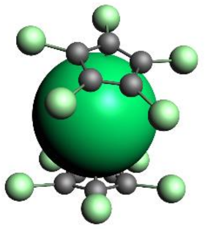
ΔE_5= -8.2; $|\nu_5|$= 0.18	HOMO-1 (ε=-3.26 eV) ν_5= -0.02	LUMO (ε=-13.22 eV) ν_5=+ 0.06

[$Ba(C_5Cl_5)_2$]

**Deformation Densities**	**$(C_5Cl_5)_2^{2-}$ orbitals**	**Ba^{2+} orbitals**

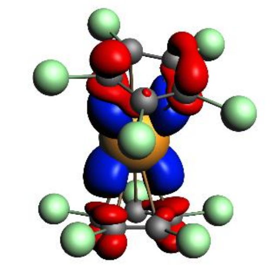

ΔE_1= -22.1; $|\nu_1|$= 0.42

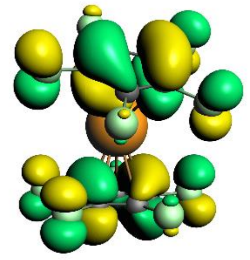

HOMO (ε=-0.81 eV) ν_1= -0.16

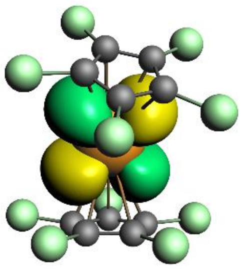

LUMO (ε=-12.82 eV) ν_1= +0.19

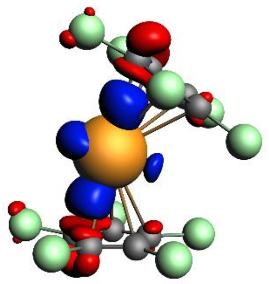

ΔE_2= -18.6; $|\nu_2|$= 0.39

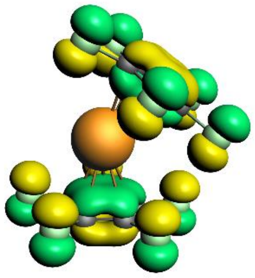

HOMO-1 (ε=-3.27 eV) ν_2= -0.04

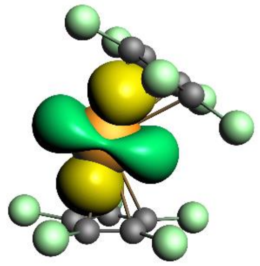

LUMO (ε=-12.82 eV) ν_2= +0.17

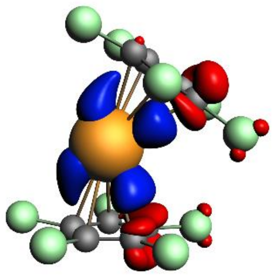

ΔE_3= -19.2; $|\nu_3|$= 0.37

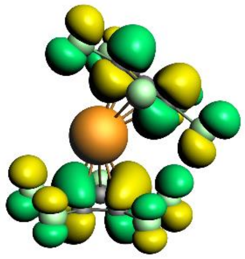

HOMO (ε=-0.81 eV) ν_3= -0.10

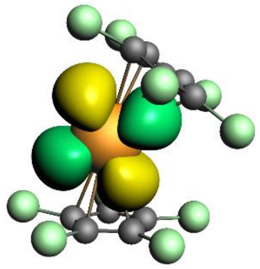

LUMO (ε=-12.82 eV) ν_3= +0.16

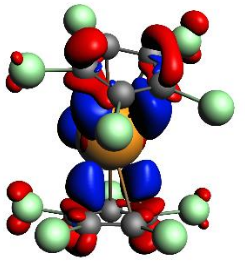

ΔE_4= -8.8; $|\nu_4|$= 0.26

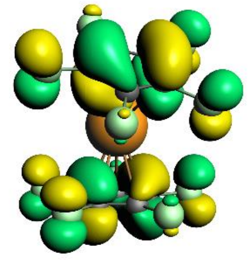

HOMO (ε=-0.81 eV) ν_4= -0.04

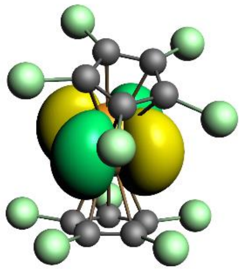

LUMO (ε=-12.82 eV) ν_4= +0.05

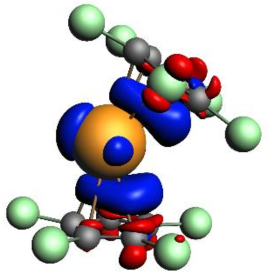

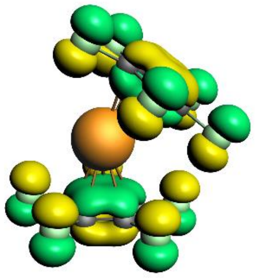

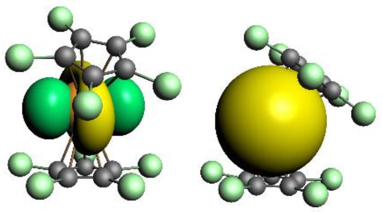

LUMO (ε=-12.82 eV) ν_5= +0.03

ΔE_5= -7.2; $|\nu_5|$= 0.19 HOMO-1 (ε=-3.27 eV) ν_5= -0.02 LUMO+1 (ε=-12.09 eV) ν_5= +0.03

Figure S4. Plot of deformation densities (isovalue = 0.003) of the pairwise orbital interaction and shape of the most important occupied and vacant orbitals (isovalue = 0.03) in [Ae(C_5Cl_5)$_2$] (Ae=Be-Ba) with the orbital interaction energies ΔE_{orb} (in kcal/mol) and their eigenvalues ν (in e). The direction of the charge flow is red→blue. The eigenvalues ν indicate the amount of donated (negative numbers) and accepted charge (positive numbers). The occupied orbitals are shown in yellow and blue for the different phases, while the unoccupied orbitals are in cyan and orange.

[Be(C_5Br_5)$_2$]

**Deformation Densities**	**(C_5Br_5)$_2^{2-}$ orbitals**	**Be^{2+} orbitals**

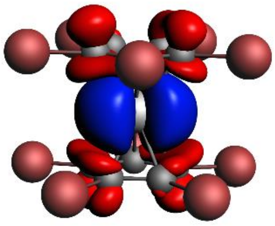

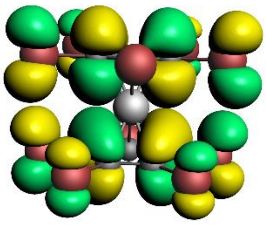

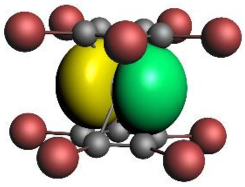

ΔE_1= -57.8; $|\nu_1|$= 0.47 HOMO (ε=-0.99 eV) ν_1= -0.23 LUMO+1 (ε=-17.36 eV) ν_1=+0.41

ΔE_2= -57.8; $|\nu_2|$= 0.47 HOMO (ε=-0.99 eV) ν_2= -0.23 LUMO+1 (ε=-17.36 eV) ν_2=+0.41

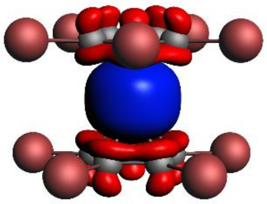

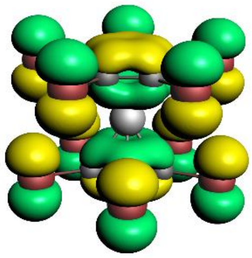

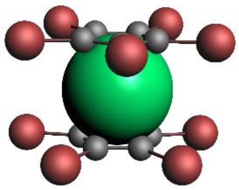

ΔE_3= -46.2; $|\nu_3|$= 0.37 HOMO-1 (ε=-3.14 eV) ν_3= -0.14 LUMO (ε=-21.09 eV) ν_3=+0.41

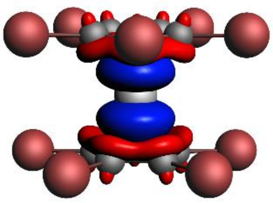

ΔE_4= -47.2; $|\nu_4|$= 0.37

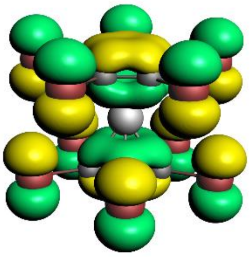

HOMO-1 (ε=-3.14 eV) ν_4= -0.12

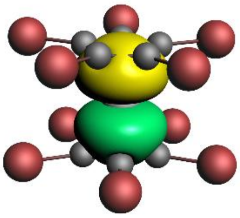

LUMO+1 (ε=-17.36 eV) ν_4=+0.33

[$Mg(C_5Br_5)_2$]

**Deformation Densities**	**$(C_5Br_5)_2^{2-}$ orbitals**	**Mg^{2+} orbitals**

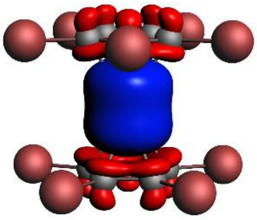

ΔE_1= -27.6; $|\nu_1|$= 0.33

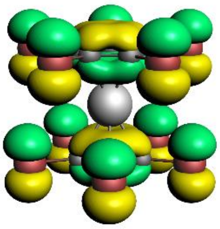

HOMO-1 (ε=-3.16 eV) ν_1=-0.08

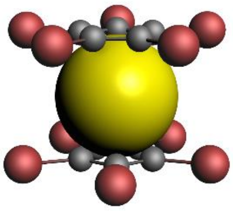

LUMO (ε=-17.8 eV) ν_1=+0.35

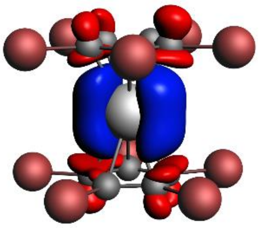

ΔE_2= -20.1; $|\nu_2|$= 0.29

ΔE_3= -20.1; $|\nu_3|$= 0.29

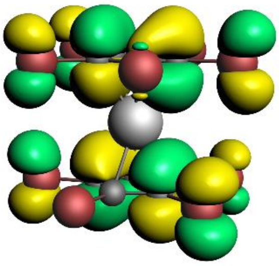

HOMO (ε=-1.01 eV) ν_2= -0.14

HOMO (ε= -1.01 eV) ν_3= -0.14

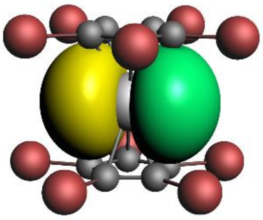

LUMO+1 (ε=-12.64 eV) ν_2=+ 0.24

LUMO+1 (ε=-12.64 eV) ν_3=+ 0.24

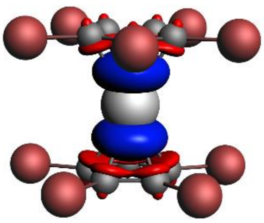

ΔE_4= -17.4; $|\nu_4|$= 0.21

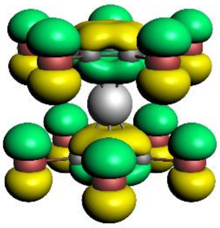

HOMO-1 (ε=-3.16 eV) ν_4=-0.08

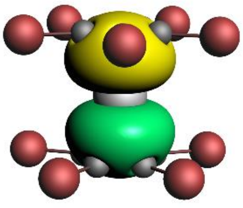

LUMO+1 (ε=-12.64 eV) ν_4=+ 0.18

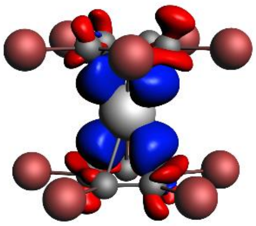

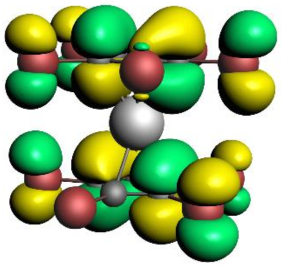

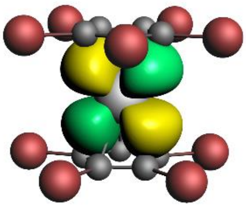

ΔE_5= -14.1; $|\nu_5|$= 0.19

ΔE_6= -14.0; $|\nu_6|$= 0.19

HOMO (ε=-1.01 eV) ν_5= -0.06

HOMO (ε= -1.01 eV) ν_6= -0.06

LUMO+4 (ε=-3.04 eV) ν_5=+ 0.08

LUMO+4 (ε=-3.04 eV) ν_6=+ 0.08

[Ca(C_5Br_5)$_2$]

**Deformation Densities**	**(C_5Br_5)$_2^{2-}$ orbitals**	**Ca^{2+} orbitals**
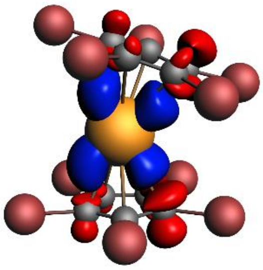	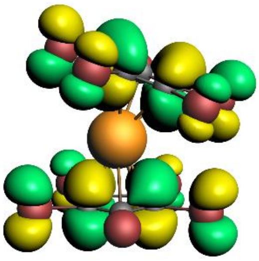	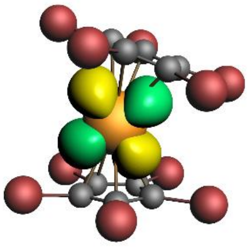
ΔE_1= -30.6; $|\nu_1|$= 0.47<br>ΔE_2= -28.5; $|\nu_2|$= 0.45	HOMO (ε=-1.04 eV) ν_1= -0.2<br>HOMO (ε=-1.04 eV) ν_2= -0.18	LUMO (ε=-15.91 eV) ν_1=+ 0.21<br>LUMO (ε=-15.91 eV) ν_2=+ 0.19
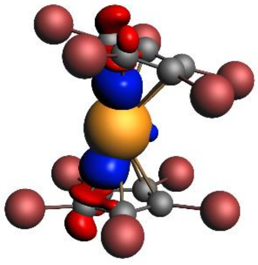	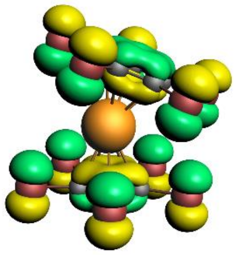	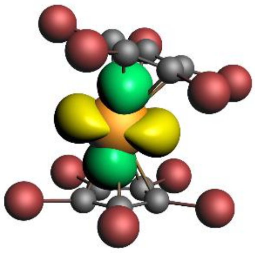
ΔE_3= -19.3; $|\nu_3|$= 0.33	HOMO-1 (ε=-3.18 eV) ν_3= -0.06	LUMO (ε=-15.91 eV) ν_3=+ 0.11
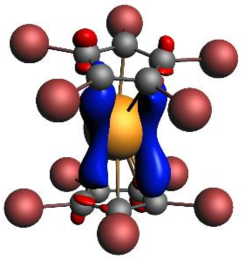	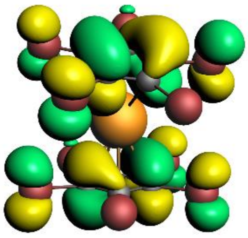	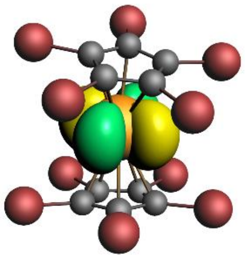

ΔE_4= -8.5; $|\nu_4|$= 0.21

HOMO (ε=-1.04 eV) ν_4= -0.04

LUMO (ε=-15.91 eV) ν_4=+ 0.03

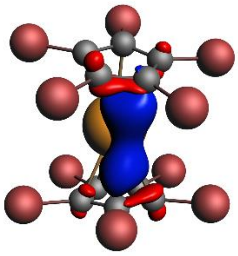

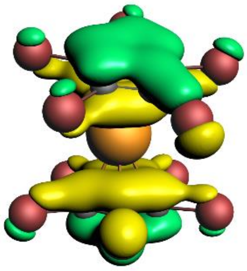

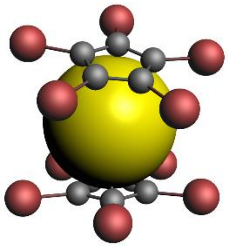

ΔE_5= -9.0; $|\nu_5|$= 0.20

HOMO-6 (ε=-6.47 eV) ν_5= - 0.04

LUMO+1 (ε=-14.24 eV) ν_5=+ 0.05

[$Sr(C_5Br_5)_2$]

**Deformation Densities**	**$(C_5Br_5)_2^{2-}$ orbitals**	**Sr^{2+} orbitals**

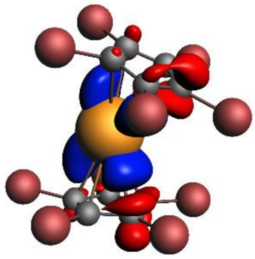

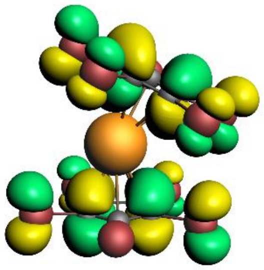

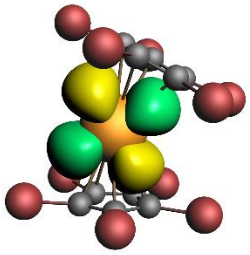

ΔE_1= -24.0; $|\nu_1|$= 0.41

HOMO (ε=-1.05 eV) ν_1= -0.16

LUMO (ε=-13.22 eV) ν_1=+ 0.20

ΔE_2= -21.6; $|\nu_2|$= 0.39

HOMO (ε=-1.05 eV) ν_2= -0.12

LUMO (ε=-13.22 eV) ν_2=+ 0.17

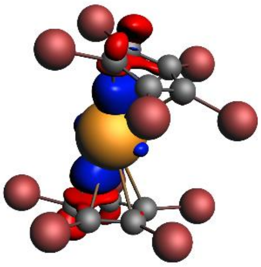

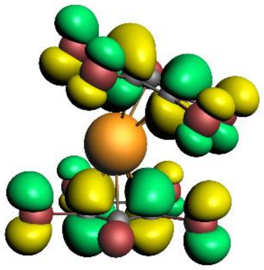

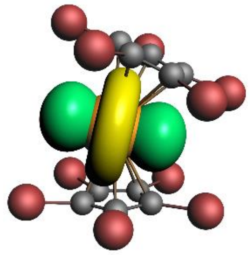

ΔE_3= -17.7; $|\nu_3|$= 0.34

HOMO (ε=-1.05 eV) ν_3= -0.08

LUMO (ε=-13.22 eV) ν_3=+ 0.07

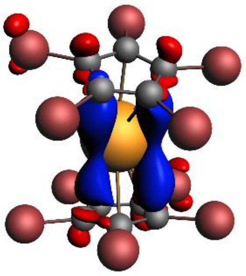

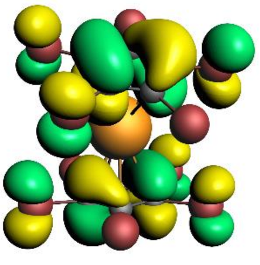

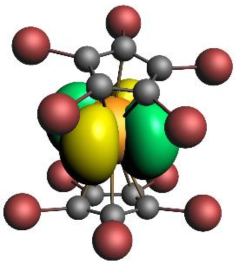

ΔE_4= -7.7; $|\nu_4|$= 0.21

HOMO (ε=-1.05 eV) ν_4= -0.02

LUMO (ε=-13.22 eV) ν_4=+ 0.04

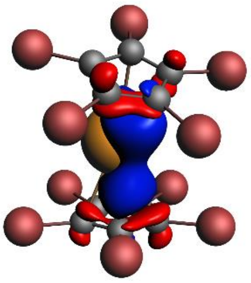

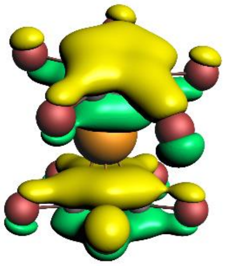

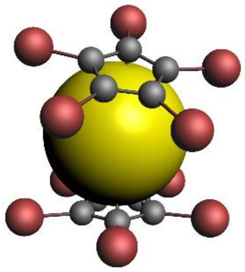

ΔE_5= -8.3; $|\nu_5|$= 0.19

HOMO-6 (ε=-6.49 eV) ν_5= -0.05

LUMO (ε=-13.22 eV) ν_5=+ 0.05

$[Ba(C_5Br_5)_2]$

Deformation Densities | **$(C_5Br_5)_2^{2-}$ orbitals** | **Ba^{2+} orbitals**

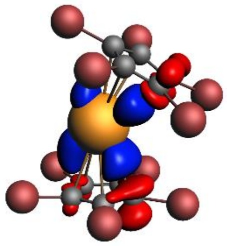

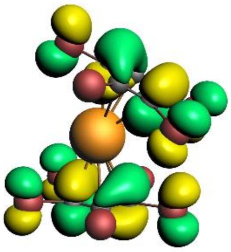

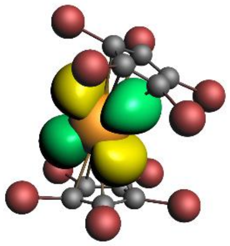

ΔE_1= -21.8; $|\nu_1|$= 0.41

ΔE_2= -19.4; $|\nu_2|$= 0.37

HOMO (ε=-1.05 eV) ν_1= -0.12

HOMO (ε=-1.05 eV) ν_2= -0.09

LUMO (ε=-12.82 eV) ν_1= +0.18

LUMO (ε=-12.82 eV) ν_2= +0.16

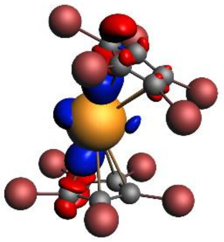

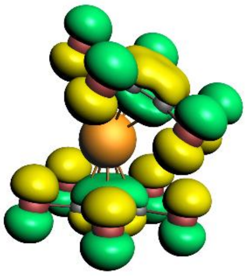

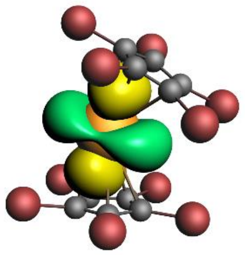

ΔE_3= -17.8; $|\nu_3|$= 0.37

HOMO-1 (ε=-3.12 eV) ν_3= -0.03

LUMO (ε=-12.82 eV) ν_3= +0.15

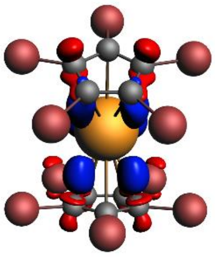

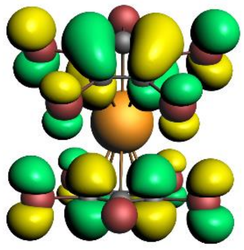

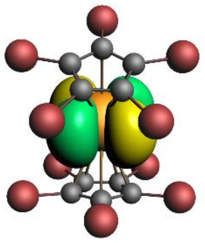

ΔE_4= -8.5; $|\nu_4|$= 0.25

HOMO (ε=-1.05 eV) ν_4= -0.04

LUMO (ε=-12.82 eV) ν_4= +0.05

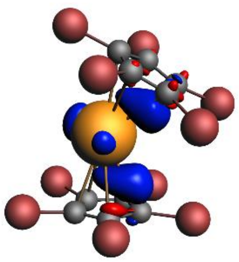

ΔE_5= -7.2; $|\nu_5|$= 0.20

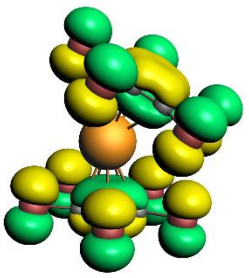

HOMO-1 (ε=-3.12 eV) ν_5= -0.02

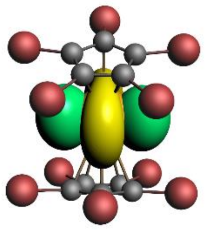

LUMO (ε=-12.82 eV) ν_5= +0.04

Figure S5. Plot of deformation densities (isovalue = 0.003) of the pairwise orbital interaction and shape of the most important occupied and vacant orbitals (isovalue = 0.03) in [Ae(C_5Br_5)$_2$] (Ae=Be-Ba) with the orbital interaction energies ΔE_{orb} (in kcal/mol) and their eigenvalues ν (in e). The direction of the charge flow is red→blue. The eigenvalues ν indicate the amount of donated (negative numbers) and accepted charge (positive numbers). The occupied orbitals are shown in yellow and blue for the different phases, while the unoccupied orbitals are in cyan and orange.

[Be(C_5I_5)$_2$]

**Deformation Densities**	**(C_5I_5)$_2^{2-}$ orbitals**	**Be^{2+} orbitals**
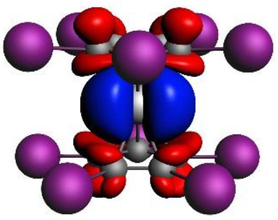	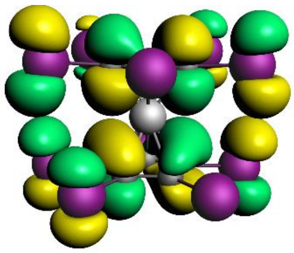	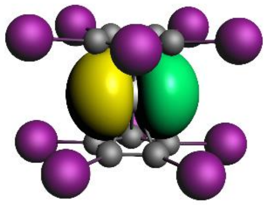
ΔE_1= -56.8; $|\nu_1|$= 0.47	HOMO (ε=-1.30 eV) ν_1= -0.24	LUMO+1 (ε=-17.36 eV) ν_1=+0.38
ΔE_2= -56.6; $|\nu_2|$= 0.47	HOMO (ε=-1.30 eV) ν_2= -0.24	LUMO+1 (ε=-17.36 eV) ν_2=+0.38

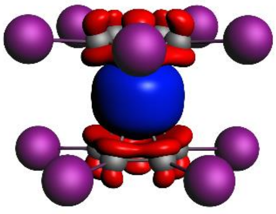

ΔE_3= -46.6; $|\nu_3|$= 0.38

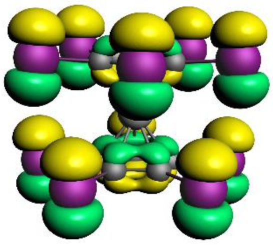

HOMO-2 (ε=-3.05 eV) ν_3= -0.10

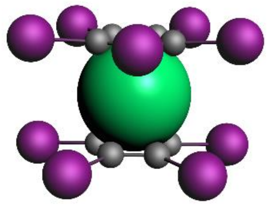

LUMO (ε=-21.09 eV) ν_3=+0.42

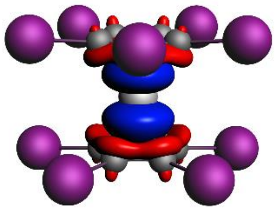

ΔE_4= -46.8; $|\nu_4|$= 0.37

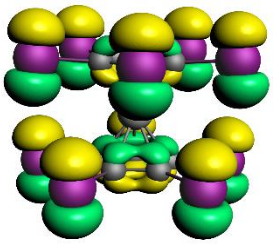

HOMO-2 (ε=-3.05 eV) ν_4= -0.08

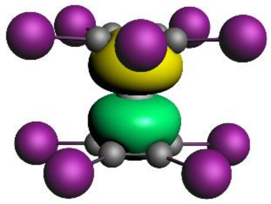

LUMO+1 (ε=-17.36 eV) ν_4=+0.32

[$Mg(C_5I_5)_2$]

**Deformation Densities**	**$(C_5I_5)_2^{2-}$ orbitals**	**Mg^{2+} orbitals**

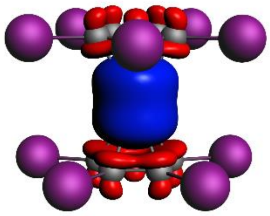

ΔE_1= -27.6; $|\nu_1|$= 0.33

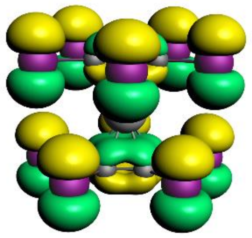

HOMO-2 (ε=-3.07 eV) ν_1=-0.04

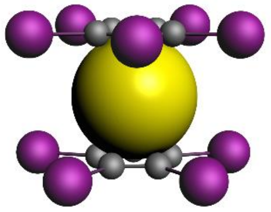

LUMO (ε=-17.8 eV) ν_1=+0.33

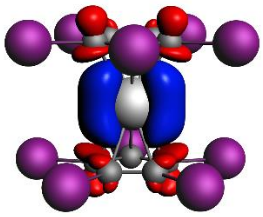

ΔE_2= -20.2; $|\nu_2|$= 0.29

ΔE_3= -20.1; $|\nu_3|$= 0.29

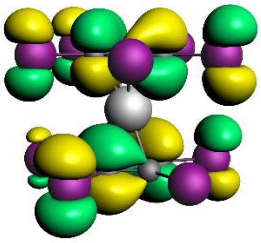

HOMO (ε=-1.32 eV) ν_2= -0.08

HOMO (ε= -1.32 eV) ν_3= -0.08

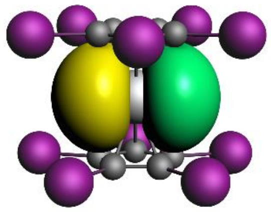

LUMO+1 (ε=-12.64 eV) ν_2=+0.23

LUMO+1 (ε=-12.64 eV) ν_3=+0.23

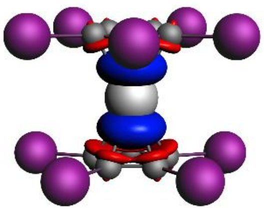

ΔE_4= -17.5; $|\nu_4|$= 0.21

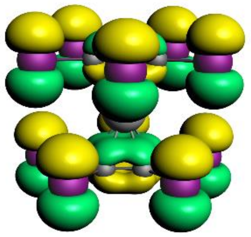

HOMO-2 (ε=-3.07 eV) ν_4=-0.04

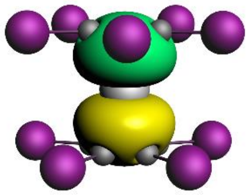

LUMO+1 (ε=-12.64 eV) ν_4=+0.24

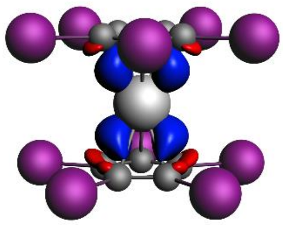

ΔE_5= -14.4; $|\nu_5|$= 0.19

ΔE_6= -14.3; $|\nu_6|$= 0.19

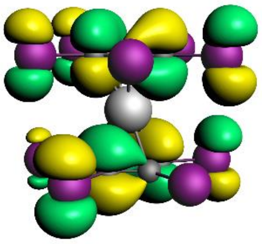

HOMO (ε=-1.32 eV) ν_5= -0.04

HOMO (ε= -1.32 eV) ν_6= -0.04

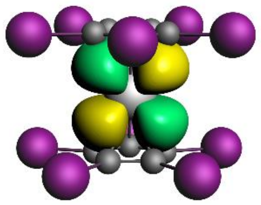

LUMO+3 (ε=-3.04 eV) ν_5=+ 0.08

LUMO+3 (ε=-3.04 eV) ν_6=+ 0.08

[$Ca(C_5I_5)_2$]

**Deformation Densities**	**$(C_5I_5)_2^{2-}$ orbitals**	**Ca^{2+} orbitals**

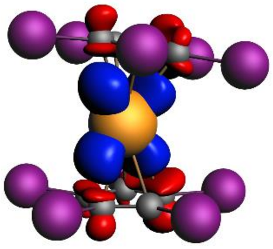

ΔE_1= -30.5; $|\nu_1|$= 0.46

ΔE_2= -29.6; $|\nu_2|$= 0.45

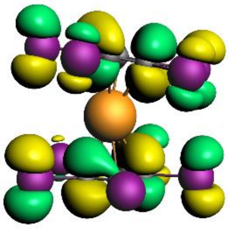

HOMO (ε=-1.33 eV) ν_1= -0.18

HOMO (ε=-1.33 eV) ν_2= -0.16

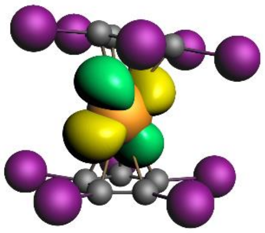

LUMO (ε=-15.91 eV) ν_1=+ 0.20

LUMO (ε=-15.91 eV) ν_2=+ 0.19

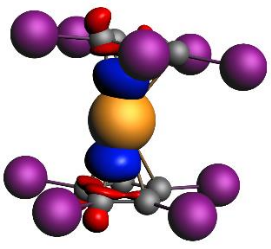

ΔE_3= -17.7; $|\nu_3|$= 0.31

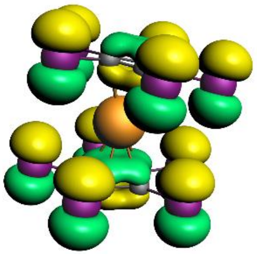

HOMO-2 (ε=-3.07 eV) ν_3= -0.06

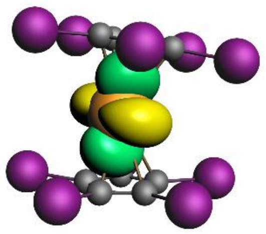

LUMO (ε=-15.91 eV) ν_3=+ 0.07

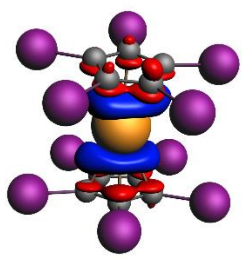

ΔE_4= -7.7; $|\nu_5|$= 0.17

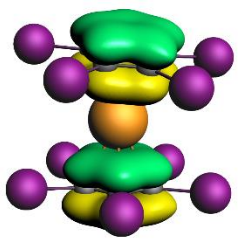

HOMO-9 (ε=-6.35 eV) ν_4= -0.1

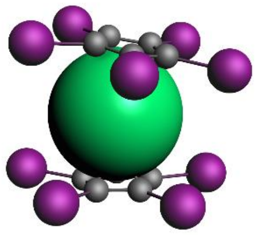

LUMO+1 (ε=-14.24 eV) ν_4=+ 0.12

[Sr(C_5I_5)$_2$]

**Deformation Densities**	**(C_5I_5)$_2^{2-}$ orbitals**	**Sr^{2+} orbitals**

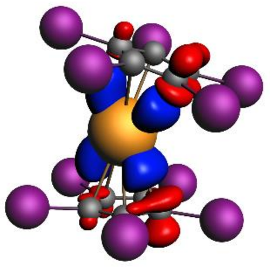

ΔE_1= -23.6; $|\nu_1|$= 0.40

ΔE_2= -22.1; $|\nu_2|$= 0.38

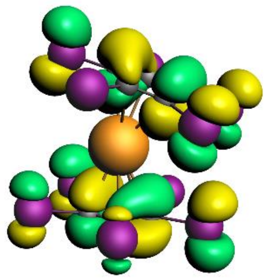

HOMO (ε=-1.33 eV) ν_1= -0.14

HOMO (ε=-1.33 eV) ν_2= -0.10

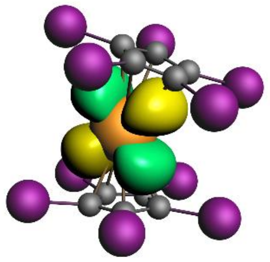

LUMO (ε=-13.22 eV) ν_1=+ 0.19

LUMO (ε=-13.22 eV) ν_2=+ 0.18

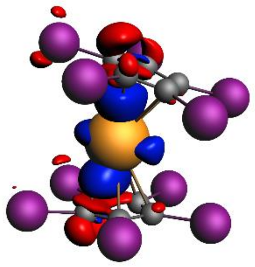

ΔE_3= -17.0; $|\nu_3|$= 0.33

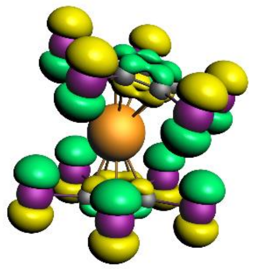

HOMO-2 (ε=-3.08 eV) ν_3= - 0.06

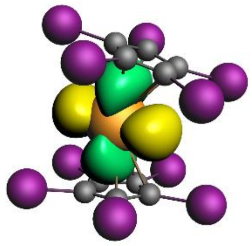

LUMO (ε=-13.22 eV) ν_3=+ 0.11

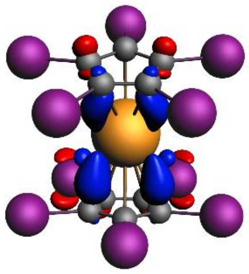

ΔE_4= -7.9; $|\nu_4|$= 0.22

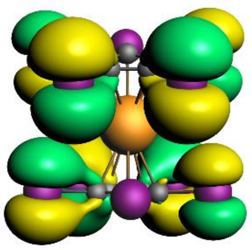

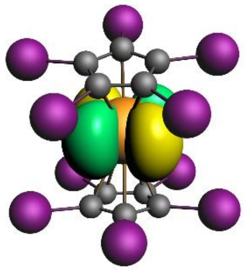

LUMO (ε=-13.22 eV) ν_4=+ 0.04

HOMO-4 (ε=-3.40 eV) ν_4= -0.03

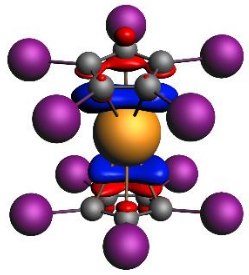
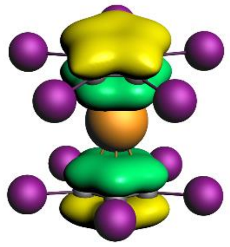
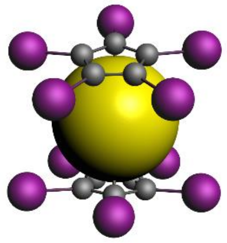

ΔE_5= -7.3; $|\nu_5|$= 0.18

HOMO-9 (ε=-6.36 eV) ν_5= -0.08

LUMO (ε=-13.22 eV) ν_5=+ 0.11

[Ba(C_5I_5)$_2$]

**Deformation Densities**	**(C_5I_5)$_2^{2-}$ orbitals**	**Ba^{2+} orbitals**

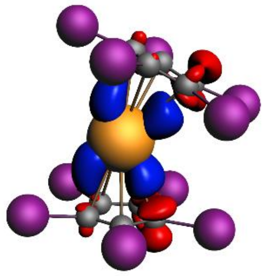
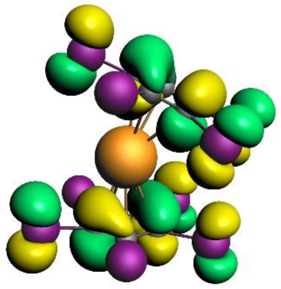
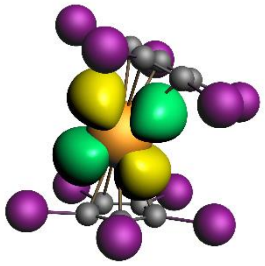

ΔE_1= -21.4; $|\nu_1|$= 0.40

ΔE_2= -20.0; $|\nu_2|$= 0.38

HOMO (ε=-1.34 eV) ν_1= -0.12

HOMO (ε=-1.34 eV) ν_2= -0.08

LUMO (ε=-12.82 eV) ν_1= +0.19

LUMO (ε=-12.82 eV) ν_2= +0.17

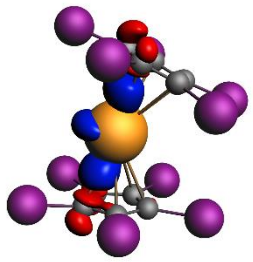
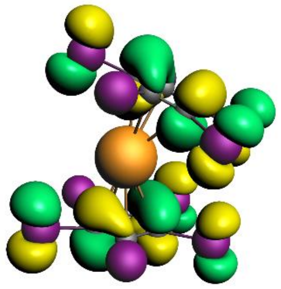
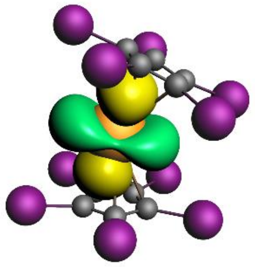

ΔE_3= -16.9; $|\nu_3|$= 0.38

HOMO (ε=-1.34 eV) ν_3= -0.06

LUMO (ε=-12.82 eV) ν_3= +0.14

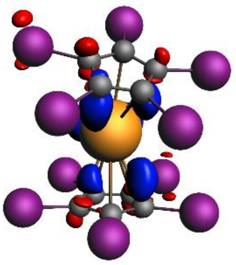
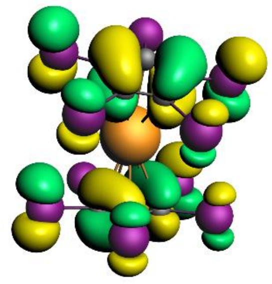
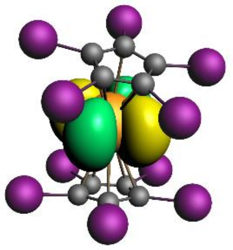

ΔE_4= -8.3; $|\nu_4|$= 0.22

HOMO (ε=-1.34 eV) ν_4= -0.02

LUMO (ε=-12.82 eV) ν_4= +0.05

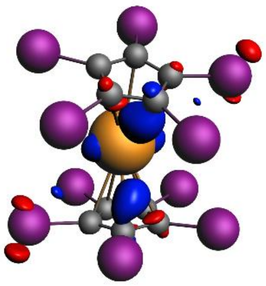

ΔE_5= -7.6; $|\nu_5|$= 0.22

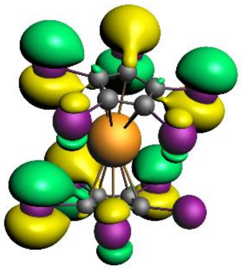

HOMO-4 (ε=-3.39 eV) ν_5= -0.03

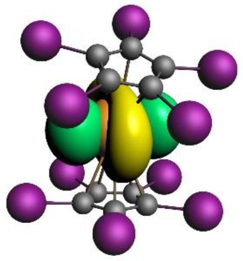

LUMO (ε=-12.82 eV) ν_5= +0.05

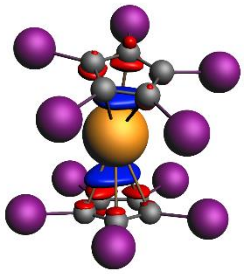

ΔE_6= -5.9; $|\nu_5|$= 0.16

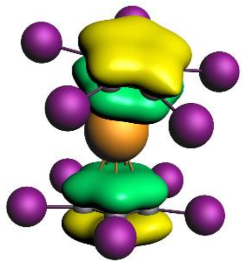

HOMO-9 (ε=-6.38 eV) ν_6= -0.06

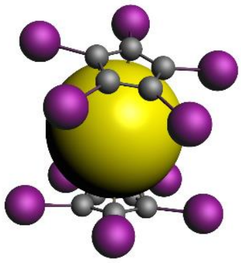

LUMO+1 (ε=-12.09 eV) ν_6= +0.09

Figure S6. Plot of deformation densities (isovalue = 0.003) of the pairwise orbital interaction and shape of the most important occupied and vacant orbitals (isovalue = 0.03) in $[Ae(C_5I_5)_2]$ (Ae=Be-Ba) with the orbital interaction energies ΔE_{orb} (in kcal/mol) and their eigenvalues *v* (in e). The direction of the charge flow is red→blue. The eigenvalues *v* indicate the amount of donated (negative numbers) and accepted charge (positive numbers). The occupied orbitals are shown in yellow and blue for the different phases, while the unoccupied orbitals are in cyan and orange.

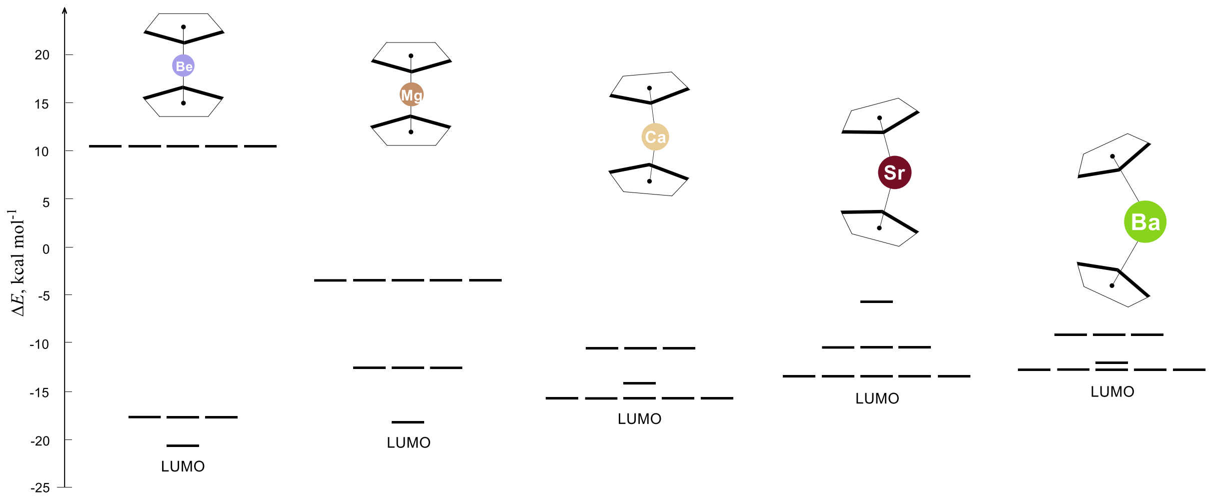

Figure S7. Energy of the orbitals for the group 2 at the BP86-D3(BJ)/TZ2P level of theory.

Table S3. Cp^X–Cp^X bond lengths $[Ae(Cp^X)_2]$ (Ae = Be – Ba ; X = H, Me, F, I) metallocenes optimized at B3LYP-D3(BJ)/def2-TZVPP level of theory.

	$[Ae(Cp)_2]$	$[Ae(Cp^*)_2]$	$[Ae(C_5I_5)_2]$	$[Ae(C_5F_5)_2]$
	Cp^X–Cp^X distance, Å			
Be	-	3.30	3.35	3.27
Mg	4.00	3.92	3.96	4.06
Ca	4.70	4.54	4.61	4.57
Sr	5.04	4.82	4.92	5.00
Ba	5.08	5.03	5.23	5.17

$[Be(Cp)_2]$

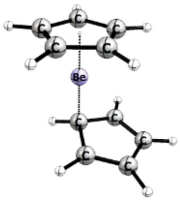

B3LYP-D3(BJ)/def2-tzvpp
Thermal correction to Gibbs Free Energy = 0.132740
Thermal correction to Enthalpy = 0.179136
Sum of electronic and thermal Free Energies = −401.884653
Sum of electronic and thermal Enthalpies = −401.838257
N_{imag} = 0

C	1.886210000	1.267213000	-0.046460000
C	1.662488000	0.287310000	0.953383000
C	1.729149000	-0.987519000	0.338966000
C	1.987997000	-0.796750000	-1.040608000
C	2.082215000	0.598059000	-1.278874000
C	-1.720500000	1.116994000	0.299138000
C	-1.319093000	0.184430000	-0.757548000
C	-1.704762000	-1.137095000	-0.255277000
C	-2.173299000	-0.999111000	1.024965000
C	-2.183126000	0.394421000	1.367826000
H	-1.594277000	-2.060091000	-0.804122000
H	-1.463986000	0.440599000	-1.804292000
H	-1.623521000	2.190602000	0.241927000
H	-2.489181000	0.798795000	2.321284000
H	-2.470756000	-1.803698000	1.681026000
H	2.207758000	1.067447000	-2.239477000
H	1.396831000	0.479328000	1.978066000
H	1.827192000	2.332781000	0.092442000
H	1.524001000	-1.927810000	0.819737000
H	2.026622000	-1.569508000	-1.788868000
Be	0.412135000	0.123699000	-0.461290000

[Mg(Cp)$_2$]

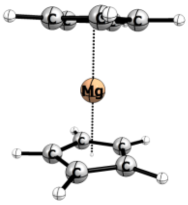

B3LYP-D3(BJ)/def2-tzvpp
Thermal correction to Gibbs Free Energy = 0.129458
Thermal correction to Enthalpy = 0.177326
Sum of electronic and thermal Free Energies = −587.256354
Sum of electronic and thermal Enthalpies = −587.208486
N_{imag} = 0

C	1.995346000	1.169789000	0.358804000
C	2.002968000	0.052104000	1.230783000
C	2.007352000	-1.122548000	0.437225000
C	2.002214000	-0.730807000	-0.925191000
C	1.994831000	0.685877000	-0.973664000
C	-2.007929000	1.174108000	-0.187579000
C	-2.004553000	0.143059000	-1.160448000
C	-1.995545000	-1.100774000	-0.480479000
C	-1.993789000	-0.838558000	0.912638000
C	-2.001148000	0.567428000	1.093659000
H	-2.004890000	-2.075187000	-0.941806000
H	-2.022029000	0.280534000	-2.229656000
H	-2.028630000	2.233447000	-0.386956000
H	-2.015329000	1.084522000	2.039599000
H	-2.000897000	-1.578506000	1.696737000
H	2.004048000	1.289399000	-1.866998000
H	2.019055000	0.089063000	2.308177000
H	2.004666000	2.205905000	0.656786000
H	2.026701000	-2.135753000	0.805251000
H	2.017820000	-1.393773000	-1.775261000
Mg	-0.000164000	0.000769000	0.030323000

[Ca(Cp)$_2$]

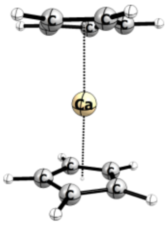

B3LYP-D3(BJ)/def2-tzvpp
Thermal correction to Gibbs Free Energy = 0.124512
Thermal correction to Enthalpy = 0.176616
Sum of electronic and thermal Free Energies = −1064.808771
Sum of electronic and thermal Enthalpies = −1064.756667
N_{imag} = 0

C	-2.370164000	-1.192279000	-0.074431000
C	-2.357828000	-0.291025000	-1.164679000
C	-2.334891000	1.023892000	-0.644206000
C	-2.333759000	0.935634000	0.767438000

C	-2.355517000	-0.434053000	1.119634000
H	-2.428178000	-2.268118000	-0.141809000
H	-2.404167000	-0.558805000	-2.209404000
H	-2.359901000	1.935027000	-1.222410000
H	-2.357615000	1.767405000	1.454940000
H	-2.399963000	-0.830170000	2.122834000
C	2.365135000	-0.809960000	-0.880789000
C	2.342891000	0.594151000	-1.050126000
C	2.330868000	1.188943000	0.233059000
C	2.346358000	0.152578000	1.195470000
C	2.367231000	-1.082993000	0.507192000
H	2.418268000	-1.543187000	-1.671274000
H	2.375065000	1.119863000	-1.992351000
H	2.352011000	2.247905000	0.441047000
H	2.381759000	0.282409000	2.266469000
H	2.422380000	-2.060826000	0.961076000
Ca	0.000053000	-0.029737000	-0.002951000

[Sr(Cp)$_2$]

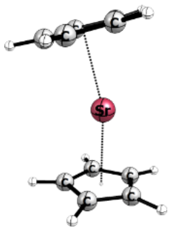

B3LYP-D3(BJ)/def2-tzvpp
Thermal correction to Gibbs Free Energy = 0.121802
Thermal correction to Enthalpy = 0.176212
Sum of electronic and thermal Free Energies = −417.924149
Sum of electronic and thermal Enthalpies = −417.869739
N_{imag} = 0

C	-2.650628000	-0.851319000	0.703861000
C	-2.471787000	0.480215000	1.145089000
C	-2.360642000	1.307538000	0.004068000
C	-2.470785000	0.487878000	-1.142563000
C	-2.650018000	-0.846578000	-0.710421000
C	2.649963000	-0.846232000	0.710905000
C	2.650679000	-0.851664000	-0.703376000
C	2.471876000	0.479655000	-1.145267000
C	2.360646000	1.307535000	-0.004657000
C	2.470700000	0.488434000	1.142382000
H	2.475239000	0.812957000	-2.172161000
H	2.820058000	-1.711658000	-1.334053000
H	2.818673000	-1.701363000	1.348337000
H	2.473012000	0.829600000	2.166692000
H	2.259364000	2.382211000	-0.008827000
H	-2.818785000	-1.702018000	-1.347424000
H	-2.475058000	0.814016000	2.171821000
H	-2.819955000	-1.711005000	1.334972000
H	-2.259359000	2.382215000	0.007705000
H	-2.473187000	0.828546000	-2.167038000
Sr	-0.000001000	-0.214639000	-0.000004000

[Ba(Cp)$_2$]

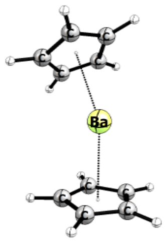

B3LYP-D3(BJ)/def2-tzvpp
Thermal correction to Gibbs Free Energy = 0.122750
Thermal correction to Enthalpy = 0.176007
Sum of electronic and thermal Free Energies = −412.701733
Sum of electronic and thermal Enthalpies = −412.648477
N_{imag} = 0

C	-2.339874000	0.922332000	1.077591000
C	-2.868951000	-0.365908000	0.838675000
C	-2.946259000	-0.553768000	-0.558987000
C	-2.465711000	0.619054000	-1.183923000
C	-2.091344000	1.531030000	-0.172566000
H	-2.197502000	1.381502000	2.044517000
H	-3.208511000	-1.061784000	1.591842000
H	-3.356792000	-1.417573000	-1.061025000
H	-2.438517000	0.806613000	-2.247147000
H	-1.716560000	2.531232000	-0.327058000
C	2.489893000	0.558352000	1.193568000
C	2.956714000	-0.581035000	0.500230000
C	2.851629000	-0.322208000	-0.884241000
C	2.318794000	0.976322000	-1.046629000
C	2.095850000	1.520511000	0.237704000
H	2.484751000	0.691527000	2.265319000
H	3.376734000	-1.469094000	0.949323000
H	3.175362000	-0.978585000	-1.678684000
H	2.157361000	1.484224000	-1.985748000
H	1.725280000	2.511632000	0.450256000
Ba	-0.000108000	-0.541210000	-0.000181000

[Be(Cp*)$_2$]

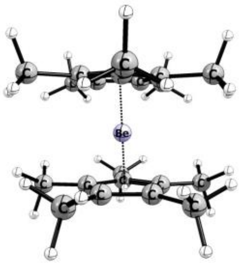

B3LYP-D3(BJ)/def2-tzvpp
Thermal correction to Gibbs Free Energy = 0.388992
Thermal correction to Enthalpy = 0.472463
Sum of electronic and thermal Free Energies = −795.006089
Sum of electronic and thermal Enthalpies = −794.922618
N_{imag} = 0

Be	0.000004000	-0.000008000	0.000111000
C	2.051148000	0.384420000	-0.133020000
C	-1.048961000	1.002696000	-1.398418000
C	1.819240000	-0.972812000	0.206174000

C	1.424974000	1.196910000	0.846944000
C	-1.819250000	0.972989000	-0.206106000
C	-0.803627000	-0.340655000	-1.795414000
C	1.049032000	-1.002776000	1.398348000
C	0.803673000	0.340603000	1.795750000
C	-2.051248000	-0.384222000	0.133293000
C	-1.425007000	-1.196890000	-0.846520000
C	-2.835951000	-0.874572000	1.308124000
H	-2.482990000	-1.845243000	1.654402000
H	-2.781496000	-0.184590000	2.149460000
H	-3.894155000	-0.990438000	1.057018000
C	1.439395000	2.691657000	0.892266000
H	0.545630000	3.089833000	1.371183000
H	2.298971000	3.062557000	1.457605000
H	1.499846000	3.126509000	-0.104690000
C	0.071991000	0.772273000	3.026279000
H	-0.693286000	0.052700000	3.314364000
H	0.753518000	0.874279000	3.875488000
H	-0.416950000	1.735940000	2.889756000
C	0.613275000	-2.227442000	2.137362000
H	1.332483000	-2.495062000	2.916564000
H	-0.349753000	-2.085335000	2.626306000
H	0.522610000	-3.087532000	1.475673000
C	-2.317411000	2.165442000	0.546331000
H	-3.292612000	2.491074000	0.173329000
H	-2.436090000	1.952616000	1.607953000
H	-1.640517000	3.014102000	0.454038000
C	-0.613553000	2.226425000	-2.139190000
H	-1.334885000	2.494783000	-2.916181000
H	-0.519479000	3.086663000	-1.478150000
H	0.347610000	2.082515000	-2.631257000
C	-0.071360000	-0.772061000	-3.025669000
H	0.698077000	-0.055448000	-3.310069000
H	0.412565000	-1.738477000	-2.890848000
H	-0.751555000	-0.868329000	-3.876599000
C	2.316858000	-2.165226000	-0.546630000
H	3.293535000	-2.489295000	-0.176182000
H	1.641218000	-3.014528000	-0.451542000
H	2.432122000	-1.953417000	-1.608817000
C	2.836303000	0.875053000	-1.307413000
H	2.479604000	1.842948000	-1.657728000
H	3.893279000	0.996948000	-1.053933000
H	2.787498000	0.182121000	-2.146644000
C	-1.439512000	-2.691628000	-0.892041000
H	-0.544803000	-3.089932000	-1.369046000
H	-1.502170000	-3.126717000	0.104706000
H	-2.297914000	-3.062306000	-1.459300000

[Mg(Cp*)$_2$]

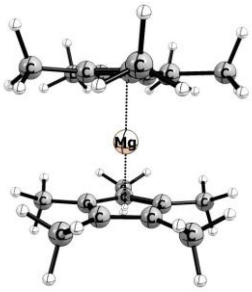

B3LYP-D3(BJ)/def2-tzvpp
Thermal correction to Gibbs Free Energy = 0.379227
Thermal correction to Enthalpy = 0.470309
Sum of electronic and thermal Free Energies = −980.376408
Sum of electronic and thermal Enthalpies = −980.285326
N_{imag} = 0

Mg	0.000334000	-0.002607000	-0.001144000
C	-1.960777000	-0.554909000	-1.075673000
C	1.959488000	-1.043419000	-0.621924000
C	-1.956844000	0.854554000	-0.862976000
C	-1.959912000	-1.192347000	0.198993000
C	1.958424000	-0.915998000	0.797597000
C	1.960642000	0.267381000	-1.181334000
C	-1.955592000	1.087411000	0.543037000
C	-1.956735000	-0.177500000	1.198895000
C	1.957553000	0.472911000	1.115137000
C	1.958966000	1.203999000	-0.107689000
C	2.001378000	1.065432000	2.491322000
H	1.468719000	2.016207000	2.539526000
H	1.551332000	0.402876000	3.231085000
H	3.028122000	1.257721000	2.816412000
C	-2.023947000	-2.669765000	0.443436000
H	-1.525266000	-2.950214000	1.372080000
H	-3.057743000	-3.020007000	0.517873000
H	-1.554236000	-3.234482000	-0.362702000
C	-2.001174000	-0.405101000	2.679994000
H	-1.526727000	0.408810000	3.228922000
H	-3.029081000	-0.482218000	3.046226000
H	-1.492162000	-1.327677000	2.962062000
C	-2.000704000	2.428019000	1.211970000
H	-3.028837000	2.773192000	1.355457000
H	-1.532671000	2.404679000	2.196561000
H	-1.487380000	3.191340000	0.626077000
C	2.006661000	-2.045720000	1.781703000
H	3.035961000	-2.319332000	2.031641000
H	1.509114000	-1.789616000	2.717917000
H	1.524298000	-2.942227000	1.391124000
C	2.012665000	-2.328834000	-1.391810000
H	3.043118000	-2.641889000	-1.583777000
H	1.527191000	-3.143789000	-0.853572000
H	1.520819000	-2.241070000	-2.361323000
C	2.010468000	0.603079000	-2.641296000
H	1.559701000	-0.180781000	-3.250280000
H	1.483092000	1.531740000	-2.863043000
H	3.039181000	0.728444000	-2.991859000
C	-2.008044000	1.905839000	-1.930604000

H	-3.037697000	2.137450000	-2.218845000
H	-1.552894000	2.839990000	-1.600023000
H	-1.486787000	1.591924000	-2.835943000
C	-2.015362000	-1.246985000	-2.404174000
H	-1.487145000	-2.201219000	-2.386691000
H	-3.045354000	-1.457331000	-2.707129000
H	-1.568469000	-0.641442000	-3.193160000
C	2.015064000	2.696108000	-0.241136000
H	1.549382000	3.037446000	-1.166251000
H	1.508501000	3.197280000	0.584693000
H	3.046600000	3.060553000	-0.250131000

[Ca(Cp*)$_2$]

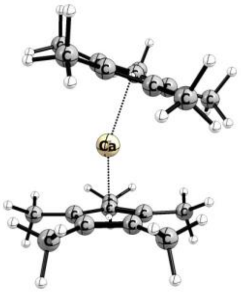

B3LYP-D3(BJ)/def2-tzvpp
Thermal correction to Gibbs Free Energy = 0.383150
Thermal correction to Enthalpy = 0.468400
Sum of electronic and thermal Free Energies = −1457.911950
Sum of electronic and thermal Enthalpies = −1457.826700
N_{imag} = 1, ν = -8.4863i cm^{-1}

Ca	0.000847000	0.006645000	-0.397769000
C	2.114623000	0.857073000	0.874891000
C	-2.092698000	0.773717000	0.955361000
C	2.383308000	1.069227000	-0.503040000
C	2.065105000	-0.543684000	1.101563000
C	-2.076535000	-0.643140000	1.044955000
C	-2.357444000	1.125083000	-0.395056000
C	2.499150000	-0.201102000	-1.129119000
C	2.301029000	-1.198562000	-0.136372000
C	-2.328642000	-1.167608000	-0.249537000
C	-2.503384000	-0.074673000	-1.140774000
C	-2.480889000	-2.620297000	-0.594493000
H	-2.213411000	-2.825995000	-1.632519000
H	-1.858155000	-3.257947000	0.035468000
H	-3.512772000	-2.961091000	-0.463132000
C	1.879382000	-1.217171000	2.428554000
H	1.370567000	-2.177492000	2.332331000
H	2.837753000	-1.413685000	2.919266000
H	1.290893000	-0.607991000	3.115291000
C	2.412342000	-2.681448000	-0.337966000
H	2.152348000	-2.977688000	-1.355682000
H	3.430967000	-3.039302000	-0.157584000
H	1.761329000	-3.236468000	0.339840000
C	2.869098000	-0.446955000	-2.562958000
H	3.953451000	-0.516310000	-2.695231000
H	2.449131000	-1.380742000	-2.941538000
H	2.525982000	0.355248000	-3.219077000

C	-1.907072000	-1.447024000	2.299703000
H	-2.869989000	-1.667930000	2.770634000
H	-1.421679000	-2.405589000	2.108996000
H	-1.303777000	-0.922385000	3.040699000
C	-1.936933000	1.729814000	2.100539000
H	-2.901297000	1.980616000	2.553394000
H	-1.313356000	1.314688000	2.893123000
H	-1.480155000	2.671083000	1.790544000
C	-2.544850000	2.519549000	-0.917440000
H	-1.937285000	3.245282000	-0.373850000
H	-2.284137000	2.601735000	-1.974199000
H	-3.584323000	2.849964000	-0.825071000
C	2.603064000	2.399810000	-1.161831000
H	3.654031000	2.703133000	-1.120768000
H	2.323697000	2.387432000	-2.217097000
H	2.029506000	3.194318000	-0.681401000
C	1.985291000	1.924944000	1.920388000
H	1.347215000	1.610469000	2.746925000
H	2.955942000	2.189860000	2.351146000
H	1.559348000	2.844652000	1.515549000
C	-2.883575000	-0.172860000	-2.589605000
H	-2.538884000	0.689228000	-3.163557000
H	-2.472846000	-1.066476000	-3.063626000
H	-3.969247000	-0.221661000	-2.720058000

[Sr(Cp*)$_2$]

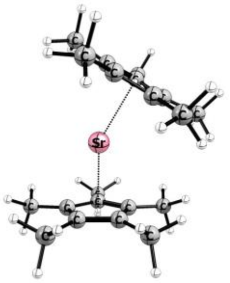

B3LYP-D3(BJ)/def2-tzvpp
Thermal correction to Gibbs Free Energy = 0.383156
Thermal correction to Enthalpy = 0.468047
Sum of electronic and thermal Free Energies = −811.023022
Sum of electronic and thermal Enthalpies = −810.938130
N_{imag} = 1, v = −5.8962i cm^{-1}

Sr	-0.000095000	-0.001131000	-0.540138000
C	-2.161029000	-0.757515000	1.024966000
C	2.140172000	-0.668518000	1.090678000
C	-2.530474000	-1.123163000	-0.295506000
C	-2.137165000	0.659879000	1.096866000
C	2.159508000	0.749522000	1.030887000
C	2.495264000	-1.166207000	-0.190046000
C	-2.735627000	0.067792000	-1.040185000
C	-2.491657000	1.169658000	-0.179287000
C	2.526512000	1.127615000	-0.286775000
C	2.734975000	-0.056271000	-1.041722000
C	3.221963000	-0.121212000	-2.460108000
H	2.872379000	-1.018289000	-2.975913000
H	2.897121000	0.740937000	-3.046593000

H	4.315276000	-0.138443000	-2.515241000
C	2.752199000	2.531723000	-0.766982000
H	2.556862000	2.639104000	-1.836232000
H	2.117064000	3.250343000	-0.245344000
H	3.785600000	2.855756000	-0.606426000
C	1.934082000	1.683804000	2.182602000
H	2.866990000	1.913179000	2.707346000
H	1.512410000	2.638026000	1.861137000
H	1.250280000	1.263645000	2.920410000
C	1.893556000	-1.495648000	2.317802000
H	2.826929000	-1.741154000	2.834375000
H	1.261800000	-0.974780000	3.037863000
H	1.403887000	-2.443477000	2.086534000
C	2.677528000	-2.611596000	-0.551194000
H	2.016194000	-3.263635000	0.022958000
H	2.484109000	-2.799384000	-1.609524000
H	3.698878000	-2.954754000	-0.356694000
C	-3.223509000	0.145938000	-2.457616000
H	-4.316845000	0.164333000	-2.511940000
H	-2.873623000	1.047422000	-2.965435000
H	-2.899523000	-0.711005000	-3.052173000
C	-2.762312000	-2.522236000	-0.787320000
H	-3.797275000	-2.842647000	-0.629600000
H	-2.567256000	-2.621903000	-1.857368000
H	-2.130748000	-3.248100000	-0.271433000
C	-1.936575000	-1.702079000	2.168484000
H	-1.247712000	-1.291539000	2.907036000
H	-2.868752000	-1.930461000	2.694970000
H	-1.521454000	-2.656020000	1.837834000
C	-2.669153000	2.618743000	-0.527774000
H	-2.476084000	2.814948000	-1.584638000
H	-3.689061000	2.963850000	-0.329243000
H	-2.004827000	3.263361000	0.051242000
C	-1.887652000	1.475484000	2.331058000
H	-1.396321000	2.424346000	2.107871000
H	-2.820084000	1.718091000	2.850687000
H	-1.256246000	0.946939000	3.045794000

[Ba(Cp*)$_2$]

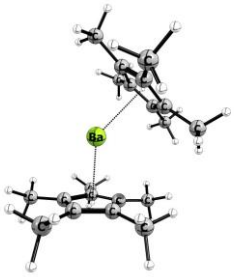

B3LYP-D3(BJ)/def2-tzvpp
Thermal correction to Gibbs Free Energy = 0.377556
Thermal correction to Enthalpy = 0.468676
Sum of electronic and thermal Free Energies = −805.809770
Sum of electronic and thermal Enthalpies = −805.718650
N_{imag} = 0

Ba	0.000082000	0.000417000	-0.689554000

C	2.722476000	-1.054004000	-0.305520000
C	-2.567231000	-1.196647000	0.098692000
C	2.228824000	-0.880493000	1.012319000
C	2.932859000	0.228977000	-0.868320000
C	-2.932726000	-0.227318000	-0.868887000
C	-2.135684000	-0.514093000	1.263812000
C	2.135513000	0.511977000	1.264786000
C	2.567207000	1.196618000	0.100904000
C	-2.722253000	1.054658000	-0.303882000
C	-2.228822000	0.878808000	1.013761000
C	3.496954000	0.518239000	-2.228060000
H	3.107792000	1.450124000	-2.646257000
H	4.586559000	0.623005000	-2.207530000
H	3.277277000	-0.277975000	-2.943143000
C	3.025963000	-2.370386000	-0.957981000
H	2.961791000	-2.315045000	-2.047071000
H	4.036431000	-2.721773000	-0.724967000
H	2.343617000	-3.158034000	-0.629055000
C	1.958833000	-1.978990000	1.997707000
H	2.873455000	-2.305805000	2.502922000
H	1.261960000	-1.664276000	2.773784000
H	1.533674000	-2.867224000	1.523449000
C	1.761171000	1.153081000	2.567577000
H	2.643227000	1.377575000	3.175887000
H	1.229158000	2.095648000	2.426131000
H	1.119795000	0.507747000	3.168095000
C	2.683800000	2.684699000	-0.049049000
H	1.953113000	3.215133000	0.565205000
H	3.669807000	3.052662000	0.253228000
H	2.539158000	3.008064000	-1.082958000
C	-3.025677000	2.372114000	-0.954144000
H	-2.344802000	3.159741000	-0.622193000
H	-2.959278000	2.319100000	-2.043227000
H	-4.036979000	2.721991000	-0.722450000
C	-1.958699000	1.975721000	2.000863000
H	-1.262290000	1.659651000	2.776787000
H	-1.532950000	2.864376000	1.527909000
H	-2.873389000	2.302264000	2.506139000
C	-1.762153000	-1.157651000	2.565618000
H	-1.230147000	-2.100023000	2.422817000
H	-1.121102000	-0.513541000	3.167795000
H	-2.644613000	-1.383198000	3.172950000
C	-2.683986000	-2.684482000	-0.053869000
H	-2.540669000	-3.005874000	-1.088575000
H	-1.952446000	-3.216050000	0.558402000
H	-3.669562000	-3.053115000	0.249035000
C	-3.496749000	-0.513926000	-2.229193000
H	-4.586344000	-0.618898000	-2.208953000
H	-3.277176000	0.283809000	-2.942632000
H	-3.107396000	-1.444843000	-2.649294000

[Be(5F-Cp)$_2$]

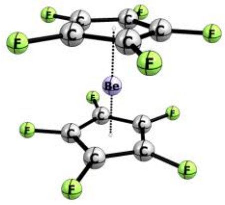

B3LYP-D3(BJ)/def2-tzvpp
Thermal correction to Gibbs Free Energy = 0.034766
Thermal correction to Enthalpy = 0.106836
Sum of electronic and thermal Free Energies = −1394.645772
Sum of electronic and thermal Enthalpies = −1394.573703
N_{imag} = 0

C	1.616879000	0.678351000	-1.015559000
C	1.638496000	1.157694000	0.314631000
C	1.655484000	0.040859000	1.181397000
C	1.644159000	-1.128833000	0.387299000
C	1.620487000	-0.735153000	-0.970664000
C	-1.655482000	-0.040809000	-1.181399000
C	-1.638557000	-1.157654000	-0.314606000
C	-1.616913000	-0.678250000	1.015604000
C	-1.620456000	0.735246000	0.970608000
C	-1.644096000	1.128872000	-0.387330000
Be	0.000024000	-0.000775000	0.000029000
F	1.617869000	-2.378229000	0.838196000
F	-1.606508000	-2.433170000	-0.684965000
F	1.574635000	-1.549664000	-2.019295000
F	1.637194000	0.083088000	2.509191000
F	1.606047000	2.433165000	0.685048000
F	1.567654000	1.424645000	-2.113552000
F	-1.637168000	-0.083137000	-2.509191000
F	-1.567945000	-1.424545000	2.113628000
F	-1.574378000	1.549750000	2.019217000
F	-1.617411000	2.378227000	-0.838278000

[Mg(5F-Cp)$_2$]

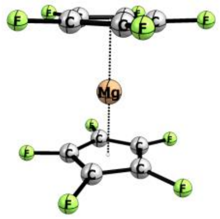

B3LYP-D3(BJ)/def2-tzvpp
Thermal correction to Gibbs Free Energy = 0.031344
Thermal correction to Enthalpy = 0.105096
Sum of electronic and thermal Free Energies = −1580.009878
Sum of electronic and thermal Enthalpies = −1579.936126
N_{imag} = 0

C	-2.028159000	0.777930000	0.919180000
C	-2.028881000	1.114149000	-0.456492000
C	-2.028770000	-0.090252000	-1.201156000
C	-2.028319000	-1.170753000	-0.285830000
C	-2.028112000	-0.634265000	1.024817000
C	2.029073000	0.090383000	1.201113000

C	2.028770000	-1.114172000	0.456654000
C	2.028517000	-0.777987000	-0.919033000
C	2.027664000	0.634076000	-1.024866000
C	2.028303000	1.170749000	0.285527000
F	-2.092674000	-2.465820000	-0.602387000
F	2.096928000	-2.347548000	0.962117000
F	-2.095225000	-1.335497000	2.158388000
F	-2.094770000	-0.190019000	-2.530552000
F	-2.097129000	2.347567000	-0.961715000
F	-2.095638000	1.639723000	1.936043000
F	2.095313000	0.190543000	2.530535000
F	2.096170000	-1.639890000	-1.935811000
F	2.094351000	1.335152000	-2.158457000
F	2.092690000	2.465865000	0.601844000
Mg	-0.000055000	0.000014000	0.000039000

[Ca(5F-Cp)$_2$]

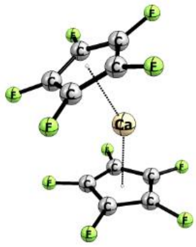

B3LYP-D3(BJ)/def2-tzvpp
Thermal correction to Gibbs Free Energy = 0.029247
Thermal correction to Enthalpy = 0.104924
Sum of electronic and thermal Free Energies = −2057.571438
Sum of electronic and thermal Enthalpies = −2057.495761
N_{imag} = 0

C	2.081793000	0.887658000	0.847077000
C	2.012485000	-0.494062000	1.123821000
C	2.324626000	-1.191064000	-0.061342000
C	2.584600000	-0.241491000	-1.070519000
C	2.436470000	1.042851000	-0.508420000
C	-2.081788000	0.887632000	0.847102000
C	-2.436465000	1.042865000	-0.508391000
C	-2.584601000	-0.241460000	-1.070526000
C	-2.324632000	-1.191063000	-0.061375000
C	-2.012487000	-0.494096000	1.123808000
F	2.945031000	-0.512333000	-2.335442000
F	-2.641010000	2.207284000	-1.142680000
F	2.641017000	2.207251000	-1.142745000
F	2.409483000	-2.522496000	-0.195040000
F	1.780009000	-1.044705000	2.319777000
F	1.924191000	1.876410000	1.734670000
F	-1.924183000	1.876359000	1.734723000
F	-2.945036000	-0.512266000	-2.335455000
F	-2.409494000	-2.522490000	-0.195111000
F	-1.780010000	-1.044774000	2.319748000
Ca	0.000000000	-0.006039000	-0.541471000

[Sr(5F-Cp)$_2$]

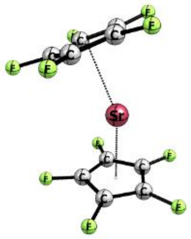

B3LYP-D3(BJ)/def2-tzvpp
Thermal correction to Gibbs Free Energy = 0.022984
Thermal correction to Enthalpy = 0.104527
Sum of electronic and thermal Free Energies = −1410.697190
Sum of electronic and thermal Enthalpies = −1410.615647
N_{imag} = 0

C	-2.514529000	-1.197102000	0.025791000
C	-2.270711000	-0.448773000	1.194578000
C	-2.345759000	0.918890000	0.858423000
C	-2.636827000	1.012755000	-0.516159000
C	-2.740877000	-0.293720000	-1.029953000
C	2.514619000	-1.197352000	0.023556000
C	2.740716000	-0.292245000	-1.030762000
C	2.636582000	1.013396000	-0.514869000
C	2.345770000	0.917287000	0.859613000
C	2.270903000	-0.450940000	1.193581000
F	-2.791394000	2.150134000	-1.216235000
F	2.982919000	-0.617287000	-2.313845000
F	-2.984049000	-0.620830000	-2.312312000
F	-2.263897000	1.945479000	1.717373000
F	-2.129590000	-0.946431000	2.430593000
F	-2.574161000	-2.536335000	-0.056605000
F	2.573643000	-2.536484000	-0.061191000
F	2.791379000	2.151907000	-1.213021000
F	2.264960000	1.942403000	1.720388000
F	2.130396000	-0.950566000	2.428868000
Sr	-0.000031000	0.007077000	-0.434182000

[Ba(5F-Cp)$_2$]

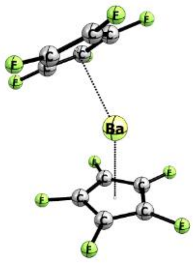

B3LYP-D3(BJ)/def2-tzvpp
Thermal correction to Gibbs Free Energy = 0.023006
Thermal correction to Enthalpy = 0.104591
Sum of electronic and thermal Free Energies = −1405.485637
Sum of electronic and thermal Enthalpies = −1405.404053
N_{imag} = 0

C	2.311169000	1.051598000	-0.835279000
C	2.247490000	1.243336000	0.559288000
C	2.647772000	0.045628000	1.179490000
C	2.960569000	-0.882962000	0.171645000
C	2.753043000	-0.262476000	-1.072064000

C	-2.647557000	0.048257000	-1.179555000
C	-2.960548000	-0.882554000	-0.173824000
C	-2.753227000	-0.264819000	1.071296000
C	-2.311291000	1.049763000	0.837496000
C	-2.247370000	1.244588000	-0.556632000
F	3.299888000	-2.173143000	0.363596000
F	-3.299964000	-2.172271000	-0.368671000
F	2.918801000	-0.837785000	-2.277454000
F	2.724039000	-0.173504000	2.504329000
F	1.983992000	2.401038000	1.181345000
F	2.105112000	1.992766000	-1.768495000
F	-2.723827000	-0.167860000	-2.504882000
F	-2.919035000	-0.842844000	2.275384000
F	-2.105227000	1.988820000	1.772838000
F	-1.983805000	2.403667000	-1.176087000
Ba	-0.000001000	-0.644859000	-0.000505000

[Be(5Cl-Cp)$_2$]

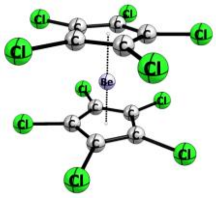

B3LYP-D3(BJ)/def2-tzvpp
Thermal correction to Gibbs Free Energy = 0.018938
Thermal correction to Enthalpy = 0.096247
Sum of electronic and thermal Free Energies = −4998.235540
Sum of electronic and thermal Enthalpies = −4998.158231
N_{imag} = 0

C	1.010339000	0.634929000	1.644716000
C	-0.302218000	1.168408000	1.638740000
C	-1.215029000	0.085075000	1.633622000
C	-0.467149000	-1.118018000	1.636162000
C	0.908354000	-0.778372000	1.643011000
C	1.215031000	-0.084997000	-1.633600000
C	0.302239000	-1.168371000	-1.638883000
C	-1.010382000	-0.634851000	-1.644773000
C	-0.908299000	0.778438000	-1.642959000
C	0.467134000	1.118055000	-1.635987000
Be	-0.000096000	-0.001257000	0.000075000
Cl	-1.109691000	-2.698741000	1.640119000
Cl	0.711287000	-2.824947000	-1.647299000
Cl	2.213115000	-1.877826000	1.663088000
Cl	2.916916000	-0.207772000	-1.630962000
Cl	2.459426000	1.535532000	1.667097000
Cl	1.109672000	2.698740000	-1.639919000
Cl	-0.711269000	2.824940000	1.647073000
Cl	-2.213047000	1.877896000	-1.662688000
Cl	-2.916910000	0.207827000	1.630963000
Cl	-2.459482000	-1.535458000	-1.667506000

[Mg(5Cl-Cp)$_2$]

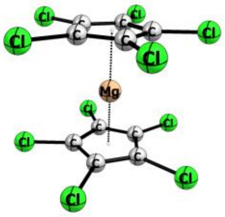

B3LYP-D3(BJ)/def2-tzvpp
Thermal correction to Gibbs Free Energy = 0.011867
Thermal correction to Enthalpy = 0.095271
Sum of electronic and thermal Free Energies = −5183.610906
Sum of electronic and thermal Enthalpies = −5183.527502
N_{imag} = 0

C	-1.985970000	1.067742000	0.564465000
C	-1.986211000	0.867544000	-0.840168000
C	-1.987328000	-0.530208000	-1.083866000
C	-1.987725000	-1.193799000	0.170254000
C	-1.986837000	-0.206206000	1.188916000
C	1.987015000	0.530110000	1.083932000
C	1.986349000	-0.867630000	0.840101000
C	1.986351000	-1.067654000	-0.564562000
C	1.986954000	0.206395000	-1.188875000
C	1.987296000	1.193863000	-0.170096000
Mg	-0.000008000	-0.000375000	-0.000178000
Cl	2.010395000	1.283705000	2.622122000
Cl	-2.008106000	2.581781000	1.365558000
Cl	-2.010553000	-0.500394000	2.876333000
Cl	2.008929000	-2.097644000	2.032131000
Cl	-2.012438000	-2.889574000	0.411881000
Cl	2.009210000	-2.581585000	-1.365872000
Cl	-2.011132000	-1.283963000	-2.621971000
Cl	2.010754000	0.500791000	-2.876257000
Cl	-2.008361000	2.097405000	-2.032367000
Cl	2.011346000	2.889688000	-0.411468000

[Ca(5Cl-Cp)$_2$]

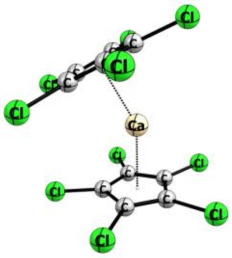

B3LYP-D3(BJ)/def2-tzvpp
Thermal correction to Gibbs Free Energy = 0.010238
Thermal correction to Enthalpy = 0.095306
Sum of electronic and thermal Free Energies = −5661.178312
Sum of electronic and thermal Enthalpies = −5661.093244
N_{imag} = 0

C	2.036977000	0.706650000	1.013361000
C	2.036890000	-0.706128000	1.013731000
C	2.349021000	-1.141954000	-0.292191000
C	2.542387000	-0.000324000	-1.099484000
C	2.349165000	1.141753000	-0.292788000

C	-2.182833000	1.142362000	0.397270000
C	-2.471041000	0.705424000	-0.913686000
C	-2.470945000	-0.706056000	-0.913236000
C	-2.182676000	-1.142121000	0.397997000
C	-2.007588000	0.000392000	1.209416000
Ca	0.004446000	0.000004000	-0.531732000
Cl	-2.679112000	1.713321000	-2.291668000
Cl	2.422987000	2.774520000	-0.822558000
Cl	-2.065448000	2.774823000	0.912892000
Cl	1.748575000	1.716343000	2.367809000
Cl	-1.709285000	0.000948000	2.895724000
Cl	1.748394000	-1.715073000	2.368714000
Cl	-2.065124000	-2.774236000	0.914665000
Cl	2.422681000	-2.775006000	-0.821099000
Cl	-2.678904000	-1.714860000	-2.290571000
Cl	2.850231000	-0.000785000	-2.792008000

[Sr(5Cl-Cp)$_2$]

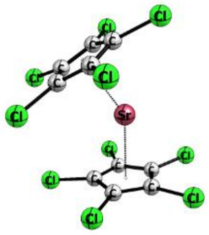

B3LYP-D3(BJ)/def2-tzvpp
Thermal correction to Gibbs Free Energy = 0.005700
Thermal correction to Enthalpy = 0.095093
Sum of electronic and thermal Free Energies = −5014.304521
Sum of electronic and thermal Enthalpies = −5014.215128
N_{imag} = 0

C	-2.199002000	-0.965708000	0.824074000
C	-2.110502000	0.390628000	1.205022000
C	-2.428010000	1.179848000	0.079678000
C	-2.716625000	0.312764000	-0.993895000
C	-2.574468000	-1.012160000	-0.534405000
C	2.427578000	-1.179988000	0.080287000
C	2.716456000	-0.313633000	-0.993804000
C	2.574821000	1.011610000	-0.535073000
C	2.199383000	0.966086000	0.823444000
C	2.110420000	-0.389992000	1.205191000
Sr	-0.000011000	0.000307000	-0.633844000
Cl	2.417547000	-2.897635000	0.016197000
Cl	-2.698252000	-2.431641000	-1.499911000
Cl	3.007440000	-0.789697000	-2.623506000
Cl	-3.007786000	0.787779000	-2.623874000
Cl	2.698734000	2.430469000	-1.501493000
Cl	-2.418252000	2.897464000	0.014482000
Cl	1.916602000	2.321389000	1.837862000
Cl	-1.741204000	0.975207000	2.773792000
Cl	1.741120000	-0.973516000	2.774348000
Cl	-1.915939000	-2.320313000	1.839335000

[Ba(5Cl-Cp)$_2$]

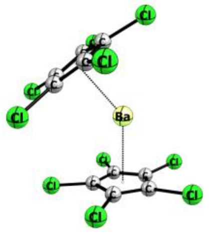

B3LYP-D3(BJ)/def2-tzvpp
Thermal correction to Gibbs Free Energy = 0.004525
Thermal correction to Enthalpy = 0.095116
Sum of electronic and thermal Free Energies = −5009.096271
Sum of electronic and thermal Enthalpies = −5009.005680
N_{imag} = 0

C	-2.550049000	-1.186724000	0.148115000
C	-2.176074000	-0.420111000	1.269700000
C	-2.282763000	0.941646000	0.919952000
C	-2.727272000	1.013723000	-0.414742000
C	-2.894325000	-0.300521000	-0.890659000
C	2.730307000	-0.999592000	-0.447196000
C	2.888088000	0.326305000	-0.892912000
C	2.544328000	1.186089000	0.168004000
C	2.180302000	0.391406000	1.273169000
C	2.292248000	-0.961179000	0.891010000
Ba	-0.001186000	0.003271000	-0.754454000
Cl	-1.719566000	-1.034890000	2.803873000
Cl	1.962526000	-2.322348000	1.883442000
Cl	2.869261000	-2.411004000	-1.423124000
Cl	-2.514526000	-2.902235000	0.038416000
Cl	3.197628000	0.808806000	-2.517946000
Cl	-3.219011000	-0.743285000	-2.523915000
Cl	2.500529000	2.903482000	0.099092000
Cl	-2.866876000	2.448681000	-1.355455000
Cl	-1.939886000	2.277579000	1.941816000
Cl	1.732137000	0.967600000	2.824553000

[Be(5Br-Cp)$_2$]

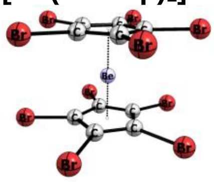

B3LYP-D3(BJ)/def2-tzvpp
Thermal correction to Gibbs Free Energy = 0.002222
Thermal correction to Enthalpy = 0.092382
Sum of electronic and thermal Free Energies = −26137.813497
Sum of electronic and thermal Enthalpies = −26137.723337
N_{imag} = 0

C	1.062448000	-0.544202000	1.660716000
C	0.852333000	0.856088000	1.654770000
C	-0.544147000	1.088910000	1.651142000
C	-1.197292000	-0.167239000	1.654765000
C	-0.204438000	-1.176716000	1.660780000
C	0.544215000	-1.088875000	-1.651141000

C	-0.852323000	-0.856106000	-1.654728000
C	-1.062423000	0.544235000	-1.660689000
C	0.204484000	1.176712000	-1.660794000
C	1.197278000	0.167258000	-1.654809000
Be	-0.001111000	-0.000551000	0.000049000
Br	-3.044803000	-0.444797000	1.713033000
Br	-2.184499000	-2.165643000	-1.714827000
Br	-0.511596000	-3.018740000	1.732117000
Br	1.379030000	-2.760561000	-1.702011000
Br	2.719310000	-1.405764000	1.732056000
Br	3.044792000	0.444836000	-1.712550000
Br	2.184531000	2.165606000	1.714329000
Br	0.511615000	3.018737000	-1.731813000
Br	-1.378978000	2.760586000	1.702001000
Br	-2.719297000	1.405791000	-1.732342000

[Mg(5Br-Cp)$_2$]

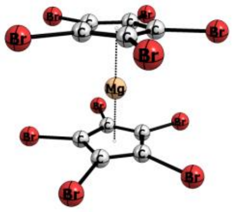

B3LYP-D3(BJ)/def2-tzvpp
Thermal correction to Gibbs Free Energy = −0.002407
Thermal correction to Enthalpy = 0.091745
Sum of electronic and thermal Free Energies = −26323.192747
Sum of electronic and thermal Enthalpies = −26323.098594
N_{imag} = 0

C	-1.135244000	0.456236000	1.970696000
C	-0.760739000	-0.911713000	1.991393000
C	0.656149000	-0.977938000	1.998207000
C	1.157050000	0.348791000	1.980789000
C	0.050071000	1.235312000	1.964267000
C	-0.656076000	0.977997000	-1.998219000
C	0.760682000	0.911946000	-1.993254000
C	1.135283000	-0.456040000	-1.971856000
C	-0.049904000	-1.235289000	-1.963112000
C	-1.156886000	-0.348925000	-1.978931000
Mg	-0.000025000	-0.000013000	0.000011000
Br	0.138012000	3.108442000	1.942747000
Br	1.932404000	2.375827000	-2.014402000
Br	2.965845000	0.843760000	1.983227000
Br	2.889824000	-1.118113000	-1.962670000
Br	1.686978000	-2.543971000	2.029208000
Br	-0.137964000	-3.108479000	-1.940254000
Br	-1.932724000	-2.375374000	2.009702000
Br	-2.965526000	-0.844216000	-1.978524000
Br	-2.889900000	1.118138000	1.960107000
Br	-1.687006000	2.543926000	-2.029142000

[Ca(5Br-Cp)$_2$]

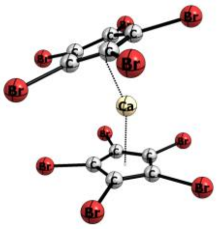

B3LYP-D3(BJ)/def2-tzvpp
Thermal correction to Gibbs Free Energy = −0.005714
Thermal correction to Enthalpy = 0.091778
Sum of electronic and thermal Free Energies = −26800.663606
Sum of electronic and thermal Enthalpies = −26800.761098
N_{imag} = 0

C	2.149368000	0.971457000	0.730254000
C	2.075717000	-0.373576000	1.154090000
C	2.282753000	-1.197485000	0.027033000
C	2.488843000	-0.362619000	-1.091882000
C	2.405868000	0.977528000	-0.657253000
C	-2.282346000	1.197499000	0.029162000
C	-2.488742000	0.364623000	-1.091178000
C	-2.406222000	-0.976299000	-0.658847000
C	-2.149678000	-0.972692000	0.728658000
C	-2.075590000	0.371583000	1.154804000
Ca	-0.000007000	-0.000036000	-0.474410000
Br	2.484625000	2.500470000	-1.758033000
Br	-2.657547000	0.938856000	-2.874906000
Br	2.657887000	-0.933761000	-2.876579000
Br	-2.485364000	-2.497312000	-1.762264000
Br	2.222067000	-3.074944000	0.006985000
Br	-1.949296000	-2.488709000	1.816222000
Br	1.813992000	-0.962948000	2.914712000
Br	-1.813821000	0.957852000	2.916454000
Br	1.948701000	2.485547000	1.820440000
Br	-2.221236000	3.074967000	0.012373000

[Sr(5Br-Cp)$_2$]

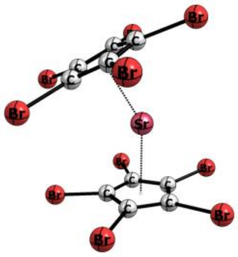

B3LYP-D3(BJ)/def2-tzvpp
Thermal correction to Gibbs Free Energy = −0.010285
Thermal correction to Enthalpy = 0.091589
Sum of electronic and thermal Free Energies = −26153.887245
Sum of electronic and thermal Enthalpies = −26153.785372
N_{imag} = 0

C	-2.250994000	-0.981085000	0.763546000
C	-2.164220000	0.364062000	1.180993000
C	-2.435574000	1.183738000	0.066238000
C	-2.696820000	0.346705000	-1.037244000

C	-2.581704000	-0.990576000	-0.606602000
C	2.435179000	-1.183725000	0.068393000
C	2.696722000	-0.348660000	-1.036508000
C	2.582039000	0.989393000	-0.608149000
C	2.251291000	0.982345000	0.762004000
C	2.164084000	-0.362057000	1.181745000
Sr	0.000012000	-0.000114000	-0.622066000
Br	-1.978897000	-2.492971000	1.843400000
Br	-2.662851000	-2.513635000	-1.710117000
Br	-1.821139000	0.958861000	2.926838000
Br	-2.902575000	0.912657000	-2.821914000
Br	-2.370154000	3.062410000	0.036252000
Br	1.979544000	2.496154000	1.839256000
Br	2.663535000	2.510522000	-1.714294000
Br	1.820976000	-0.953764000	2.928636000
Br	2.902242000	-0.917711000	-2.820222000
Br	2.369305000	-3.062423000	0.041651000

[Ba(5Br-Cp)$_2$]

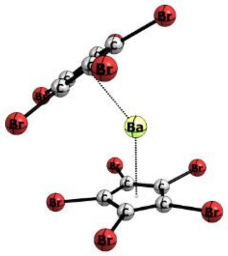

B3LYP-D3(BJ)/def2-tzvpp
Thermal correction to Gibbs Free Energy = −0.010264
Thermal correction to Enthalpy = 0.091578
Sum of electronic and thermal Free Energies = −26148.676703
Sum of electronic and thermal Enthalpies = −26148.574861
N_{imag} = 0

C	-2.473310000	-1.139510000	0.433738000
C	-2.269109000	0.000176000	1.236614000
C	-2.473438000	1.139603000	0.433405000
C	-2.814553000	0.703884000	-0.861231000
C	-2.814468000	-0.704210000	-0.861027000
C	2.675574000	-1.139019000	-0.180341000
C	2.927659000	-0.000176000	-0.968869000
C	2.675712000	1.138901000	-0.180635000
C	2.272090000	0.704944000	1.096951000
C	2.272008000	-0.704682000	1.097133000
Ba	0.015615000	0.000067000	-0.747909000
Br	-1.874740000	0.000470000	3.070881000
Br	-2.274170000	-2.926028000	0.981273000
Br	-2.274421000	2.926303000	0.980394000
Br	-2.972858000	1.801269000	-2.383398000
Br	-2.972748000	-1.802063000	-2.382854000
Br	1.828894000	-1.811621000	2.547762000
Br	2.712055000	-2.922550000	-0.777811000
Br	1.829046000	1.812307000	2.547275000
Br	2.712314000	2.922262000	-0.778602000
Br	3.265386000	-0.000441000	-2.821821000

[Be(5I-Cp)$_2$]

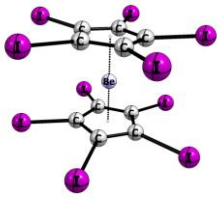

B3LYP-D3(BJ)/def2-tzvpp
Thermal correction to Gibbs Free Energy = −0.012179
Thermal correction to Enthalpy = 0.089534
Sum of electronic and thermal Free Energies = −3374.040358
Sum of electronic and thermal Enthalpies = −3373.938645
N_{imag} = 0

C	-0.396726000	-1.094087000	1.688369000
C	0.997441000	-0.840830000	1.638306000
C	1.188598000	0.563195000	1.632829000
C	-0.086367000	1.180875000	1.681624000
C	-1.068709000	0.156571000	1.715452000
C	-1.188677000	-0.562837000	-1.633109000
C	-0.997190000	0.841068000	-1.638188000
C	0.397014000	1.094021000	-1.688819000
C	1.068928000	-0.156760000	-1.715334000
C	0.086185000	-1.180694000	-1.680922000
Be	-0.000309000	-0.000470000	0.000039000
I	-0.451766000	3.215296000	1.859422000
I	3.015309000	1.544652000	1.684789000
I	-3.107827000	0.435309000	1.979305000
I	-1.295130000	-2.954054000	1.882924000
I	2.493366000	-2.276710000	1.702014000
I	3.107916000	-0.436191000	-1.979230000
I	0.451208000	-3.215221000	-1.858640000
I	-3.015438000	-1.544158000	-1.686220000
I	1.295360000	2.953998000	-1.883224000
I	-2.493031000	2.277056000	-1.701167000

[Mg(5I-Cp)$_2$]

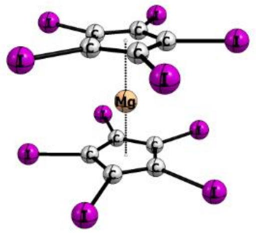

B3LYP-D3(BJ)/def2-tzvpp
Thermal correction to Gibbs Free Energy = −0.011300
Thermal correction to Enthalpy = 0.089069
Sum of electronic and thermal Free Energies = −3559.422738
Sum of electronic and thermal Enthalpies = −3559.322369
N_{imag} = 0

C	1.004801000	-0.712924000	-1.970091000
C	-0.351449000	-1.137231000	-1.992624000
C	-1.174572000	0.021491000	-2.000589000
C	-0.325975000	1.161725000	-1.983748000
C	1.020635000	0.708260000	-1.964440000

C	1.174679000	-0.020840000	2.000845000
C	0.350461000	1.137169000	1.992465000
C	-1.005680000	0.712576000	1.969784000
C	-1.019899000	-0.708588000	1.965271000
C	0.327035000	-1.161694000	1.984671000
Mg	0.000059000	0.003107000	-0.000099000
I	2.673144000	-1.954288000	-1.976580000
I	2.717046000	1.910201000	-1.965193000
I	-0.946582000	3.146165000	-2.011467000
I	-1.015976000	-3.107389000	-2.032268000
I	-3.252813000	0.043910000	-2.055608000
I	3.252878000	-0.041848000	2.056823000
I	0.949458000	-3.145304000	2.011787000
I	1.013028000	3.108289000	2.030161000
I	-2.674936000	1.952091000	1.980377000
I	-2.715265000	-1.912524000	1.96181500

[Ca(5I-Cp)$_2$]

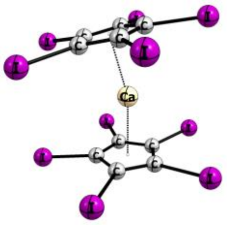

B3LYP-D3(BJ)/def2-tzvpp
Thermal correction to Gibbs Free Energy = −0.016798
Thermal correction to Enthalpy = 0.089175
Sum of electronic and thermal Free Energies = −4036.990815
Sum of electronic and thermal Enthalpies = −4036.884842
N_{imag} = 0

C	2.196466000	0.898782000	0.909062000
C	2.011606000	-0.405979000	1.423945000
C	2.201488000	-1.324687000	0.365307000
C	2.506000000	-0.588219000	-0.803310000
C	2.502207000	0.786151000	-0.467150000
C	-2.218265000	1.325824000	-0.340006000
C	-2.258928000	0.413616000	-1.420422000
C	-2.341138000	-0.893699000	-0.885856000
C	-2.352841000	-0.789219000	0.524506000
C	-2.278180000	0.582727000	0.861996000
Ca	0.034269000	-0.008954000	-0.307429000
I	2.016252000	2.673338000	1.980760000
I	2.717080000	2.371858000	-1.800717000
I	2.696524000	-1.373399000	-2.723819000
I	1.573600000	-0.890692000	3.398331000
I	1.963178000	-3.389469000	0.482751000
I	-1.983334000	3.389136000	-0.488208000
I	-2.287433000	1.371299000	2.786532000
I	-1.994917000	0.894188000	-3.430746000
I	-2.266020000	-2.667421000	-1.975292000
I	-2.444286000	-2.376057000	1.867391000

[Sr(5I-Cp)$_2$]

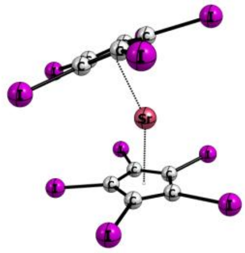

B3LYP-D3(BJ)/def2-tzvpp
Thermal correction to Gibbs Free Energy = −0.019303
Thermal correction to Enthalpy = 0.088980
Sum of electronic and thermal Free Energies = −3390.113029
Sum of electronic and thermal Enthalpies = −3390.004746
N_{imag} = 0

C	-2.373635000	-1.243958000	0.255686000
C	-2.274736000	-0.166440000	1.164923000
C	-2.424904000	1.035191000	0.435869000
C	-2.624373000	0.700054000	-0.921685000
C	-2.593238000	-0.708693000	-1.033098000
C	2.582772000	-1.036470000	-0.323574000
C	2.704924000	0.165102000	-1.057192000
C	2.477817000	1.242336000	-0.171369000
C	2.219972000	0.707143000	1.110350000
C	2.284304000	-0.702237000	1.016082000
Sr	0.014038000	0.003982000	-0.528141000
I	-2.139045000	-3.259748000	0.722874000
I	-2.576314000	-1.788658000	-2.816336000
I	-2.054085000	-0.323461000	3.227261000
I	-2.321812000	2.961555000	1.218800000
I	-2.681050000	2.048571000	-2.510376000
I	2.634277000	-2.963113000	-1.114977000
I	2.023835000	-2.046829000	2.582703000
I	2.902800000	0.312287000	-3.127395000
I	2.343094000	3.253304000	-0.699292000
I	1.860622000	1.804138000	2.841518000

[Ba(5I-Cp)$_2$]

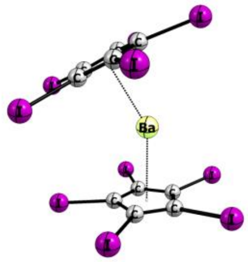

B3LYP-D3(BJ)/def2-tzvpp
Thermal correction to Gibbs Free Energy = −0.019634
Thermal correction to Enthalpy = 0.088977
Sum of electronic and thermal Free Energies = −3384.901172
Sum of electronic and thermal Enthalpies = −3384.792560
N_{imag} = 0

C	-2.467002000	-0.899359000	0.799774000
C	-2.343037000	0.451176000	1.193896000
C	-2.592668000	1.259092000	0.063601000

C	-2.877334000	0.409493000	-1.026740000
C	-2.798077000	-0.924465000	-0.571706000
C	2.577522000	-1.260865000	0.083128000
C	2.862086000	-0.450050000	-1.036370000
C	2.804257000	0.898803000	-0.623338000
C	2.485844000	0.921345000	0.751032000
C	2.349435000	-0.414224000	1.189397000
Ba	-0.002575000	0.009191000	-0.656439000
I	1.952699000	-1.030517000	3.136412000
I	2.229921000	2.624840000	1.919382000
I	2.365981000	-3.332950000	0.071712000
I	3.050735000	-1.113855000	-3.002826000
I	2.943625000	2.562849000	-1.869184000
I	-3.093970000	1.007505000	-3.011420000
I	-2.937648000	-2.629977000	-1.759638000
I	-2.188825000	-2.561505000	2.021105000
I	-1.925662000	1.134125000	3.114268000
I	-2.394252000	3.330798000	-0.019349000
